# Supplementary material for: The ecological origins of snakes as revealed by skull evolution
Source: Nat Commun. 2018 Jan 25;9:376. doi: 10.1038/s41467-017-02788-3 (PMC5785544; doi:10.1038/s41467-017-02788-3)
Supplement: Supplementary file 1 — Supplementary Information [file 41467_2017_2788_MOESM1_ESM.pdf]

The ecological origins of snakes as revealed by skull evolution

Filipe O. Da Silva, Anne-Claire Fabre, Yoland Savriama, Joni Ollonen, Kristin Mahlow, Anthony Herrel, Johannes Müller & Nicolas Di-Poï

Contact email: nicolas.di-poi@helsinki.fi

Supplementary Note 1: phylogeny, specimen collection, and geometric morphometrics

Squamate phylogenies, sources of phylogenetic data, and hypotheses for the origin of snakes

To include a large dataset of squamate specimens, including extant, fossil, and embryonic taxa (see details below as well as Fig. 1 (main text) and Supplementary Fig. 1), we used a composite phylogenetic hypothesis based on the most recent molecular as well as combined molecular and morphological studies on squamate evolution[1-5], as described in many other morphometric studies[6-8]. In short, the phylogenetic position of fossil taxa was first set based on the most-inclusive combined molecular and morphological phylogenetic analyses containing the highest number of phenotypic and molecular characters (691 morphological characters and 46 genes) for squamates[3], and fossils were then incorporated into the nexus file containing a large number of extant species analyzed using molecular phylogenetics[1,5]. As extinct madtsoiid snakes were not included in the study by Reeder *et al.*[3], we used other recent phylogenetic analyses that also combined phenotypic and genotypic characters to infere snake phylogeny[2,4]. Other published fossils such as *Coniophis*[9], *Najash*[10], and *Tetrapodophis*[11] were not included in our study because of their highly incomplete skulls not suitable for global skull shape analysis (see also below). The phylogenetic positions of some fossils such as *Dinilysia* and mosasauroids are remarkably instable across previous studies, but are most commonly recovered as crown snake[3] and sister group of crown snakes[3,4], respectively, in the most recent combined molecular and morphological phylogenetic inferences (Supplementary Fig. 1), so we adopted these phylogenetic positions in our main morphometric analyses. Branch length information was not considered in analyses where fossil data were included, as branch lengths of fossils and extant species were not co-estimated in our composite phylogeny. For such analyses, another high-level phylogenetic tree containing fewer species but combining molecular, morphological, and fossil information was used[3]. Importantly, both several alternative composite hypotheses and different recent combined molecular and morphological phylogenetic trees[2-4], including or not fossil data, mosasauroids, and branch length information, as well as with *Dinilysia* as sister-taxon to all extant snakes[2], were also tested to increase the robustness of our study.

Sampling, source of data, and quality control parameters

All specimens used in this study are summarized in Supplementary Tables 1 and 2. Sources of data include 2D high-quality photographs of dry as well as cleared and alcian blue/alizarin red-

stained (C&S) skulls, accurate drawings made by anatomists and taxonomists and published in highly respected publications and/or specialized book on squamate anatomy (see Supplementary Tables 1-2), as well as 3D computed tomography (CT) scans. Specimens were sampled from published literature, the Digital Morphology Database (DigiMorph), reptile colonies at the University of Helsinki and Tropicario zoo (Finland), as well as from collections at different museums (Finnish Museum of Natural History, Finland; Museum für Naturkunde Berlin, Germany; Museum of Comparative Zoology at Harvard University, USA; American Museum of Natural History, USA). Newly produced high-resolution CT scans were generated at three different imaging facilities: University of Eastern Finland, Finland (Skyscan 1172 microCT); Museum für Naturkunde Berlin, Germany (Phoenix nanotom CT); University of Helsinki (Skyscan 1272 microCT and Phoenix Nanotom 180). Surface rendering of skulls and 3D segmentation of cranial bones were done using the software Amira 5.5.0 (Visualization Sciences Group). Newly produced 2D photographs of skulls were taken in our laboratory at the University of Helsinki and at the Museum für Naturkunde Berlin.

We investigated patterns of skull shape disparity using 100 extant squamate species (125 skulls) in our 3D analysis (Supplementary Tables 1-2). We further expanded our dataset to 326 species (408 skulls) for the 2D analysis, by covering all major lineages of extant squamates (Supplementary Tables 1-2). This number of extant squamate species sampled represents approximately 3% of total Squamata, based on the total number of known extant species reported in the August 15th, 2016 version of the Reptile Database (http://www.reptile-database.org). In addition to skull data newly produced in this study, we performed extensive data mining across the vast literature on squamate skulls to add any published information. Our sampling covered most families and morphological transitions in lizards and snakes (see Fig. 1 and Supplementary Fig. 1), and only 6 extant families[1,5] (3 for snakes and 3 for lizards) could not be sampled because of scarce literature or rarity of samples in museum collections. In addition, we analyzed well-preserved lepidosaurian fossil skulls available from the literature, including 5 species related to snakes (7 skulls), 18 species related to lizards (19 skulls), and 1 outgroup species (Supplementary Fig. 1 and Supplementary Table 1). Unfortunately, most snake fossils reported so far are highly fragmented or only represented by vertebral elements, thus hampering their inclusion in our study (see e.g., Fossilworks: http://fossilworks.org). Similarly, many lizard fossils are fragmented or crushed, thus limiting the sampling. For developmental studies, we traced the ontogenetic trajectories based on 84 embryos, including 51 lizard (36 species), 31 snake (18 species), and 2 *Sphenodon punctatus* (Sphenodontidae, outgroup) specimens.

The last decades of research on squamate skull evolution produced a large range of morphological descriptions of adult skulls and a valuable photographic record of adult, embryonic, and fossil data positioned in standardized 2D lateral views. In complement to 3D analyses, we then

included these unique data in our study by conducting 2D analyses, with the goals of incorporing fossil data but also to better evaluate the high phenotypic diversity across different squamate families and lineages. This allowed us to perform a large-scale synthesis of skull shape diversity throughout squamates. Importantly, for the consistency of our data, we also tested for the correct lateral positioning of the skull in our 2D analyses by incorporating whenever possible more than one specimen per extant species or more than one reconstruction or original skull picture per squamate fossil, thus circumventing taphonomic problems or misinterpretations in reconstructed skulls. The accuracy and correct positioning of skulls in lateral view were further improved by controlling for shape outliers in the software MorphoJ v1.06[12], and by comparing different sources of data (including 2D and 3D skull information) for the same species and/or genus when available. In a very few species, we observed unexpected outliers because of skull mispositioning; these skull pictures were either excluded or recaptured after repositioning of the specimens in lateral view, thus improving the quality of our data. Finally, because of bone movements in open and closed mouth positions, only species with closed mouths were selected. Most importantly, we show here that the results and conclusions obtained from both 2D and 3D data converge. These quality and congruence checks ensured the quality of our datasets for morphometric investigations and, ultimately, the reliability of our results.

Description of landmarks in 2D and 3D analyses and acquisition of shape data

To describe skull shape variation, 61 and 20 landmarks were selected for 3D (Supplementary Fig. 2c-j and Supplementary Table 4) and 2D (Supplementary Fig. 2a-b and Supplementary Table 3) data, respectively. Because of the limited 3D information for squamate fossils, we only landmarked original photographs and reconstructions of well-preserved fossils in our 2D analysis. In addition, we only selected landmark points on embryonic skulls showing at least some ossification pattern in all cranial bones, thus ensuring homology throughout ontogeny. The earliest embryos landmarked were around mid-embryonic development after oviposition (20-30 days post-oviposition (dpo)), but the majority of collected embryos were at more advanced stages in the last 1/4 of development. The definition of our landmarks followed the terminology described previously for squamates[13-17]. We digitized the configuration of 3D and 2D landmarks using Amira 5.5.0 (Visualization Sciences Group) and TpsDig v2.17[18], respectively. All data were scaled by voxel or pixel size in the respective 3D and 2D software packages.

Geometric morphometric methods for analysis of morphological variation

We used geometric morphometrics to quantify shape variation in squamate skulls. In geometric morphometrics, shape is defined as all the geometric information that remains when position, scale, and rotational effects are removed from an object[19]. Data were scaled, translated, and

oriented via a generalized Procrustes analysis (GPA) superimposition method[19,20]. Scaling was done by calculating the centroid size, which is estimated as the square root of the sum of squared distances of each landmark from their centroid[19]. To describe the patterns of shape variation, we used covariance matrices of shape data after Procrustes superimposition to calculate the principal axes of shape variation with principal component analysis (PCA)[21]. This method summarizes the multidimensional shape data through independent orthogonal axes of main shape variation. The graphic ordination plot of two or more PC axes produces an empirical morphospace where observed data are scattered regarding their shape variations to the mean shape (0.0). The PCs are ordered by decreasing variance and the first two dimensions often account for most of the variance in the data. Because of the large shape variations in our dataset (Supplementary Figs 3 and 4), we further checked for deformation by performing a regression through the origin for distance in tangent space onto Procrustes distance (in radians) using the software tpsSmall v1.29[22], and found it to be non-significant (correlations > 0.99; p-value < 0.0001). Importantly, the influence of size or evolutionary allometry was also tested using a multivariate regression analysis of independent-contrasts of shape (Procrustes coordinates) on size (centroid size; see details in part 2.4 below), and all our analyses were conducted before (original shape space) and after allometric correction[23]. Methods for estimation and graphical representation of shape variation using thin-plate spline interpolation function (TPS) have been described previously[24,25]. All the analyses described in this section were performed using the software package MorphoJ v1.06[12].

Supplementary Note 2: phylomorphospace and evolution of skull shape, size, and ecology

Skull shape evolution and phylomorphospace

We used both unweighted and weighted squared-change parsimony algorithms[54], as implemented in MorphoJ v1.06[12], to estimate skull shape and size evolution in a phylogenetic context. Importantly, all ancestral reconstructions were performed using different phylogenies allowing us to include different species numbers (277 species[1], 147 species[3], and 60 species[2] species) and/or branch lengths and to address the phylogenetic uncertainty of some fossils such as *Dinilysia*[2,3] and mosasauroids[3,4] (see above). Note that weighted squared-change parsimony is equivalent to maximum likelihood algorithms when branch lengths are included. Importantly, very similar morphospaces and skull shape predictions for both most recent common ancestor (MRCA) of crown snakes and MRCA of snakes and their sister group were obtained for all phylogenies tested, independently of the number of species, presence/absence of mosasauroids, and position of *Dinilysia* (as crown snake or sister-taxon to all extant snakes, see above), and none of the fossils used in this study were found near the

estimated ancestral skull shapes (see, e.g., Fig. 2). In addition, the skull shape of the MRCA of crown snakes was systematically recovered at negative PC2 values and PC1 positive values, where most fossorial snake and lizard species are distributed (see, e.g., Supplementary Fig. 5). We then generated a phylomorphospace (Fig. 3 and Supplementary Fig. 3) by plotting the phylogenetic tree onto the morphospace delimited by main PCs and characterized by a series of lines (phylogenetic branches) connecting the shape of the operational taxonomic units (OTUs) to their MRCA. The presence of a phylogenetic signal was calculated based on the Procrustes coordinates and residual shape for all specimens, using a multivariate *K*-statistic[26] in the R-package geomorph v3.0.5[27] available on the CRAN package repository (https://cran.r-project.org/web/packages/). We obtained a significant phylogenetic structure in our data with (*K*-value=0.53; p-value=0.001) and without (*K*-value=0.85; p-value=0.001) allometric-correction.

Ecological analysis

Habitat preferences were gathered from published literature and/or reptile databases such as the IUCN Red List of Threatened Species, the Reptile Database, and the Global Invasive Species Database (see Supplementary Tables 1 and 2). Habitat preferences were first simplified into five main categories (aquatic, terrestrial, leaf-litter, fossorial, arboreal; see Supplementary Tables 1 and 2) and then plotted onto the morphospace generated from extant adult skulls (Fig. 4 and Supplementary Fig. 5). Interestingly, the morphospace already indicates that the skull shape of specialized fossorial lizards (Rhineuridae, Bipedidae, Trogonophiidae, Amphisbaenidae, Scincidae, Gymnopthalmidae, Dibamidae, Pygopodidae, and Anguidae) and fossorial snakes (Scolecophidia, Anomochilidae, and Uropeltidae) fits to a limited range of skull shapes at negative PC2 values, with only limited overlap with other ecologies when considering the two main PCs (Fig. 4 and Supplementary Fig. 5). As species share some part of their evolutionary history, they cannot be treated as independent data points. Thus, we conducted ecological analyses in a phylogenetic framework[28] using the high-level phylogenies described above for extant species. We first tested if skull shape differed among habitat modes with MANOVAs and phylogenetic MANOVAs[29] using the 'aov.phylo' function in the R-package geiger v2.0.6[30] on the first 11 PCs (accounting for more than 90% of total shape variation). Simulations of new shape variables on the tree were performed under a Brownian motion-model (using 1000 simulations) to create an empirical null distribution against which the *F*-value from the original data could be compared. For MANOVA, we used Wilks' statistic as a multivariate test. A large influence of ecology on skull shapes was observed before (nsim=1000, F=12.62, p-value=0.0001) and after phylogenetic correction (nsim=1000, F=7.5, p-value=0.001), and *post hoc* pairwise comparisons revealed significant differences between the fossorial ecology and all other habitat modes (p-value=7.1e-6 for fossorial versus terrestrial; p-value=0.0009 for fossorial versus leaf

litter) as well as between the terrestrial and aquatic habitat modes (p-value=0.00102). We next used pairwise discriminant function analysis (DFA) to estimate the proportional chance of identifying correct ecologies based on shape parameters through a cross-validation prodedure[31] in MorphoJ v1.06. The reliability of the discrimination was assessed by a leave-one-out cross-validation, also implemented in MorphoJ v1.06, which provides a parametric T-square test for the statistical difference between group means set *a priori*. By inspecting the habitat modes near the reconstructed MRCA of crown snakes and MRCA of snakes and their sister group (Fig. 4), we found that the mean shape of terrestrial lizard species was significantly different from other lizard ecologies such as arboreal (T-square=108.02; p-value=0.005), aquatic (T-square=118.30; p-value=0.009), and leaf litter (T-square=122.24; p-value=0.005). In addition, as expected from the morphospace, the mean skull shape of terrestrial lizards (or from other ecologies) was significantly distinct from that of fossorial lizards (T-square=1211.03; p-value<0.0001) or fossorial snakes (T-square=2967.03; p-value<0.0001). Similarly, a fossorial or terrestrial ecology could be correctly assigned in 100% of the cases based on skull shape variables, in contrast to other ecologies. Importantly, to account for allometric effects, all those tests were also run using the size-corrected regression residuals. To quantify convergent evolution in the different ecological categories, the distance-based convergence measures C1-C4 were computed using the R-package convevol v1.1, as described in Stayton 2015[32]. Significance was assessed in the same package using 1000 evolutionary simulations along the phylogeny according to a Brownian motion-model[32]. Coherent with the phylogenetic trends observed in the phylomorphospace, these analyses confirm the significant convergence of fossorial snake and lizard species (Supplementary Table 5). Finally, to predict the ecologies of the MRCAs of Toxicofera, snakes and their sister group, and crown snakes, a linear discriminant analysis (LDA) was performed on the PC scores of the generalized Procrustes superimposition using the "lda" function from the R-package MASS v7.3-47 (Supplementary Table 6). A leave-one-out cross validation procedure removes one specimen at a time and predicts its classification using LDA function computed on all remaining specimens. At the end, a classification accuracy of the ecological category of each specimen is given by the percentage of specimens correctly assigned by the cross-validated LDA. Finally, the MRCAs relative to the origin and diversification of snakes are added to the analysis and assigned to an ecological category. Coherent with the morphospace (Fig. 4), these analyses predict with high confidence the fossorial origin of the MRCA of crown snakes and the terrestrial origin of the MRCA of snakes and their sister group (independently of the size correction). Importantly, and consistent with a surface-terrestrial-to-fossorial transition, the MRCA of Toxicofera was also predicted as terrestrial (55%; Supplementary Table 6).

Skull shape and ecology of fossils

Snake fossils available in the literature are mostly represented by incomplete skull or vertebral fragments[9,11,33]. Good representative snakes with intact skull that could be included in our study include the well-preserved terrestrial/fossorial *Dinilysia*[34,35] and two fairly well-preserved terrestrial Madtsoiidae (*Wonambi*[36] and *Yurlunggur*[37]). The skull of *Najash*[10], *Coniophis*[9], and *Kataria*[38] are too incomplete to be used in our study. The marine Simoliophiidae snakes *Pachyrhachis*[39], *Haasiophis*[40], and *Eupodophis*[41] have crushed skulls, but precise reconstructions of the first two species were added to our analyses[42,43]. The terrestrial *Sanajeh*[44] is also crushed and does not have any reconstruction. The back skull of *Tetrapodophis amplectus,* recently identified as a putative four-legged snake from the Early Cretaceous[11], is also crushed and lacks the quadrate bone. Interestingly, while the skull shape of all analyzed snake fossils was found to be relatively similar (despite strong ecological differences), none of the fossils were positioned near our skull shape reconstructions for both MRCA of crown snakes and MRCA of snakes and their sister group (Fig. 3) in all phylogenies tested (see above). Fossil lizards were located in the cloud composed of non-fossorial species in the morphospace analysis (Supplementary Fig. 4), except for *Sineoamphisbaena hexatabularis*[45], which showed extreme positive PC1 values, and the rhineurid fossils *Spathorhynchus natronicus*[46] and *Plesiorhineura hatcherii*[47], located in the specialized fossorial shape space (Supplementary Fig. 4); the latter data confirm the fossorial ecology of these fossils. The necrosaur *Eosaniwa koehni*[48] as well as the two large marine lizards *Mosasaurus hoffmanni*[49] and *Plotosaurus bennisoni*[16] were located at extreme positive PC2 values (Supplementary Fig. 4).

Skull size evolution in squamates

Allometry, or shape changes associated with size variation, is a factor that can contribute substantially to the integration of morphological traits. As mentioned above, the influence of allometry was tested using a multivariate regression analysis of independent-contrasts of skull shape (Procrustes coordinates) on independent-contrasts of log-transformed centroid size[50] (Supplementary Tables 7-8); statistical significance was assessed using a permutation test (10000 permutations) against the null hypothesis of total independence. Allometric tests were also adjusted for phylogenetic signal[51] (see Results section of main text) based on phylogenetically independent contrasts[28], which is equivalent to distance-based phylogenetic generalized least square (D-PGLS) regression[52]. As shown in Supplementary Table 9, allometric tests revealed significant correlations between shape and size both in our 2D and 3D datasets (p-values<0.0001). However, the multivariate regression accounts for only a small part of the total shape variation (less than 15% and 9% for 3D and 2D data, respectively; Supplementary Table 9), thus indicating that allometric corrections should have limited effect in the total inference of shape patterns. Residual scores were further used to verify its relevance

to the patterns of shape distribution within morphospace. Interestingly, shape changes were almost exclusively observed for some scolecophidian species along PC2 (and not PC1); with allometric correction, the latter species show increased PC2 values and slightly overlap with non-fossorial lizard species (Supplementary Fig. 7). Significance allometric association was also found by analyzing scolecophidians separately (p-value=0.019). Interestingly, those changes were not observed for alethinophidian snakes or for other fossorial species like amphisbaenians. These results indicate that allometry was important in the early evolution of snakes, as the allometric association increased approximately by 2-fold from lizards to snakes (Supplementary Table 9). Importantly, however, similar fossorial skull shapes were obtained by amphisbaenians through a process independent of allometry. This argues for an independent origin of fossoriality between those two groups, as also supported by phylogenetic studies[1,3,53]. As previously mentioned, the size-corrected regression residuals were also used in other analyses, including those testing for ecological patterns (see above).

We further evaluated size diversification within squamates by estimating skull size evolution (centroid size) using unweighted squared-change parsimony algorithms[54] in MorphoJ v1.06. Importantly, these studies revealed a consistent larger size of the MRCA of snakes and their sister group when compared to the MRCA of crown snakes, regardless of the dataset (2D or 3D, with or without lizards; see e.g., Fig. 5 and Supplementary Fig. 6), again indicating the importance of body size in the early evolution of snakes. In addition, while the MRCA of alethinophidian snakes and sister fossil taxa initially increased in size, alethinophidians later diversified into a broad range of sizes, ranging from secondarily minituarized *Anomochilus* (with size comparable to scolecophidian snakes) to large pythons and boas.

Supplementary Note 3: Heterochrony and the origin of snakes

Quantification of ontogenetic trajectories

To better understand cranial ontogeny and the impact of heterochrony on skull evolution in snakes, we performed PCA and allometric analyses in a unique embryonic dataset covering 50% of squamate families (including some rare fossorial amphisbaenian and scolecophidian specimens), using similar methods as above. Ontogenetic trajectory vectors were then obtained by connecting younger-to-older specimens for each species (Supplementary Figs 8 and 9), and their geometric properties (path length, direction, angle) were quantified and compared based on the approach described by Collyer and Adams[55,56], using the “trajectory.analysis” function in geomorph v3.0.5 package[27]. This method allows the examination of phenotypic evolution as vectors of phenotypic change between two ontogenetic points, by comparing vectors across pairs of snakes and lizards. Small differences in the magnitude and direction of vectors between lizards and snakes would indicate

similar ontogenetic and evolutionary ontogenetic trajectories, a prerequisite for testing heterochrony hypotheses[6,57,58]. Procrustes distances between pairs of phenotypic trajectories were used to assess potential differences between lizard and snake species, and statistical significance was determined by a random permutation procedure of 1000 iterations (Supplementary Table 10). Because of the large variation of embryonic character development and the difficulty of comparing squamate embryos at early and intermediate stages based on published staging tables (including the 'standard event system'[59]), ontogenetic trajectories were only quantified between two equivalent points: late embryo (stage 10) and adult (Supplementary Fig. 8b). Indeed, the latest stage of snake and lizard development (stage 10, as defined in the model organism *Boaedon fuliginosus*[60]) is more easily identifyable based on external characters such as the presence of pigmented eyes, inverted hemipenes, and well-patterned scales covering the body (including the dorsal aspect of the head)[59,60.]

Testing of heterochrony hypotheses

As both the angle and direction of trajectories were not statistically different in our dataset (Supplementary Figs 8 and 9 and Table 10), different heterochronic hypotheses were tested using multivariate regression of shape (Procrustes coordinates) onto log-centroid size as a proxy for developmental time[6,57,58]; the slope, length, and angle between descendent trajectories (snakes) in relation to ancestor trajectories (lizards) were then compared and quantified (Fig. 6a, Supplementary Fig. 10 and Supplementary Table 11) to predict global peramorphosis (acceleration: faster rate of development; hypermorphosis: delayed offset; predisplacement: earlier onset) or paedomorphosis (neoteny: slower rate of development; progenesis: earlier offset; postdisplacement: later onset) changes as described in the literature[57,58]. Strikingly, the steeper slope and angle (Supplementary Table 11) of snake ontogenetic trajectories, when compared to lizards (Fig. 6a and Supplementary Fig. 10b), indicates a faster rate of development in snakes and a global acceleration model. Importantly, to support that evolutionary scenario, we also confirmed the lack of significant differences in the duration of embryonic development in lizards and snakes (Supplementary Tables 12-13) by comparing species with well documented incubation and/or gestation periods[61,62], using analysis of variance (ANOVA). Because incubation times in oviparous species are nearly always reported as time to hatching after egg deposition, these times are not strictly comparable with gestation periods in viviparous species. Consequently, oviparous and viviparous species were analyzed both together and separately. Importantly, the temperature of egg incubation is also well known to affect the total duration of development in ectotherms such as squamates[62], so only oviparous species with incubation temperatures around 30 +/- 1^0C were used. Finally, only one representative species per genus was used to get a more representative sample. In total, 128 different species across 19 and 8 families of lizards and snakes, respectively, could be used in this study

(Supplementary Table 12). The null hypothesis (no differences in developmental time in squamates) was tested by assessing the p-value. The validity of the global acceleration model was further tested by comparing the offset of ossification in the skull of late pre-hatchling lizard and snake embryos (stage 10), with the expectation that snake skulls would show a higher degree of ossification than those of lizards. For this study, we focused on the ossification pattern of the parietal and frontal bones, the last two bones to complete ossification in squamates and thus serving as an excellent proxy for developmental time[63,64]. We used a discrete, numerical scale approach to rank the degree of ossification of these bones based on the classification scheme already developed for lizards[63,64], but by expanding ossification level details (Supplementary Table 14). Comparison of snake and lizard embryos (including species with strictly similar incubation periods like *Pogona vitticeps*, *Crotaphopeltis hotamboia*, *Pantherophis guttatus*, *Boaaedon fuliginosus*) confirmed our hypothesis of acceleration, as shown by the systematically more advanced ossification degree of both parietal and frontal bones in snakes, when compared to lizards (Fig. 6b and Supplementary Table 14). Especially, the parietal bone of lizard embryos shows a large unossified skull roof (fontanella) at stage 10 independently of the incubation/gestation periods of tested species, which is coherent with the observed late closure of this bone at post-embryonic stages[63-65]. Interestingly, the clear exceptions in our data are scolecophidian snakes that develop similarly to lizards and show largely unossified skulls at late developmental stages (Fig. 6b and Supplementary Table 14) or even at juvenile and adult stages (e.g., *Myriopholis cairi* and *Indotyphlops braminus*). These observations contrast with the expectation that the rate of ossification is similar in all snakes[66], but are well coherent with previous reports showing that at least some scolecophidians never complete skull ossification[15]. The skull shape similarities between scolecophidians and lizards suggest that scolecophidians may have retained the ancestral rate of development found among lizards.

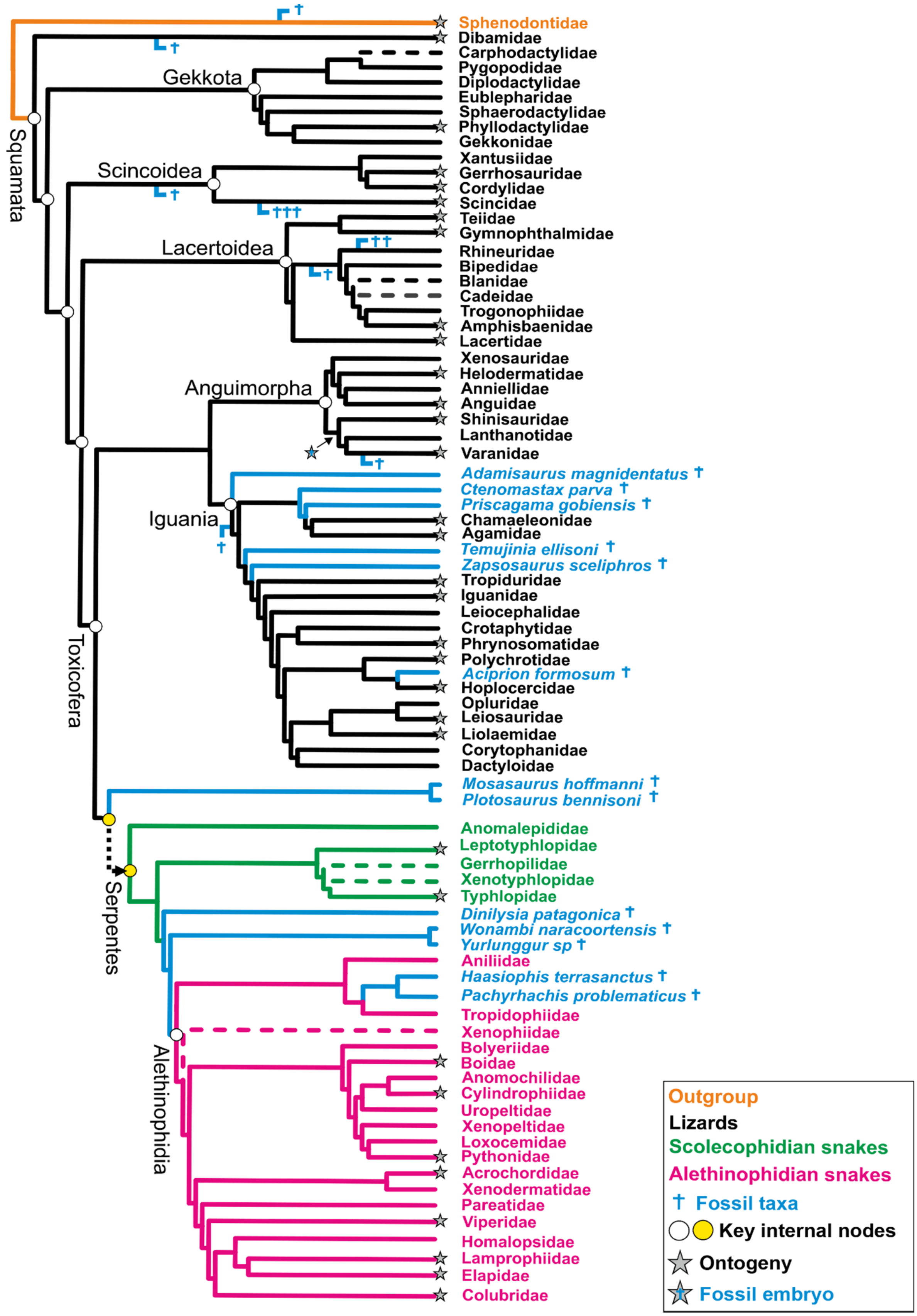

Supplementary Figure 1 | Composite phylogenetic tree of extant and fossil squamate species used in this study, adapted from the most inclusive and recent studies on squamate evolution[1-5], and rooted using Sphenodontidae (tuatara). Dashed lines represent families not analyzed. Positions of sampled fossils and ontogenies are indicated by blue crosses and stars, respectively. See Supplementary Tables 1 and 2 for a complete list of species.

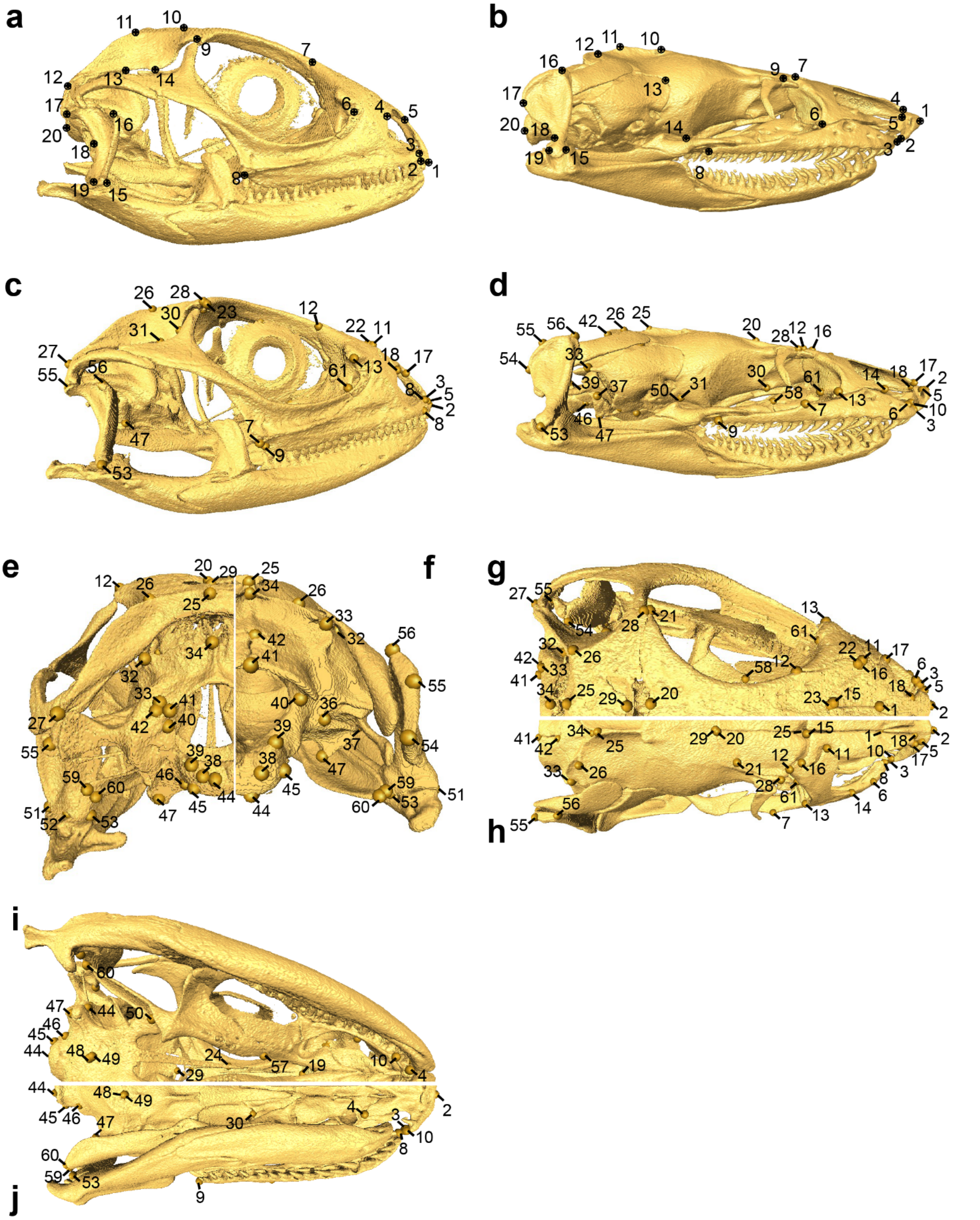

Supplementary Figure 2 | 2D (a,b) and 3D (c-j) landmark points on the skull of the lizard *Chalarodon madagascariensis* (a, c, e, g, i) and the snake *Loxocemus bicolor* (b, d, f, h, j) in lateral (a-d), posterior (e, f), dorsal (g, h), and ventral (i, j) views.

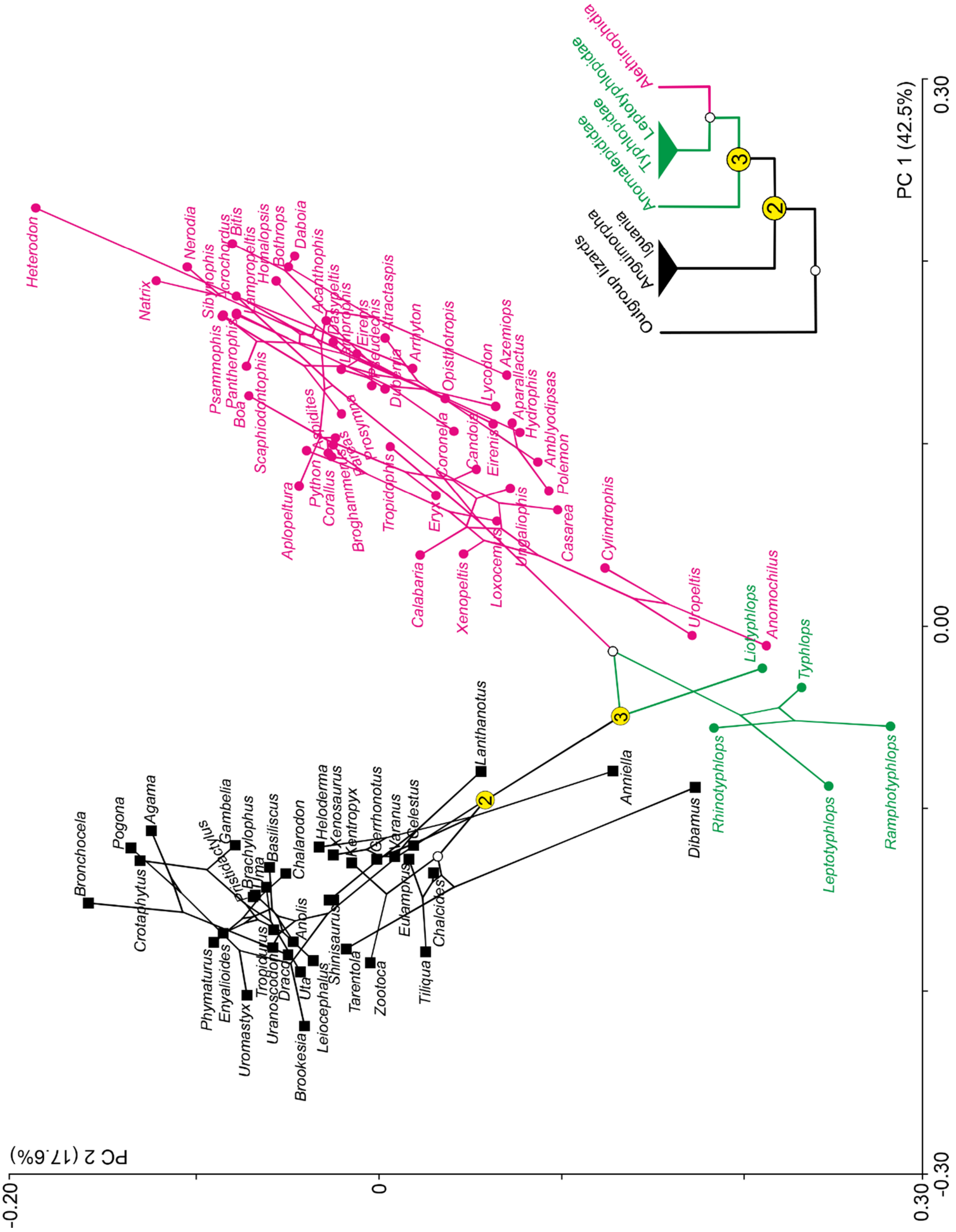

Supplementary Figure 3 | Phylomorphospace of extant squamate species for which 3D data were available (with species names indicated, see also Supplementary Tables 1 and 2). The estimated lizard-to-snake transition took place between reconstructed internal nodes 2 and 3 (yellow nodes). Numbers in brackets indicate the percentage of variance explained by each of the critical PC axes. The bottom right cladogram represents a simplified phylogenetic tree of major lineages and ancestral nodes shown in the phylomorphospace.

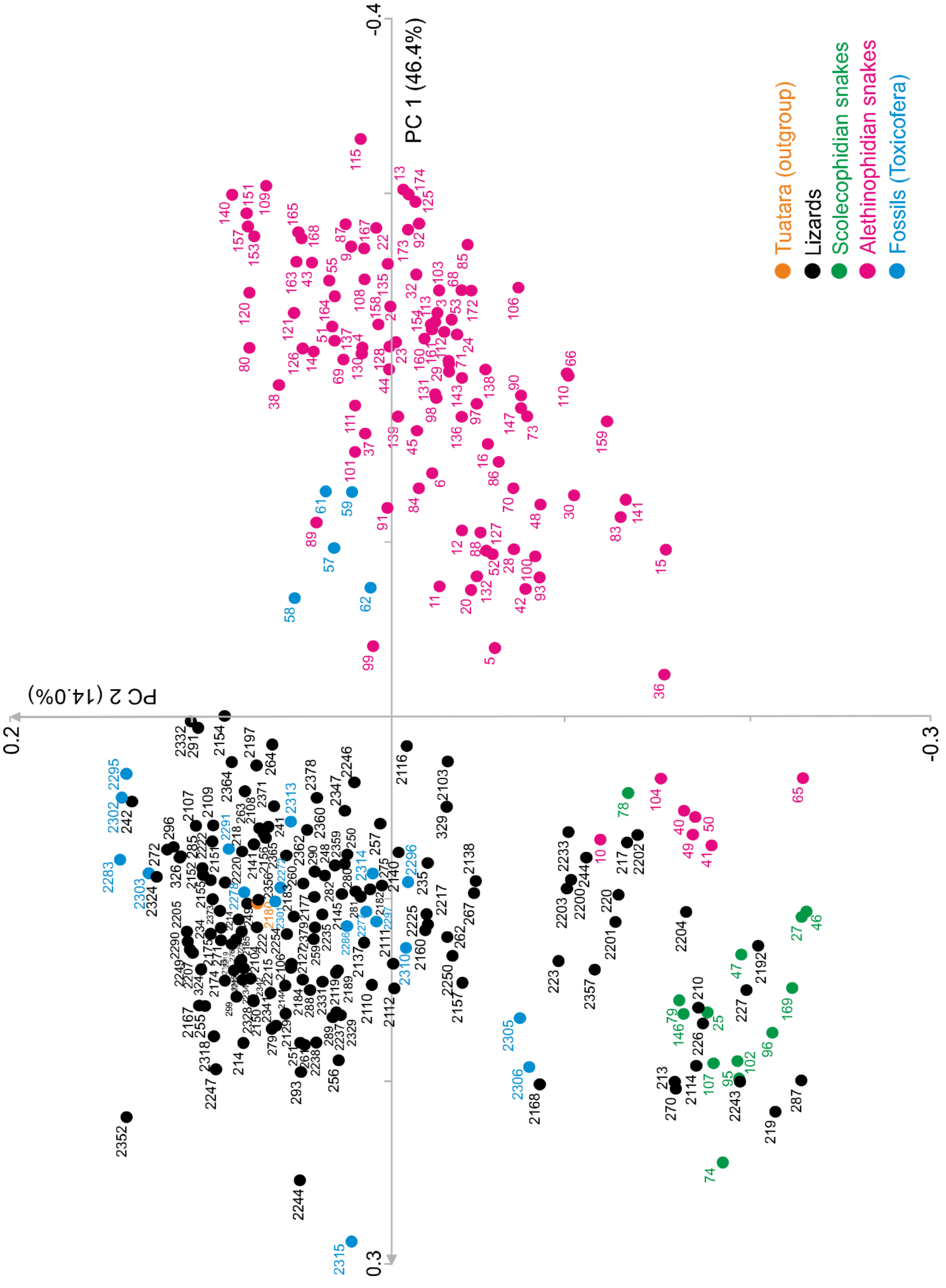

Supplementary Figure 4 | Morphospace of extant and fossil squamate species for which 2D data were available. For the complete list of species (with given ID numbers) see Supplementary Tables 1 and 2.

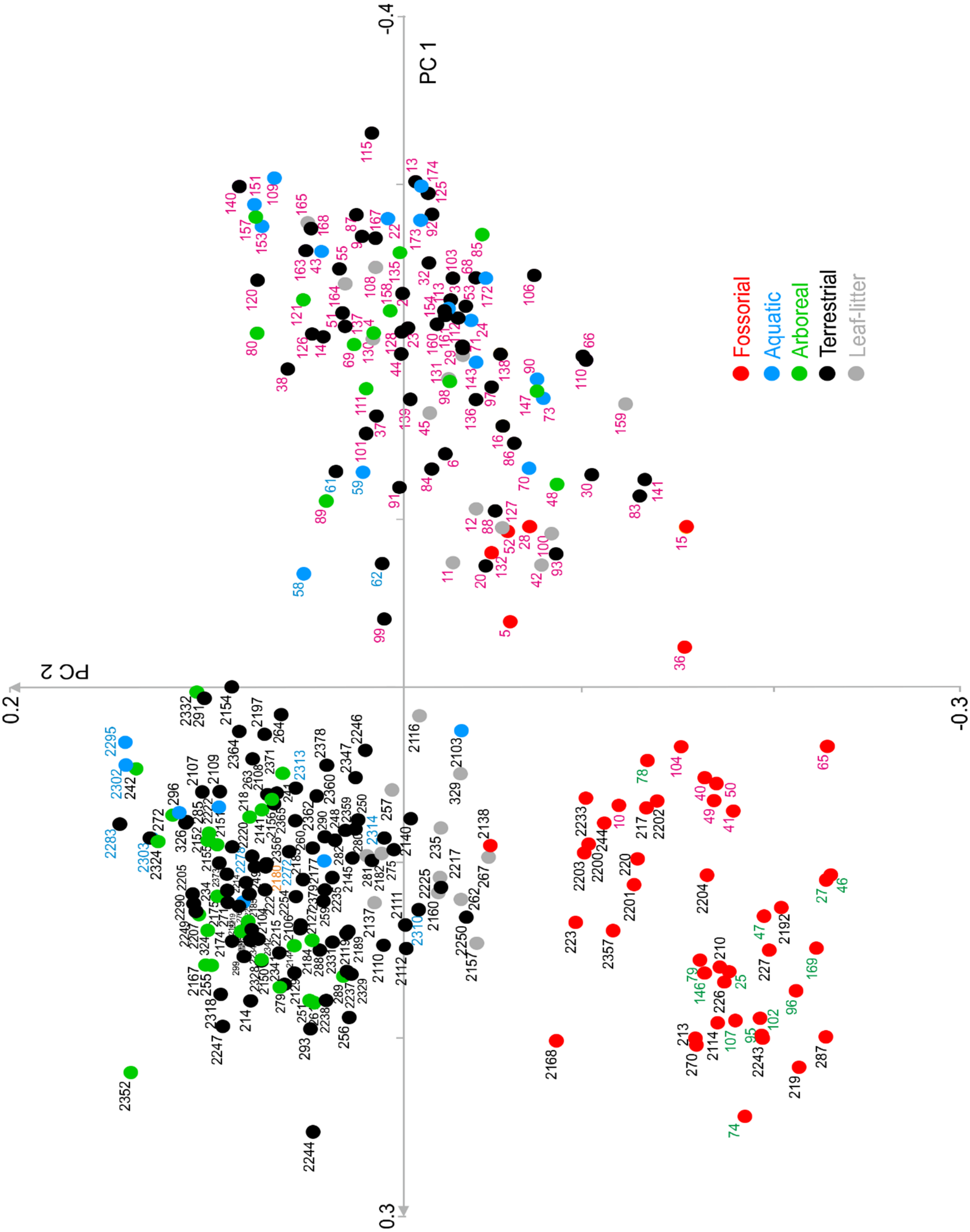

Supplementary Figure 5 | Morphospace of extant and fossil squamate species for which 2D data were available. Only Toxicofera fossils with known ecologies are shown. The color code for circles reflects different ecologies (see legend in bottom right corner), while the color code for numbers indicates lizard (black), scolecophidian (green), alethinophidian (red), fossil (blue), or tuatara outgroup (orange) species. For the complete list of species (with given ID numbers) see Supplementary Tables 1 and 2.

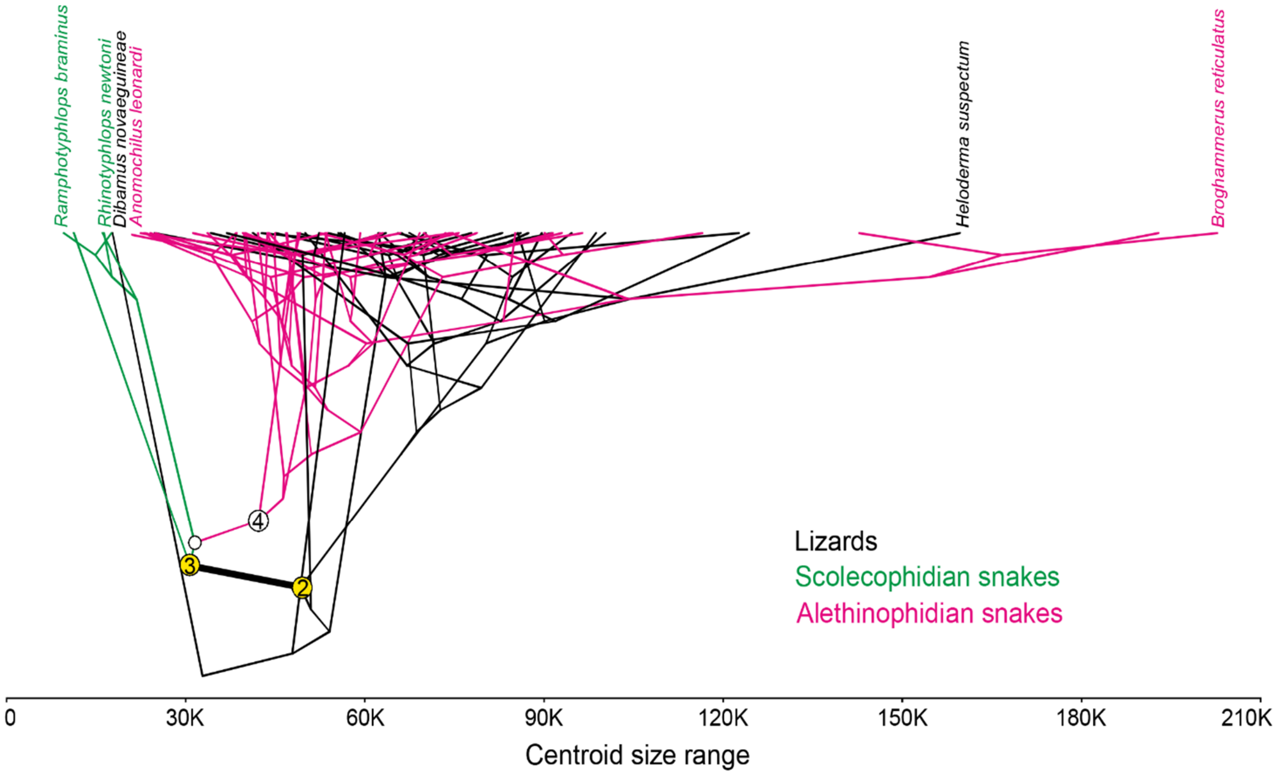

Supplementary Figure 6 | Centroid size variation (in mm) of 3D skulls from extant lizard, scolecophidian, and alethinophidian species. Names of species showing extreme centroid size values in the different groups are indicated. The estimated lizard-to-snake transition took place between reconstructed internal nodes 2 and 3 (yellow nodes), as indicated by the thick bold line. See the complete list of centroid sizes in Supplementary Tables 7 and 8.

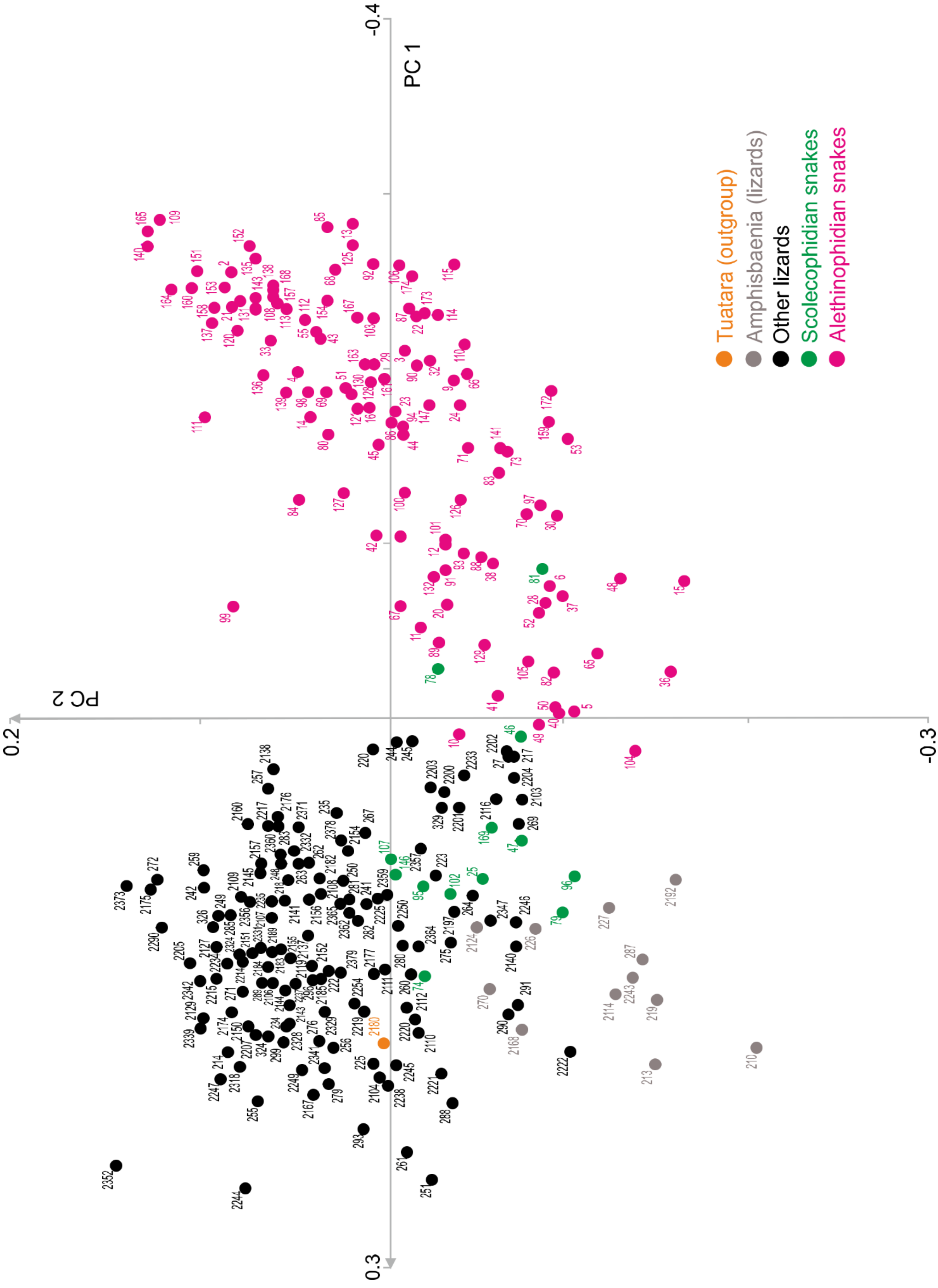

Supplementary Figure 7 | Morphospace after allometry correction of extant squamate species for which 2D data were available. For the complete list of species (with given ID numbers) see Supplementary Tables 1 and 2.

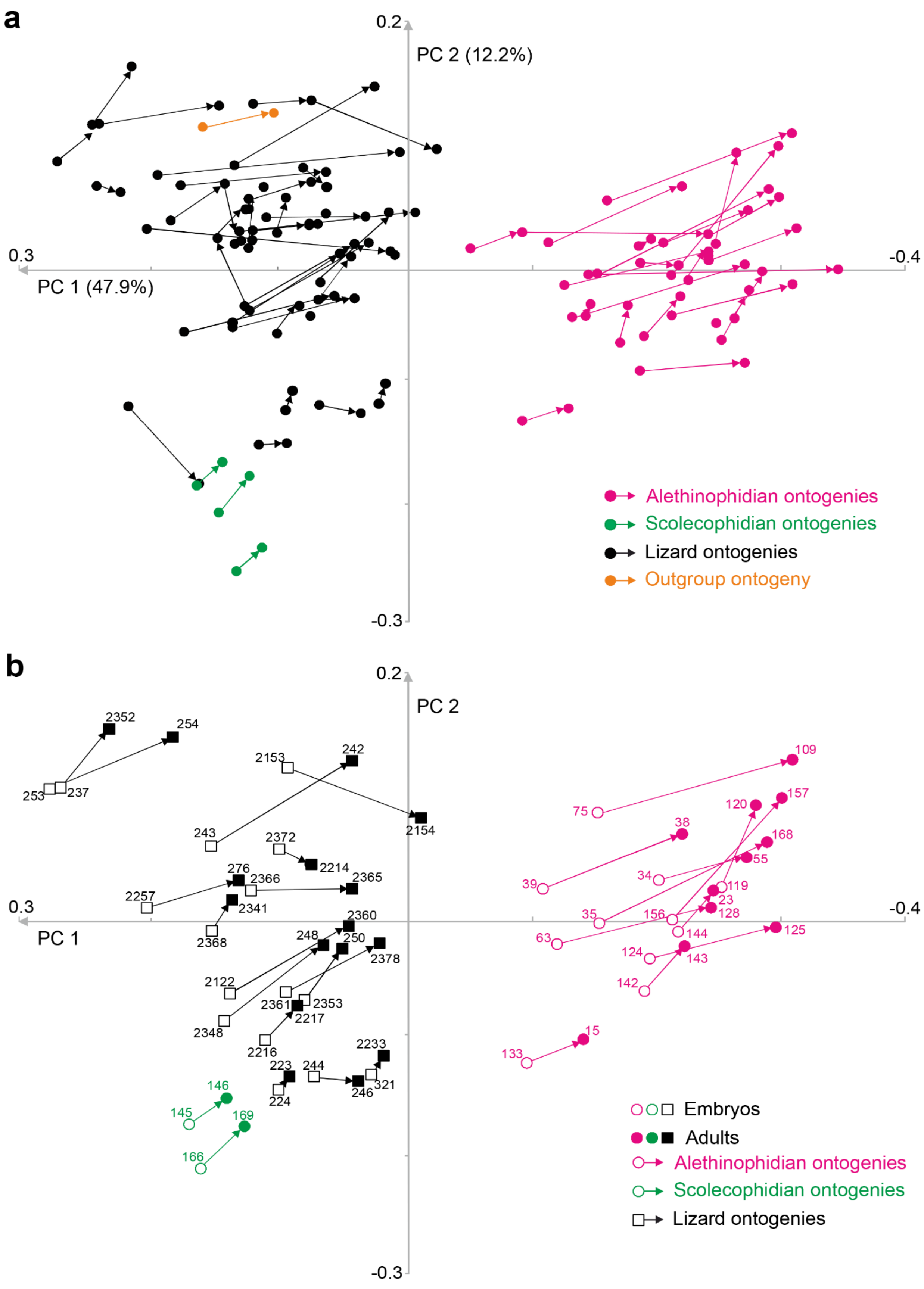

Supplementary Figure 8 | Ontogenetic trajectories for all lizard and snake embryos for which 2D data were available (**a**) or only between stage 10 embryos and adults (**b**). Arrowheads in indicate the direction of shape change throughout ontogeny. ID numbers from specimens used in (**b**) can be found in Supplementary Tables 1 and 2.

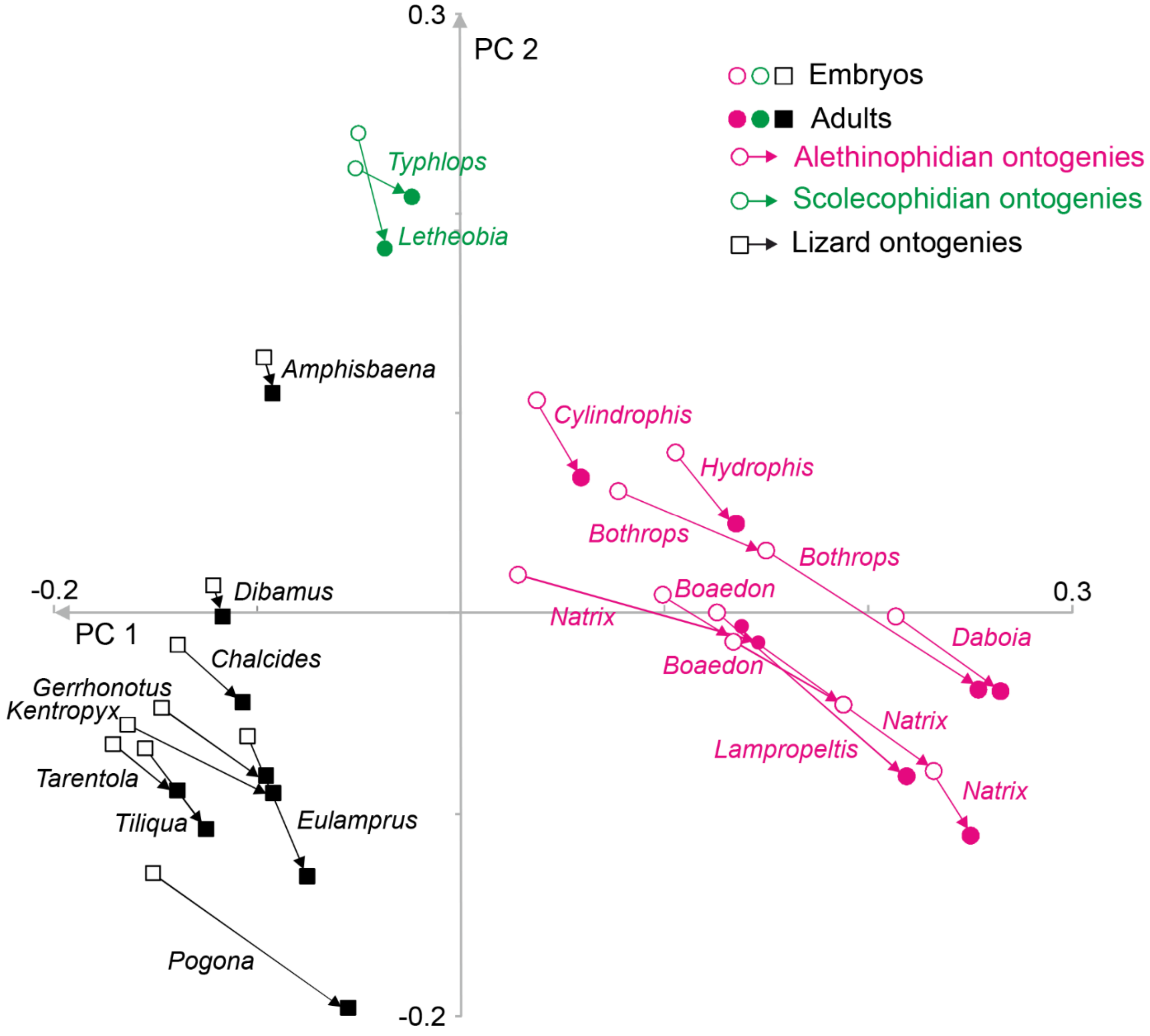

Supplementary Figure 9 | Morphospace of squamate ontogenies for which 3D data were available (with species names indicated). For the complete list of species see Supplementary Tables 1 and 2.

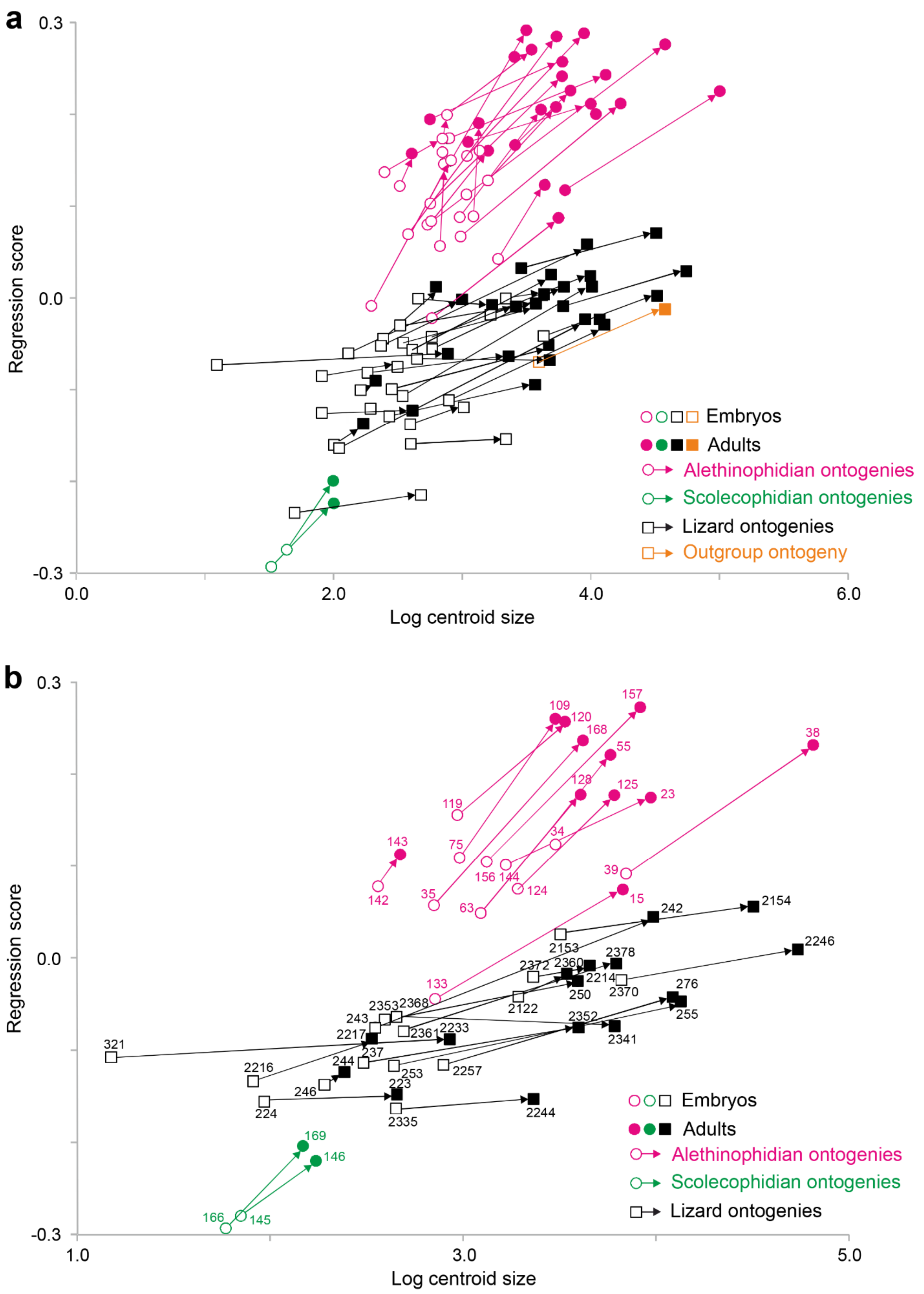

Supplementary Figure 10 | Regression analysis of 2D shape (regression score) on log-centroid size for all squamate ontogenies (**a**), or only for ontogenies between stage 10 embryos and adults (**b**). ID numbers from specimens used in (**b**) can be found in Supplementary Tables 1 and 2.

Supplementary Table 1 | List of identifiers and classifiers for all lizard and outgroup species used in the study. Species are classified by family names. Legend: ID number; Group (lizard = L, snake = S, outgroup = O); Species name; Source (type of data and/or origin, including computed tomography (CT) scan, cleared and stained (C&S) skull, picture, and/or accurate drawing); Source reference (published, unpublished, or newly produced); Type (ex = extant species, fo = fossil); Family (reptile families); 3D analysis (species analyzed (Y=yes) or not (N=no) in the 3D data); Ecology (main ecologies of species: Aq=aquatic, SAq=semi-aquatic, M=marine, F=fossorial (species living and foraging underground), LL=leaf litter (terrestrial species living under vegetation layers or surface debris), T=terrestrial (species adapted for surface locomotion and foraging), TSax=terrestrial saxicolous (species living on or among rocks), Ar=Arboreal (species adapted for locomotion between tree branches or bushes), SAr=Semi-arboreal, ?=no ecological information); Ecology reference (reference number); Stage (ad=adult, j=juvenile, em(st10)=late (stage 10) embryo, em=early or intermediate embryo). New specimens produced by this work and embryonic specimens are highlighted with bold font and gray shading, respectively.

ID	Group	Species	Source	Source (ref)	Type	Family	3D analysis	Ecology	Ecology (ref)	Stage
326	L	*Agama agama*	CT scan (Digimorph)	16	ex	Agamidae	No	T	123,124	ad
607	L	*Agama hispida*	**CT scan (ZMB 25567)**	**This work**	ex	Agamidae	Yes	T	123,124	ad
2324	L	*Bronchocela jubata*	Picture	Savalli (unpublished)	ex	Agamidae	No	Ar	125	ad
610	L	*Bronchocela jubata*	CT scan (**ZMB 36897**)	**This work**	ex	Agamidae	Yes	Ar	125	ad
242	L	*Calotes emma*	CT scan (Digimorph)	16	ex	Agamidae	No	SAr	126,127	ad
243	L	*Calotes versicolor*	Acurate drawing	120	ex	Agamidae	No	Ar	53	em(st10)
272	L	*Draco quinquefasciatus*	CT scan (Digimorph)	16	ex	Agamidae	No	Ar	128	ad
611	L	*Draco volans*	**CT scan (LUOMUS 1346)**	**This work**	ex	Agamidae	Yes	Ar	128	ad
296	L	*Hydrosaurus pustulatus*	Biolid picture repository	Zuber (unpublished)	ex	Agamidae	No	SAq	129	ad
2332	L	*Hypsilurus boydii*	Acurate drawing	122	ex	Agamidae	No	Ar	126,130	ad
2107	L	*Leiolepis belliana*	CT scan (Digimorph)	16	ex	Agamidae	No	T	53,131,132	ad
2108	L	*Leiolepis triploida*	CT scan (Digimorph)	Digimorph (unpublished)	ex	Agamidae	No	T	133,134	ad
2129	L	*Moloch horridus*	CT scan (Digimorph)	Digimorph (unpublished)	ex	Agamidae	No	T	126	ad
2151	L	*Physignathus cocincinus*	CT scan (Digimorph)	16	ex	Agamidae	No	SAr	135	ad
604	L	*Pogona barbata*	**CT scan (ZMB 54559)**	**This work**	ex	Agamidae	Yes	T	126	ad
2154	L	*Pogona vitticeps*	**CT scan (Lab)**	**This work**	ex	Agamidae	No	T	126	ad
2153	L	*Pogona vitticeps*	**CT scan (Lab)**	**This work**	ex	Agamidae	No	T	126	em(st10)
2337	L	*Pogona vitticeps*	**CT scan (Lab PV144)**	**This work**	ex	Agamidae	Yes	T	126	em
2336	L	*Pogona vitticeps*	**CT scan (Lab PV106)**	**This work**	ex	Agamidae	Yes	T	126	em
2249	L	*Saara hardwickii*	CT scan (Digimorph)	Digimorph (unpublished)	ex	Agamidae	Yes	T	136,137	ad
210	L	*Amphisbaena alba*	CT scan (Digimorph)	16	ex	Amphisbaenidae	No	F	138,139	ad
2350	L	*Amphisbaena caeca*	**CT scan (AMNH 13237)**	**This work**	ex	Amphisbaenidae	Yes	F	138	em
2243	L	*Amphisbaena darwinii*	C&S	50	ex	Amphisbaenidae	No	F	138	ad
211	L	*Amphisbaena darwinii*	C&S	50	ex	Amphisbaenidae	No	F	138	em
213	L	*Amphisbaena fuliginosa*	CT scan (Digimorph)	16	ex	Amphisbaenidae	No	F	138	ad
219	L	*Amphisbaena kingii*	CT scan (Digimorph)	Digimorph (unpublished)	ex	Amphisbaenidae	No	F	138	ad
2114	L	*Amphisbaena microcephalum*	CT scan (Digimorph)	Digimorph (unpublished)	ex	Amphisbaenidae	No	F	138	ad

287	L	*Geocalamus acutus*	CT scan (Digimorph)	Digimorph (unpublished)	ex	Amphisbaenidae	No	F	138	ad
2124	L	*Loveridgea ionidesii*	CT scan (Digimorph)	Digimorph (unpublished)	ex	Amphisbaenidae	Yes	F	138	ad
2345	L	*Anguis fragilis*	**CT scan (LUOMUS)**	**This work**	ex	Anguidae	No	T/LL	140	em(st10)
2351	L	*Anguis fragilis*	**CT scan (ZMB RE29)**	**This work**	ex	Anguidae	Yes	T/LL	140	em
248	L	*Celestus enneagrammus*	CT scan (Digimorph)	16	ex	Anguidea	Yes	T	53,141	ad
2348	L	*Celestus costatus*	**CT scan (MCZ R166783)**	**This work**	ex	Anguidae	No	T	53,141	em(st10)
2359	L	*Diploglossus lessonae*	Acurate drawing	15	ex	Anguidae	No	T	142,143	ad
275	L	*Elgaria multicarinata*	CT scan (Digimorph)	16	ex	Anguidea	No	T/LL	53,144	ad
2374	L	*Elgaria multicarinata*	C&S (fineartamerica)	Hanken (unpublished)	ex	Anguidae	No	T/LL	53,144	em
2362	L	*Gerrhonotus infernalis*	**CT scan (ZMB 1154)**	**This work**	ex	Anguidae	Yes	T	145	ad
2363	L	*Gerrhonotus infernalis*	**CT scan (AMNH 155913)**	**This work**	ex	Anguidae	Yes	T	145	em
2140	L	*Pseudopus apodus*	CT scan (Digimorph)	16	ex	Anguidea	No	T	146,147	ad
217	L	*Anniella pulchra*	CT scan (Digimorph)	16	ex	Anniellidae	Yes	F	148,149	ad
226	L	*Bipes biporus*	CT scan (Digimorph)	16	ex	Bipedidae	No	F	150	ad
227	L	*Bipes canaliculatus*	CT scan (Digimorph)	16	ex	Bipedidae	No	F	151	ad
2352	L	*Bradypodion pumilum*	Acurate drawing	15	ex	Chamaeleonidae	No	Ar	152	ad
237	L	*Bradypodion pumilum*	C&S	67	ex	Chamaeleonidae	No	Ar	152	em(st10)
609	L	*Brookesia brygooi*	CT scan (Digimorph)	16	ex	Chamaeleonidae	Yes	SAr	153	ad
251	L	*Chamaeleo calyptratus*	CT scan (Digimorph)	Digimorph (unpublished)	ex	Chamaeleonidae	No	Ar	153	ad
255	L	*Chamaeleo laevigatus*	CT scan (Digimorph)	16	ex	Chamaeleonidae	No	Ar	153	ad
254	L	*Trioceros hoehnelii*	C&S	67	ex	Chamaeleonidae	No	Ar	153	ju
253	L	*Trioceros hoehnelii*	C&S	67	ex	Chamaeleonidae	No	Ar	153	em(st10)
252	L	*Trioceros hoehnelii*	C&S	67	ex	Chamaeleonidae	No	Ar	153	em
2356	L	*Chamaesaura anguina*	**CT scan (ZMB 56421)**	**This work**	ex	Cordylidae	No	T	53,154	ad
2349	L	*Chamaesaura anguina*	**C&S (MCZ R173157)**	**This work**	ex	Cordylidae	No	T	53,154	em
260	L	*Smaug mossambicus*	CT scan (Digimorph)	16	ex	Cordylidae	No	TSax	53	ad
225	L	*Basiliscus basiliscus*	CT scan (Digimorph)	16	ex	Corytophanidae	Yes	SAq/SAr	53	ad
261	L	*Corytophanes cristatus*	CT scan (Digimorph)	16	ex	Corytophanidae	No	Ar	155,156	ad
263	L	*Crotaphytus collaris*	CT scan (Digimorph)	16	ex	Crotaphytidae	Yes	TSax	157	ad
285	L	*Gambelia wislizenii*	CT scan (Digimorph)	16	ex	Crotaphytidae	Yes	T	53	ad
218	L	*Anolis carolinensis*	CT scan (Digimorph)	16	ex	Dactyloidae	No	Ar	158	ad
608	L	*Anolis sagrei*	**CT scan (ZMB 537)**	**This work**	ex	Dactyloidae	Yes	Ar	159	ad
269	L	*Dibamus novaeguineae*	Acurate drawing	68	ex	Dibamidae	No	F	160	ad
2357	L	*Dibamus novaeguineae*	**CT scan (ZMB 33822)**	**This work**	ex	Dibamidae	Yes	F	160	ad
2358	L	*Dibamus novaeguineae*	**CT scan (ZMB 33822)**	**This work**	ex	Dibamidae	Yes	F	160	ju
2245	L	*Saltuarius cornutus*	CT scan (Digimorph)	16	ex	Diplodactylidae	No	Ar	161	ad
2328	L	*Strophurus ciliaris*	CT scan (Digimorph)	16	ex	Diplodactylidae	No	Ar	162	ad
324	L	*Aeluroscalabotes felinus*	CT scan (Digimorph)	16	ex	Eublepharidae	No	SAr	163	ad
256	L	*Coleonyx variegatus*	CT scan (Digimorph)	16	ex	Eublepharidae	No	TSax	164	ad
279	L	*Eublepharis macularius*	CT scan (Digimorph)	16	ex	Eublepharidae	No	TSax	165	ad
293	L	*Hemitheconyx caudicinctus*	CT scan (Digimorph)	Digimorph (unpublished)	ex	Eublepharidae	No	T	166	ad
2272	L	*Aciprion formosum*	CT scan (Digimorph)	16	fo	Fossil	No	T	167	ad
2314	L	*Adamisaurus magnidentatus*	Picture	69	fo	Fossil	No	T	71	ad
5000	L	*Anguimorpha embryo*	CT scan	70	fo	Fossil	No	?	70	em
2277	L	*Cryptolacerta hassiaca*	CT scan	53	fo	Fossil	No	LL	53	ad
2278	L	*Ctenomastax parva*	CT scan (Digimorph)	16	fo	Fossil	No	T	71	ad
2283	L	*Eosaniwa koehni*	Acurate drawing (reconstruction)	48	fo	Fossil	No	T	48	ad

2285	O	*Gephyrosaurus bridensis*	Acurate drawing	43	fo	Fossil	No	T	168	ad
2286	L	*Globaura venusta*	Acurate drawing	71	fo	Fossil	No	?	169	ad
2291	L	*Huehuecuetzpalli mixtecus*	Acurate drawing	72	fo	Fossil	No	?	170	ad
2295	L	*Mosasaurus hoffmanni*	Acurate drawing	49	fo	Fossil	No	M	171	ad
2296	L	*Myrmecodaptria microphagosa*	Acurate drawing	71	fo	Fossil	No	?	71	ad
2297	L	*Myrmecodaptria microphagosa*	Fossil	71	fo	Fossil	No	?	71	ad
2301	L	*Parmeosaurus scutatus*	CT scan (Digimorph)	16	fo	Fossil	No	T?	71	ad
2305	L	*Plesiorhineura hatcherii*	CT scan	47	fo	Fossil	No	F	47	ad
2302	L	*Plotosaurus bennisoni*	CT scan (Digimorph)	16	fo	Fossil	No	M	16	ad
2303	L	*Priscagama gobiensis*	Acurate drawing	73	fo	Fossil	No	T	71	ad
2315	L	*Sineoamphisbaena hexatabularis*	Acurate drawing	45	fo	Fossil	No	F	45	ad
2306	L	*Spathorhynchus natronicus*	Picture	46	fo	Fossil	No	F	46	ad
2310	L	*Temujinia ellisoni*	Fossil	71	fo	Fossil	No	T	71	ad
2313	L	*Zapsosaurus sceliphros*	Picture	71	fo	Fossil	No	T	71	ad
2318	L	*Agamura persica*	CT scan	74	ex	Gekkonidae	No	TSax	172	ad
2290	L	*Bunopus tuberculatus*	Acurate drawing	74	ex	Gekkonidae	No	T	173	ad
2331	L	*Hemidactylus frenatus*	CT scan	74	ex	Gekkonidae	No	T	174	ad
2144	L	*Phelsuma lineata*	CT scan (Digimorph)	16	ex	Gekkonidae	No	Ar	175	ad
2339	L	*Ptenopus carpi*	CT scan	74	ex	Gekkonidae	No	T	176	ad
2167	L	*Rhacodactylus auriculatus*	CT scan (Digimorph)	16	ex	Gekkonidae	No	Ar	177	ad
288	L	*Broadleysaurus major*	CT scan (Digimorph)	Digimorph (unpublished)	ex	Gerrhosauridae	No	TSax	53	ad
259	L	*Cordylosaurus subtessellatus*	CT scan (Digimorph)	16	ex	Gerrhosauridae	No	TSax	178	ad
214	L	*Gerrhosaurus skoogi*	CT scan	75	ex	Gerrhosauridae	No	T	179	ad
215	L	*Gerrhosaurus skoogi*	CT scan	75	ex	Gerrhosauridae	No	T	179	ju
2247	L	*Tracheloptychus petersi*	CT scan (Digimorph)	Digimorph (unpublished)	ex	Gerrhosauridae	No	T	53	ad
2238	L	*Zonosaurus ornatus*	CT scan (Digimorph)	16	ex	Gerrhosauridae	No	T	53	ad
223	L	*Bachia bicolor*	Acurate drawing	76	ex	Gymnophthalmidae	No	F	180	ad
224	L	*Bachia bicolor*	Acurate drawing	76	ex	Gymnophthalmidae	No	F	180	em(st10)
245	L	*Calyptommatus nicterus*	Acurate drawing	77	ex	Gymnophthalmidae	No	F	181	ad
244	L	*Calyptommatus sp.*	C&S	78	ex	Gymnophthalmidae	No	F	181	ad
246	L	*Calyptommatus sinebrachiatus*	Acurate drawing	78	ex	Gymnophthalmidae	No	F	77	em(st10)
257	L	*Colobosaura modesta*	CT scan (Digimorph)	16	ex	Gymnophthalmidae	No	LL	182	ad
283	L	*Euspondylus acutirostris*	Acurate drawing	79	ex	Gymnophthalmidae	No	LL	183	ad
2138	L	*Nothobachia ablephara*	Acurate drawing	78	ex	Gymnophthalmidae	No	F	181,182	ad
2145	L	*Pholidobolus montium*	CT scan (Digimorph)	16	ex	Gymnophthalmidae	No	T	184	ad
2137	L	*Potamites ecpleopus*	Acurate drawing	80	ex	Gymnophthalmidae	No	LL	182	ad
2136	L	*Potamites ecpleopus*	Acurate drawing	80	ex	Gymnophthalmidae	No	LL	182	ju
2157	L	*Procellosaurinus tetradactylus*	C&S	78	ex	Gymnophthalmidae	No	LL	182	ad
2160	L	*Psilophthalmus paeminosus*	C&S	78	ex	Gymnophthalmidae	No	LL	182	ad
2162	L	*Ptychoglossus bicolor*	C&S	81	ex	Gymnophthalmidae	No	LL	185	em(st10)
2176	L	*Scriptosaura catimbau*	Acurate drawing	78	ex	Gymnophthalmidae	No	F	181	ad
2217	L	*Vanzosaura rubricauda*	C&S	82	ex	Gymnophthalmidae	No	LL	182	ad
2216	L	*Vanzosaura rubricauda*	Acurate drawing	78	ex	Gymnophthalmidae	No	LL	182	em(st10)
290	L	*Heloderma horridum*	CT scan (Digimorph)	16	ex	Helodermatidae	Yes	T	186	ad
291	L	*Heloderma suspectum*	CT scan (Digimorph)	Digimorph (unpublished)	ex	Helodermatidae	No	T	187	ad
2330	L	*Heloderma suspectum*	CT scan (Digimorph)	Digimorph (unpublished)	ex	Helodermatidae	No	T	187	ju
2257	L	*Hoplocercus spinosus*	C&S	83	ex	Hoplocercidae	No	T	188	em(st10)
276	L	*Enyalioides laticeps*	CT scan (Digimorph)	16	ex	Hoplocercidae	Yes	Ar	189,190	ad
234	L	*Brachylophus fasciatus*	CT scan (Digimorph)	16	ex	Iguanidae	Yes	Ar	191	ad

264	L	*Ctenosaura pectinata*	CT scan (Digimorph)	Digimorph (unpublished)	ex	Iguanidae	No	T/SAr	192	ad
271	L	*Dipsosaurus dorsalis*	CT scan (Digimorph)	16	ex	Iguanidae	No	T	193	ad
2364	L	*Iguana iguana*	CT scan	118	ex	Iguanidae	No	Ar	194	ad
2259	L	*Iguana iguana*	C&S	83	ex	Iguanidae	No	Ar	194	em
2258	L	*Iguana iguana*	C&S	83	ex	Iguanidae	No	Ar	194	em
2234	L	*Ichnotropis capensis*	**Picture (ZMB 13943)**	**This work**	ex	Lacertidae	No	T	195	ad
299	L	*Lacerta viridis*	CT scan (Digimorph)	16	ex	Lacertidae	No	T	196	ad
298	L	*Lacerta agilis*	C&S	84	ex	Lacertidae	No	T	196	em
297	L	*Lacerta agilis*	C&S	84	ex	Lacertidae	No	T	196	em
2104	L	*Latastia longicaudata*	CT scan (Digimorph)	Digimorph (unpublished)	ex	Lacertidae	No	T	197	ad
2379	L	*Psammodromus algirus*	**CT scan (LUOMUS 1198)**	**This work**	ex	Lacertidae	No	T	198	ad
2184	L	*Takydromus formosanus*	CT scan (Digimorph)	16	ex	Lacertidae	No	SAr	199	ad
2237	L	*Takydromus sexlineatus*	**CT scan (ZMB 14567)**	**This work**	ex	Lacertidae	No	SAr	199	ad
2373	L	*Zootoca vivipara*	**CT scan (ZMB 27791)**	**This work**	ex	Lacertidae	Yes	T	199	ad
2239	L	*Zootoca vivipara*	C&S	65	ex	Lacertidae	No	T	199	em
2240	L	*Zootoca vivipara*	C&S	65	ex	Lacertidae	No	T	199	em
2103	L	*Lanthanotus borneensis*	CT scan (Digimorph)	16	ex	Lanthanotidae	Yes	SAq	53	ad
2106	L	*Leiocephalus barahonensis*	CT scan (Digimorph)	16	ex	Leiocephalidae	Yes	T	200	ad
2256	L	*Anisolepis longicauda*	C&S	83	ex	Leiosauridae	No	T	201	em
2109	L	*Leiosaurus catamarcensis*	CT scan (Digimorph)	16	ex	Leiosauridae	No	T	202	ad
2156	L	*Pristidactylus torquatus*	CT scan (Digimorph)	16	ex	Leiosauridae	Yes	T	203	ad
2214	L	*Urostrophus vautieri*	CT scan (Digimorph)	16	ex	Leiosauridae	No	T	204	ad
2372	L	*Urostrophus vautieri*	**CT scan (ZMB RE28)**	**This work**	ex	Leiosauridae	Yes	T	204	em(st10)
2119	L	*Liolaemus bellii*	CT scan (Digimorph)	16	ex	Liolaemidae	No	T	205	ad
2120	L	*Liolaemus scapularis*	C&S	85	ex	Liolaemidae	No	T	205	em
2150	L	*Phymaturus palluma*	CT scan (Digimorph)	16	ex	Liolaemidae	Yes	TSax	206	ad
249	L	*Chalarodon madagascariensis*	CT scan (Digimorph)	16	ex	Opluridae	Yes	T	207	ad
2141	L	*Oplurus cyclurus*	CT scan (Digimorph)	16	ex	Opluridae	No	SAr	208	ad
2143	L	*Petrosaurus mearnsi*	CT scan (Digimorph)	16	ex	Phrynosomatidae	No	TSax	209	ad
2244	L	*Phrynosoma platyrhinos*	CT scan (Digimorph)	16	ex	Phrynosomatidae	No	T	210	ad
2335	L	*Phrynosoma taurus*	CT scan (Digimorph)	Digimorph (unpublished)	ex	Phrynosomatidae	No	T	210	em(st10)
2174	L	*Sceloporus variabilis*	CT scan (Digimorph)	16	ex	Phrynosomatidae	No	T	211	ad
2205	L	*Uma scoparia*	CT scan (Digimorph)	16	ex	Phrynosomatidae	Yes	T	212	ad
2215	L	*Uta stansburiana*	CT scan (Digimorph)	16	ex	Phrynosomatidae	Yes	T	213	ad
2341	L	*Tarentola americana*	CT scan/Acurate drawing	86	ex	Phyllodactylidae	No	TSax/Ar	214	ad
605	L	*Tarentola mauritanica*	**CT scan (ZMB 17966)**	**This work**	ex	Phyllodactylidae	Yes	TSax/Ar	214	ad
2368	L	*Tarentola mauritanica*	**CT scan (ZMB 5)**	**This work**	ex	Phyllodactylidae	Yes	TSax/Ar	214	em(st10)
2367	L	*Tarentola mauritanica*	**CT scan (ZMB 4)**	**This work**	ex	Phyllodactylidae	Yes	TSax/Ar	214	em
2155	L	*Polychrus marmoratus*	CT scan (Digimorph)	16	ex	Polychrotidae	No	Ar	215	ad
2261	L	*Polychrus acutirostris*	C&S	83	ex	Polychrotidae	No	Ar	215	em
220	L	*Aprasia striolata*	Acurate drawing	87	ex	Pygopodidae	No	F	216	ad
267	L	*Delma borea*	CT scan (Digimorph)	16	ex	Pygopodidae	No	LL	217	ad
2116	L	*Lialis burtonis*	CT scan (Digimorph)	16	ex	Pygopodidae	No	LL	218	ad
2168	L	*Rhineura floridana*	CT scan (Digimorph)	16	ex	Rhineuridae	No	F	219	ad
2200	L	*Acontias aurantiacus*	Acurate drawing	88	ex	Scincidae	No	F	88	ad
2202	L	*Acontias cregoi*	Acurate drawing	88	ex	Scincidae	No	F	88	ad
2203	L	*Acontias lineatus*	Acurate drawing	88	ex	Scincidae	No	F	88	ad
2233	L	*Acontias meleagris*	**CT scan (ZMB 14540)**	**This work**	ex	Scincidae	No	F	220	ad

321	L	*Acontias meleagris*	C&S	114	ex	Scincidae	No	F	220	em(st10)
329	L	*Amphiglossus splendidus*	CT scan (Digimorph)	16	ex	Scincidae	No	LL	221	ad
235	L	*Brachymeles gracilis*	CT scan (Digimorph)	16	ex	Scincidae	No	LL	222,223	ad
250	L	*Chalcides ocellatus*	CT scan (Digimorph)	Digimorph (unpublished)	ex	Scincidae	Yes	T	224	ad
2353	L	*Chalcides chalcides*	**CT scan (ZMB HUB29)**	**This work**	ex	Scincidae	Yes	T	224	em(st10)
2354	L	*Chalcides chalcides*	**CT scan (ZMB HUB29)**	**This work**	ex	Scincidae	Yes	T	224	em
2355	L	*Chalcides chalcides*	**CT scan (ZMB HUB40A)**	**This work**	ex	Scincidae	Yes	T	224	em
2329	L	*Egernia depressa*	Picture	89	ex	Scincidae	No	TSax	225	ad
280	L	*Eugongylus rufescens*	CT scan (Digimorph)	16	ex	Scincidae	No	LL	226	ad
2378	L	*Eulamprus quoyii*	**CT scan (ZMB 43336)**	**This work**	ex	Scincidae	Yes	TSax	226	ad
2361	L	*Eulamprus quoyii*	**CT scan (ZMB RE37)**	**This work**	ex	Scincidae	Yes	TSax	226	em(st10)
281	L	*Eumeces algeriensis*	CT scan (Digimorph)	16	ex	Scincidae	No	T	227	ad
282	L	*Eumeces schneideri*	CT scan (Digimorph)	Digimorph (unpublished)	ex	Scincidae	No	T	228	ad
292	L	*Hemiergis peronii*	C&S	90	ex	Scincidae	No	T/LL	229	em
2360	L	*Liopholis whitii*	**CT scan (ZMB 29584)**	**This work**	ex	Scincidae	No	TSax	230,231	ad
2122	L	*Liopholis whitii*	C&S	90	ex	Scincidae	No	TSax	230,231	em(st10)
2121	L	*Liopholis whitii*	C&S	90	ex	Scincidae	No	TSax	230,231	em
2127	L	*Mabuya sp.*	Acurate drawing	92	ex	Scincidae	No	T/LL	232	ad
2235	L	*Mochlus sundevalli*	**Picture (ZMB 37312)**	**This work**	ex	Scincidae	No	T	233,234	ad
2347	L	*Phoboscincus bocourti*	CT scan	93	ex	Scincidae	No	T	235	ad
2175	L	*Scincus scincus*	CT scan (Digimorph)	16	ex	Scincidae	No	F	236	ad
2182	L	*Sphenomorphus solomonis*	CT scan (Digimorph)	16	ex	Scincidae	No	LL	237	ad
2246	L	*Tiliqua scincoides*	CT scan (Digimorph)	16	ex	Scincidae	Yes	T	238	ad
606	L	*Tiliqua scincoides*	**CT scan (ZMB 17061)**	**This work**	ex	Scincidae	Yes	T	238	ad
2370	L	*Tiliqua nigrolutea*	**CT scan (ZMB HUB1)**	**This work**	ex	Scincidae	Yes	T	238	em(st10)
2189	L	*Trachylepis maculilabris*	Acurate drawing	92	ex	Scincidae	No	T	239	ad
2201	L	*Typhlosaurus braini*	Acurate drawing	88	ex	Scincidae	No	F	88	ad
2204	L	*Typhlosaurus vermis*	Acurate drawing	88	ex	Scincidae	No	F	88	ad
2177	L	*Shinisaurus crocodilurus*	CT scan (Digimorph)	94	ex	Shinisauridae	Yes	SAq	240	ad
2178	L	*Shinisaurus crocodilurus*	CT scan (Digimorph)	94	ex	Shinisauridae	No	SAq	240	ju
289	L	*Gonatodes albogularis*	CT scan (Digimorph)	16	ex	Sphaerodactylidae	No	Ar	241	ad
2342	L	*Teratoscincus przewalskii*	CT scan	74	ex	Sphaerodactylidae	No	T	242	ad
2180	O	*Sphenodon punctatus*	CT scan (Digimorph)	Digimorph (unpublished)	ex	Sphenodontidae	No	T	243	ad
2181	O	*Sphenodon punctatus*	CT scan (Digimorph)	Digimorph (unpublished)	ex	Sphenodontidae	No	T	243	ju
2376	O	*Sphenodon punctatus*	C&S	95	ex	Sphenodontidae	No	T	243	em(st10)
2375	O	*Sphenodon punctatus*	C&S	95	ex	Sphenodontidae	No	T	243	em
222	L	*Aspidoscelis tigris*	CT scan (Digimorph)	16	ex	Teiidae	No	T	244	ad
241	L	*Callopistes maculatus*	CT scan (Digimorph)	16	ex	Teiidae	No	T	245	ad
2365	L	*Kentropyx altamazonica*	**CT scan (ZMB 69836)**	**This work**	ex	Teiidae	Yes	SAr	246	ad
2366	L	*Kentropyx altamazonica*	**CT scan (AMNH 73471)**	**This work**	ex	Teiidae	Yes	SAr	246	em(st10)
2197	L	*Tupinambis teguixin*	CT scan (Digimorph)	16	ex	Teiidae	No	T	247	ad
2264	L	*Salvator merianae*	C&S	83	ex	Teiidae	No	T	248	em
2194	L	*Salvator merianae*	C&S	96	ex	Teiidae	No	T	248	em
2185	L	*Teius teyou*	CT scan (Digimorph)	16	ex	Teiidae	No	T	249	ad
270	L	*Diplometopon zarudnyi*	CT scan (Digimorph)	97	ex	Trogonophiidae	No	F	250	ad
2192	L	*Trogonophis wiegmanni*	CT scan (Digimorph)	16	ex	Trogonophiidae	No	F	250	ad
2152	L	*Plica plica*	CT scan (Digimorph)	16	ex	Tropiduridae	No	Ar	251	ad
2183	L	*Stenocercus guentheri*	CT scan (Digimorph)	16	ex	Tropiduridae	No	T	252	ad
2371	L	*Tropidurus torquatus*	**CT scan (LUOMUS 1195)**	**This work**	ex	Tropiduridae	Yes	SAr	253	ad

2263	L	*Tropidurus sp.*	C&S	83	ex	Tropiduridae	No	SAr	253	em
2262	L	*Tropidurus sp.*	C&S	83	ex	Tropiduridae	No	SAr	253	em
2207	L	*Uranoscodon superciliosus*	CT scan (Digimorph)	16	ex	Tropiduridae	Yes	SAr	254	ad
2219	L	*Varanus acanthurus*	CT scan (Digimorph)	16	ex	Varanidae	Yes	T/Ar	255	ad
2220	L	*Varanus exanthematicus*	CT scan (Digimorph)	16	ex	Varanidae	No	T	256	ad
2221	L	*Varanus gouldii*	CT scan (Digimorph)	Digimorph (unpublished)	ex	Varanidae	No	T	257	ad
5001	L	*Varanus panoptes*	CT scan	98	ex	Varanidae	No	T	190	em(st10)
5003	L	*Varanus panoptes*	CT scan	98	ex	Varanidae	No	T	190	em
5002	L	*Varanus panoptes*	CT scan	98	ex	Varanidae	No	T	190	em
2222	L	*Varanus salvator*	CT scan (Digimorph)	16	ex	Varanidae	No	SAq	258	ad
262	L	*Cricosaura typica*	CT scan (Digimorph)	Digimorph (unpublished)	ex	Xantusiidae	No	LL	259	ad
2110	L	*Lepidophyma flavimaculatum*	CT scan (Digimorph)	16	ex	Xantusiidae	No	TSax	260	ad
2111	L	*Lepidophyma gaigeae*	CT scan (Digimorph)	Digimorph (unpublished)	ex	Xantusiidae	No	TSax	190	ad
2112	L	*Lepidophyma smithii*	CT scan (Digimorph)	Digimorph (unpublished)	ex	Xantusiidae	No	TSax	260	ad
2250	L	*Xantusia bezyi*	CT scan (Digimorph)	Digimorph (unpublished)	ex	Xantusiidae	No	TSax	261	ad
2225	L	*Xantusia henshawi*	CT scan (Digimorph)	Digimorph (unpublished)	ex	Xantusiidae	No	TSax	262	ad
2254	L	*Xenosaurus grandis*	CT scan (Digimorph)	16	ex	Xenosauridae	Yes	TSax	263	ad

Supplementary Table 2 | List of identifiers and classifiers for all snake species used in the study (see legend in Supplementary Table 1).

ID	Group	Species	Source	Source (ref)	Type	Family	3D analysis	Ecology	Ecology (ref)	Stage
109	S	*Acrochordus granulatus*	**CT scan (ZMB 9444)**	**This work**	ex	Acrochordidae	Yes	Aq	264,265	ad
75	S	*Acrochordus granulatus*	**C&S (MCZ R1648600)**	**This work**	ex	Acrochordidae	No	Aq	264,265	em(st10)
1	S	*Acrochordus granulatus*	C&S	99	ex	Acrochordidae	No	Aq	264,265	em
5	S	*Anilius scytale*	CT scan (Digimorph)	16	ex	Aniliidae	No	F	264,266	ad
27	S	*Liotyphlops albirostris*	CT scan (Digimorph)	100	ex	Anomalepididae	Yes	F	267,268	ad
46	S	*Typhlophis squamosus*	CT scan (Digimorph)	100	ex	Anomalepididae	No	F	269	ad
81	S	*Anomalepis aspinosus*	Acurate drawing	15	ex	Anomalepididae	No	F	270	ad
65	S	*Anomochilus leonardi*	CT scan (Digimorph)	121	ex	Anomochilidae	Yes	F	271	ad
121	S	*Boa constrictor*	**CT scan (ZMB 56461)**	**This work**	ex	Boidae	Yes	SAr	264,269	ad
11	S	*Calabaria reinhardtii*	CT scan (Digimorph)	16	ex	Boidae	Yes	LL	264,272	ad
128	S	*Candoia superciliosa*	**CT scan (ZMB 9466)**	**This work**	ex	Boidae	Yes	T	264	ad
63	S	*Candoia carinata*	**C&S (MCZ R166747)**	**This work**	ex	Boidae	No	T	273	em(st10)
91	S	*Chilabothrus striatus*	Acurate drawing	15	ex	Boidae	No	T	274	ad
129	S	*Chilabothrus striatus*	CT scan (Digimorph)	16	ex	Boidae	Yes	T	274	ad
130	S	*Corallus hortulanus*	**CT scan (ZMB 63744)**	**This work**	ex	Boidae	Yes	Ar	264,275	ad
89	S	*Corallus ruschenbergerii*	Acurate drawing	15	ex	Boidae	No	Ar	275,276	ad
20	S	*Eryx colubrinus*	CT scan (Digimorph)	16	ex	Boidae	No	T	277	ad
139	S	*Eryx jaculus*	**CT scan (ZMB 24284)**	**This work**	ex	Boidae	Yes	T	278,279	ad
93	S	*Exiliboa placata*	Acurate drawing	15	ex	Boidae	No	T?	280	ad
88	S	*Lichanura trivirgata*	CT scan (Digimorph)	16	ex	Boidae	No	T	264,281	ad
26	S	*Lichanura trivirgata*	CT scan (Digimorph)	16	ex	Boidae	No	T	264,281	ju
48	S	*Ungaliophis continentalis*	CT scan (Digimorph)	16	ex	Boidae	Yes	SAr	264,282	ad
12	S	*Casarea dussumieri*	CT scan (Digimorph)	121	ex	Bolyeriidae	Yes	LL/SAr	264,282	ad
2	S	*Afronatrix anoscopus*	Ct scan	101	ex	Colubridae	No	T/SAq	283	ad
80	S	*Ahaetulla prasina*	Acurate drawing	15	ex	Colubridae	No	Ar	284	ad
4	S	*Amphiesma stolatum*	CT scan (Digimorph)	16	ex	Colubridae	No	LL	285	ad
112	S	*Arrhyton taeniatum*	**CT scan (ZMB 6600)**	**This work**	ex	Colubridae	Yes	T	264	ad
86	S	*Atractus erythromelas*	Acurate drawing	15	ex	Colubridae	No	T	286	ad
127	S	*Calamaria muelleri*	**Picture (ZMB 14999)**	**This work**	ex	Colubridae	No	LL	287	ad
14	S	*Coluber constrictor*	CT scan (Digimorph)	16	ex	Colubridae	No	T	288,289	ad
68	S	*Conophis lineatus*	Acurate drawing	102	ex	Colubridae	No	T	264,290	ad
131	S	*Coronella austriaca*	**CT scan (ZMB 33449)**	**This work**	ex	Colubridae	Yes	LL	291	ad
135	S	*Dasypeltis scabra*	**CT scan (ZMB 59037)**	**This work**	ex	Colubridae	Yes	Ar	292,293	ad
16	S	*Diadophis punctatus*	CT scan (Digimorph)	16	ex	Colubridae	No	T	289,294	ad
137	S	*Eirenis decemlineatus*	**CT scan (ZMB 11046)**	**This work**	ex	Colubridae	Yes	T	295,296	ad
138	S	*Eirenis rothii*	**CT scan (ZMB 77659)**	**This work**	ex	Colubridae	Yes	T	295,297	ad
173	S	*Farancia abacura*	Acurate drawing	103	ex	Colubridae	No	Aq	289,298	ad
174	S	*Pseudoeryx plicatilis*	Acurate drawing	103	ex	Colubridae	No	Aq	298	ad

175	S	*Helicops leopardinus*	C&S	104	ex	Colubridae	No	Aq	299	em
21	S	*Heterodon platirhinos*	CT scan (Digimorph)	16	ex	Colubridae	No	T	264	ad
140	S	*Heterodon platirhinos*	**CT scan (ZMB 13871)**	**This work**	ex	Colubridae	Yes	T	264	ad
94	S	*Lampropeltis getula*	Acurate drawing	15	ex	Colubridae	No	T	264,289	ad
23	S	*Lampropeltis getula*	CT scan (Digimorph)	Digimorph (unpublished)	ex	Colubridae	Yes	T	264,289	ad
144	S	*Lampropeltis getula*	CT scan (Digimorph)	Digimorph (unpublished)	ex	Colubridae	Yes	T	264,289	em(st10)
147	S	*Lycodon aulicus*	**CT scan (ZMB 1806)**	**This work**	ex	Colubridae	Yes	SAr	300,301	ad
33	S	*Natrix natrix*	CT scan (Digimorph)	16	ex	Colubridae	No	SAq	302	ad
152	S	*Natrix natrix*	**CT scan (ZMB 50818)**	**This work**	ex	Colubridae	Yes	SAq	302	ad
151	S	*Natrix natrix*	**CT scan (ZMB 28300)**	**This work**	ex	Colubridae	Yes	SAq	302	ad
150	S	*Natrix natrix*	**CT scan (ZMB 28224)**	**This work**	ex	Colubridae	Yes	SAq	302	ju
148	S	*Natrix natrix*	**CT scan (ZMB 18A)**	**This work**	ex	Colubridae	Yes	SAq	302	em
149	S	*Natrix natrix*	**CT scan (ZMB 18b)**	**This work**	ex	Colubridae	Yes	SAq	302	em
153	S	*Nerodia sipedon*	**CT scan (ZMB 37753)**	**This work**	ex	Colubridae	Yes	SAq	264,303	ad
154	S	*Opisthotropis latouchii*	**CT scan (ZMB 67308)**	**This work**	ex	Colubridae	Yes	SAq	304	ad
55	S	*Pantherophis guttatus*	Picture (Flicker)	Theil (unpublished)	ex	Colubridae	No	T	264,289	ad
34	S	*Pantherophis guttatus*	**CT scan (Lab)**	**This work**	ex	Colubridae	Yes	T	264,289	em(st10)
157	S	*Pantherophis obsoletus*	**CT scan (ZMB8402)**	**This work**	ex	Colubridae	Yes	SAr	264,305	ad
156	S	*Pantherophis obsoletus*	**CT scan (Lab)**	**This work**	ex	Colubridae	No	SAr	264,305	em(st10)
18	S	*Pantherophis obsoletus*	C&S	117	ex	Colubridae	No	SAr	264,305	em
17	S	*Pantherophis obsoletus*	C&S	117	ex	Colubridae	No	SAr	264,305	em
155	S	*Pantherophis obsoletus*	C&S	117	ex	Colubridae	No	SAr	264,305	em
99	S	*Phyllorhynchus decurtatus*	Acurate drawing	15	ex	Colubridae	No	T	289,306	ad
164	S	*Scaphiodontophis annulatus*	**CT scan (ZMB 64686)**	**This work**	ex	Colubridae	Yes	LL	264,307	ad
165	S	*Sibynophis collaris*	**CT scan (ZMB 28550)**	**This work**	ex	Colubridae	Yes	LL	300,308	ad
42	S	*Sonora semiannulata*	CT scan (Digimorph)	16	ex	Colubridae	No	LL	264,306	ad
43	S	*Thamnophis marcianus*	CT scan (Digimorph)	16	ex	Colubridae	No	SAq	309	ad
44	S	*Trimorphodon biscutatus*	CT scan (Digimorph)	16	ex	Colubridae	No	T	264,310	ad
51	S	*Xenochrophis piscator*	CT scan (Digimorph)	16	ex	Colubridae	No	T/SAq	300,311	ad
132	S	*Cylindrophis melanotus*	**CT scan (ZMB 14510)**	**This work**	ex	Cylindrophiidae	Yes	F	312	ad
15	S	*Cylindrophis ruffus*	CT scan (Digimorph)	16	ex	Cylindrophiidae	No	F	264	ad
133	S	*Cylindrophis ruffus*	**CT scan (MCZ R-172777)**	**This work**	ex	Cylindrophiidae	Yes	F	264	em(st10)
108	S	*Acanthophis antarcticus*	**CT scan (ZMB 38580)**	**This work**	ex	Elapidae	Yes	LL	313	ad
84	S	*Aspidelaps scutatus*	Acurate drawing	15	ex	Elapidae	No	T	314	ad
69	S	*Dendroaspis polylepis*	Acurate drawing	105	ex	Elapidae	No	SAr	315	ad
143	S	*Hydrophis gracilis*	**CT scan (ZMB 55909)**	**This work**	ex	Elapidae	Yes	Aq	316,317	ad
142	S	*Hydrophis gracilis*	**CT scan (AMNH 92707)**	**This work**	ex	Elapidae	Yes	Aq	316,317	em(st10)
172	S	*Hydrops martii*	Acurate drawing	106	ex	Elapidae	No	Aq	318	ad
24	S	*Laticauda colubrina*	CT scan (Digimorph)	16	ex	Elapidae	No	Aq	319,320	ad
30	S	*Micrurus fulvius*	CT scan (Digimorph)	16	ex	Elapidae	No	T	264	ad
32	S	*Naja naja*	CT scan (Digimorph)	16	ex	Elapidae	No	T	300,321	ad
31	S	*Naja kaouthia*	C&S	119	ex	Elapidae	No	T	300,321	em
97	S	*Notechis scutatus*	Acurate drawing	15	ex	Elapidae	No	T	322,323	ad
71	S	*Oxyuranus scutellatus*	Picture (EOL)	Matz (unpublished)	ex	Elapidae	No	T	323,324	ad
90	S	*Parahydrophis mertoni*	Acurate drawing	15	ex	Elapidae	No	M	320,325	ad
161	S	*Pseudechis porphyriacus*	**CT scan (ZMB 43283)**	**This work**	ex	Elapidae	Yes	T	326	ad
56	S	*Dinilysia patagonica*	Picture	107	fo	Fossil	No	?	34	ad
57	S	*Dinilysia patagonica*	Acurate drawing	34	fo	Fossil	No	?	34	ad
58	S	*Haasiophis terrasanctus*	Acurate drawing	43	fo	Fossil	No	M	43	ad

59	S	*Pachyrhachis problematicus*	Acurate drawing	40	fo	Fossil	No	M	40	ad
60	S	*Pachyrhachis problematicus*	3D reconstruction	42	fo	Fossil	No	M	42	ad
61	S	*Wonambi naracoortensis*	Acurate drawing	36	fo	Fossil	No	T	36	ad
62	S	*Yurlunggur camfieldensis*	Picture	37	fo	Fossil	No	T	37	ad
22	S	*Homalopsis buccata*	CT scan (Digimorph)	16	ex	Homalopsidae	No	SAq	327	ad
601	S	*Homalopsis buccata*	**CT scan (LUOMUS 1399)**	**This work**	ex	Homalopsidae	Yes	SAq	327	ad
110	S	*Amblyodipsas unicolor*	**CT scan (ZMB 77966)**	**This work**	ex	Lamprophiidae	Yes	F	328	ad
83	S	*Aparallactus modestus*	Acurate drawing	15	ex	Lamprophiidae	No	T	293	ad
600	S	*Aparallactus modestus*	**CT scan (ZMB 6910)**	**This work**	ex	Lamprophiidae	Yes	T	293	ad
113	S	*Atractaspis boulengeri*	**CT scan (ZMB 11040)**	**This work**	ex	Lamprophiidae	Yes	T	329	ad
120	S	*Boaedon fuliginosus*	**CT scan (ZMB 51392)**	**This work**	ex	Lamprophiidae	Yes	T	293	ad
119	S	*Boaedon fuliginosus*	**CT scan (Lab LF50)**	**This work**	ex	Lamprophiidae	Yes	T	293	em(st10)
118	S	*Boaedon fuliginosus*	**CT scan (Lab LF48)**	**This work**	ex	Lamprophiidae	Yes	T	293	em
117	S	*Boaedon fuliginosus*	**CT scan (Lab LF41)**	**This work**	ex	Lamprophiidae	Yes	T	293	em
116	S	*Boaedon fuliginosus*	**CT scan (Lab LF35)**	**This work**	ex	Lamprophiidae	Yes	T	293	em
136	S	*Duberria lutrix*	**CT scan (ZMB 1566)**	**This work**	ex	Lamprophiidae	Yes	T	330	ad
141	S	*Homoroselaps lacteus*	**CT scan (ZMB 80398)**	**This work**	ex	Lamprophiidae	No	T	331,332	ad
29	S	*Lycophidion capense*	CT scan (Digimorph)	16	ex	Lamprophiidae	No	T	331	ad
100	S	*Polemon collaris*	Acurate drawing	15	ex	Lamprophiidae	No	LL	293	ad
159	S	*Polemon gabonensis*	**CT scan (ZMB 21142)**	**This work**	ex	Lamprophiidae	Yes	LL	293	ad
160	S	*Prosymna ambigua*	**CT scan (ZMB 78750)**	**This work**	ex	Lamprophiidae	Yes	T	333	ad
168	S	*Psammophis sibilans*	**CT scan (ZMB 66045)**	**This work**	ex	Lamprophiidae	Yes	T	334	ad
35	S	*Psammophis sibilans*	C&S	108	ex	Lamprophiidae	No	T	334	em
163	S	*Pseudaspis cana*	**CT scan (ZMB 15255)**	**This work**	ex	Lamprophiidae	No	T	331	ad
95	S	*Epictia goudotii*	Acurate drawing	15	ex	Leptotyphlopidae	No	F	267	ad
25	S	*Leptotyphlops dulcis*	CT scan (Digimorph)	100	ex	Leptotyphlopidae	Yes	F	289	ad
107	S	*Myriopholis cairi*	Acurate drawing	15	ex	Leptotyphlopidae	No	F	335	ju
96	S	*Trilepida macrolepis*	Acurate drawing	15	ex	Leptotyphlopidae	No	F	267	ad
28	S	*Loxocemus bicolor*	CT scan (Digimorph)	16	ex	Loxocemidae	Yes	F	155,264	ad
111	S	*Aplopeltura boa*	**CT scan (ZMB 5397)**	**This work**	ex	Pareatidae	Yes	Ar	336	ad
158	S	*Pareas carinatus*	**CT scan (ZMB 20533)**	**This work**	ex	Pareatidae	Yes	Ar	264,336	ad
98	S	*Pareas margaritophorus*	Acurate drawing	15	ex	Pareatidae	No	Ar	336	ad
6	S	*Aspidites melanocephalus*	CT scan (Digimorph)	16	ex	Pythonidae	Yes	T	323	ad
70	S	*Liasis mackloti*	Acurate drawing	109	ex	Pythonidae	No	SAq	337	ad
64	S	*Liasis fuscus*	**CT scan (MCZ R166753)**	**This work**	ex	Pythonidae	No	SAq	323	em
126	S	*Malayopython reticulatus*	**CT scan (ZMB 45800)**	**This work**	ex	Phytonidae	Yes	T	338	ad
603	S	*Python bivittatus*	**CT scan (ZMB 30906)**	**This work**	ex	Pythonidae	Yes	T	338	ad
37	S	*Python molurus*	CT scan (Digimorph)	16	ex	Pythonidae	No	T	273	ad
101	S	*Python regius*	Acurate drawing	15	ex	Pythonidae	No	T	293	ju
38	S	*Python sebae*	Picture (Biolib)	Suber (unpublished)	ex	Pythonidae.	No	T	293	ad
39	S	*Python sebae*	C&S (picture)	110	ex	Pythonidae.	No	T	293	em(st10)
162	S	*Python sebae*	C&S (picture)	110	ex	Pythonidae	No	T	293	em
45	S	*Tropidophis haetianus*	CT scan (Digimorph)	16	ex	Tropidophiidae	Yes	LL	339	ad
78	S	*Acutotyphlops kunuaensis*	Acurate drawing	15	ex	Typhlopidae	No	F	340	ad
79	S	*Afrotyphlops punctatus*	Acurate drawing	15	ex	Typhlopidae	No	F	340	ad
102	S	*Ramphotyphlops lineatus*	Acurate drawing	15	ex	Typhlopidae	No	F	340	ad
74	S	*Ramphotyphlops braminus*	CT scan (Digimorph)	Digimorph (unpublished)	ex	Typhlopidae	Yes	F	340	ju
146	S	*Letheobia caeca*	**CT scan (ZMB 23294)**	**This work**	ex	Typhlopidae	Yes	F	334	ad
145	S	*Letheobia caeca*	**CT scan (AMNH 134214)**	**This work**	ex	Typhlopidae	Yes	F	334	em(st10)

72	S	*Ramphotyphlops sp.*	Acurate drawing	15	ex	Typhlopidae	No	F	340	ad
47	S	*Typhlops jamaicensis*	CT scan (Digimorph)	100	ex	Typhlopidae	No	F	340	ad
169	S	*Typhlops richardi*	**CT scan (ZMB 28590)**	**This work**	ex	Typhlopidae	Yes	F	340	ad
166	S	*Typhlops richardi*	**CT scan (AMNH 146788)**	**This work**	ex	Typhlopidae	Yes	F	340	em(st10)
10	S	*Brachyophidium rhodogaster*	Picture	17	ex	Uropeltidae	No	F	300	ad
36	S	*Pseudotyphlops philippinus*	Acurate drawing	111	ex	Uropeltidae	No	F	341	ad
40	S	*Rhinophis blythii*	Picture	17	ex	Uropeltidae	No	F	341	ad
41	S	*Rhinophis homolepis*	Picture	17	ex	Uropeltidae	No	F	341	ad
104	S	*Uropeltis ceylanicus*	Acurate drawing	15	ex	Uropeltidae	No	F	342	ad
105	S	*Uropeltis ocellata*	Acurate drawing	15	ex	Uropeltidae	No	F	343	ad
49	S	*Uropeltis rubromaculatus*	Picture	17	ex	Uropeltidae	No	F	344	ad
50	S	*Uropeltis woodmasoni*	CT scan (Digimorph)	17	ex	Uropeltidae	Yes	F	345	ad
3	S	*Agkistrodon contortrix*	CT scan (Digimorph)	16	ex	Viperidea	No	T	346	ad
85	S	*Atheris squamigera*	Acurate drawing	15	ex	Viperidae	No	Ar	347	ad
66	S	*Azemiops feae*	Acurate drawing	15	ex	Viperidae	No	T	347,348	ad
114	S	*Azemiops kharini*	**CT scan (ZMB 69985)**	**This work**	ex	Viperidae	Yes	T	347	ad
115	S	*Bitis arietans*	**CT scan (ZMB 16732)**	**This work**	ex	Viperidae	Yes	T	264,347	ad
87	S	*Bitis nasicornis*	Acurate drawing	15	ex	Viperidae	No	T	347	ad
9	S	*Bothrops asper*	CT scan (Digimorph)	16	ex	Viperidae	No	T	264,346	ad
53	S	*Bothrops jararaca*	CT scan/Acurate drawing	112	ex	Viperidae	No	T	346	ad
7	S	*Bothrops jararaca*	CT scan/Acurate drawing	112	ex	Viperidae	No	T	346	em(st10)
54	S	*Bothrops jararaca*	CT scan/Acurate drawing	112	ex	Viperidae	No	T	346	em
8	S	*Bothrops jararaca*	CT scan/Acurate drawing	112	ex	Viperidae	No	T	346	em
125	S	*Bothrops jararacussu*	**CT scan (ZMB 477490)**	**This work**	ex	Viperidae	Yes	T	346	ad
124	S	*Bothrops jararacussu*	**CT scan (ZMB 65523)**	**This work**	ex	Viperidae	Yes	T	346	em(st10)
123	S	*Bothrops jararacussu*	**CT scan (ZMB 65523)**	**This work**	ex	Viperidae	Yes	T	346	em
122	S	*Bothrops jararacussu*	**CT scan (ZMB 65523)**	**This work**	ex	Viperidae	Yes	T	346	em
13	S	*Causus rhombeatus*	CT scan (Digimorph)	16	ex	Viperidea	No	T	347	ad
167	S	*Daboia russelii*	**CT scan (ZMB 37714)**	**This work**	ex	Viperidae	Yes	T	347	ad
134	S	*Daboia russelii*	**CT scan (ZMB RE90)**	**This work**	ex	Viperidae	Yes	T	347	ju
92	S	*Eristicophis macmahoni*	Acurate drawing	15	ex	Viperidae	No	T	347	ad
103	S	*Sistrurus miliarius*	Acurate drawing	15	ex	Viperidae	No	T	346	ad
106	S	*Vipera latastei*	Acurate drawing	15	ex	Viperidae	No	T	347	ad
73	S	*Xenodermus javanicus*	Acurate drawing	109	ex	Xenodermatidae	No	Saq	349	ad
52	S	*Xenopeltis unicolor*	CT scan (Digimorph)	16	ex	Xenopeltidae	Yes	F	273	ad

Supplementary Table 3 | Definition of 2D landmark points (see also Supplementary Figure 2).

Landmark number	Bone/region	Definition
L1	Premaxilla	Rostral tip of the premaxilla. The premaxilla is reduced in some species, including chameleons and many caenophidian snakes, so other views have been used to place this landmark in those groups
L2	Premaxilla	Posterior tip of the transverse process of the premaxilla. This landmark is usually in contact with the maxillary bone in lizards, and the contact point can be reduced or lost in snakes. The premaxilla is reduced in some species, including chameleons and many caenophidian snakes, so other views have been used to place this landmark in those groups
L3	Maxilla	Anteriormost tip of the maxilla
L4	Nasal	Tip of the lateral process of the nasal bone. This process is less prominent in snakes, because of reduced nasal bones and loosening of their sutures
L5	Nasal	Tip of the premaxillary process of the nasal bone
L6	Prefrontal	Tip of the prefrontal lateral process in contact with the maxilla
L7	Prefrontal	Medial point of the postero-dorsal part of the prefrontal bone surrounding the optic region
L8	Maxilla	Posteriormost tip of the maxilla
L9	Parietal	Lateral edge of the fronto-parietal suture on the supraorbital process of the parietal bone
L10	Parietal	Most dorsal point of the parietal medial crest or tectum
L11	Parietal	Tip of the postero-dorsal process of the parietal bone (midline of the parietal). It can be bifurcated in lizards and snakes
L12	Parietal	Tip of the postparietal process (supratemporal process) of the parietal bone. The tip sticks out behind or above the quadrate bone or is located behind the supratemporal bone in lizards. In snakes, when present (lost in some fossorial groups), it is located rostrally or at the anterior part of the supratemporal bone
L13	Parietal	Inflection point on the ventro-lateral crest of parietal at the level of the prootic bone in lizards, and curvature of the posterior margin of parietal along the anterior parieto-prootic suture in snakes. The lateral wall downgrowth of parietal leads to an increased physical contact between parietal and prootic bones in both snakes and fossorial lizards. The homology of this landmark is ensured by the developmental characteristic of the trifurcated ossfication of parietal bone in lizards and snakes, and the topological position of the epipterygoid process and parietal downgrowth
L14	Parietal	Most ventral point of the descending lateral flanges of the parietal bone (downgrowth). In lizards, this landmark corresponds to the epipterygoid process of the parietal, which is located at the tip of the epipterygoid bone. The epipterygoid has been reduced (or lost) and replaced by the lateral wall of parietal in snakes and fossorial forms of lizards. During development of both snakes and lizards, the parietal start ossifying its lateral edges (as ossified lateral splints) before the tectum. Hence, the tip of the lateral downgrowth of parietal in snakes is homologous to the epipterygoid process of parietal in lizards
L15	Quadrate	Antero-ventral tip of the mandibular condyle of the quadrate bone (in lateral view)
L16	Quadrate	Inflection point on the antero-dorsal curvature of the cephalic condyle anterior margin of the quadrate bone (in lateral view)
L17	Quadrate	Inflection point on the postero-dorsal curvature of the cephalic condyle posterior margin of the quadrate bone (in lateral view)
L18	Quadrate	Inflection point on the posterior curvature of the central pillar of the quadrate bone between the cephalic and mandibular condyles (adjusted relative to landmarks L5 and L7). This forms the posterior edge of the quadrate conch in lizards (showing large variation in curvature and depth). Several alethinophidian snakes show a similar trend, especially the fossorial forms, but this curvature becomes gradually reduced in caenophidian snakes
L19	Quadrate	Postero-ventral tip of the mandibular condyle of the quadrate bone (in lateral view)
L20	Basioccipital	Most posterior point of the basioccipital bone. In snakes, it can be covered by the quadrate bone or the jaw in lateral view; in those cases, other views have been used to ensure correct placement of this landmark

Supplementary Table 4 | Definition of 3D landmark points (see also Supplementary Figure 2).

Landmark number	Bone/region	Definition
L1	Premaxilla	Tip of the nasal process of the premaxilla (or ascending process of the premaxilla)
L2	Premaxilla	Rostral tip of the premaxilla
L3	Premaxilla	Tip of the transverse process of the premaxilla along the external dorsal margin
L4	Premaxilla	Medial point of the incisive process (lizards) or vomerine process (snakes) of premaxilla
L5	Premaxilla	Inflection point on the curvature between the nasal and transverse processes of the premaxilla
L6	Maxilla	Dorsal tip along the rostral margin of the premaxillary process of maxilla
L7	Maxilla	Dorsal tip along the posterior margin of the ectopterygoid process of maxilla
L8	Maxilla	Ventral tip along the rostral margin of the premaxillary process of maxilla
L9	Maxilla	Ventral tip along the posterior margin of the ectopterygoid process of maxilla
L10	Premaxilla	Tip of the transverse process of the premaxilla along the medial margin
L11	Prefrontal	Medial tip of the frontal process of the prefrontal
L12	Prefrontal	Lateral tip of the frontal process of the prefrontal along the orbital margin
L13	Prefrontal	Tip of the prefrontal lateral foot process
L14	Prefrontal	Rostral tip of the prefrontal outer wall
L15	Nasal	Posterior tip of the medial margin of the nasal bone, usually located at the intersection point between the suture of the nasal and frontal bones. The contact between these bones has been increasingly reduced during snake evolution
L16	Nasal	Posterior tip of the lateral margin of nasal bone facing prefrontal and frontal bones, usually located at the intersection point between the suture of nasal, prefrontal, and frontal bones
L17	Nasal	Tip of the premaxillary process of the nasal bone on the lateral side (nearest tip at the end of the curvature along the rostral margin of nasal bone)
L18	Nasal	Tip of the premaxillary process of the nasal bone
L19	Nasal	Posteromedial tip of the medial process of the nasal bone
L20	Frontal	Medial point of intersection of the fronto-parietal suture on the frontal bone
L21	Frontal	Lateral tip of the fronto-parietal suture on the frontal bone
L22	Frontal	Anterior tip of the lateral margin of frontal bone facing nasal and prefrontal bones, usually located at the intersection point of the suture between nasal, prefrontal, and frontal bones
L23	Frontal	Tip of the fronto-nasal suture at the base of the olfactory process of the frontal bone. In lizards, these two bones are commonly separated by the nasal process of premaxilla
L24	Frontal	Most postero-ventral point of the crista cranii of the frontal bone facing the parietal bone
L25	Parietal	Medial tip of the groove between the parietal bifid supraoccipital processes. The groove between the processes is fused in some species, where it colocalizes with L26
L26	Parietal	Tip of the parietal bifid supraoccipital process in contact with the supraoccipital bone. In some lizard and snake species, this process is highly reduced but its base can still be identified, especially its medial position and the region of contact between parietal and supraoccipital bones
L27	Parietal	Tip of the postparietal process (supratemporal process) of the parietal bone
L28	Parietal	Lateral tip of the fronto-parietal suture on the parietal bone
L29	Parietal	Medial point of intersection of the fronto-parietal suture on the parietal bone
L30	Parietal	Most anterior point of the ventral edge of the parietal downgrowth
L31	Parietal	Most posterior point of the ventral edge of the parietal downgrowth
L32	Supraoccipital	Antero-lateral tip of the supraoccipital-prootic suture. When bones are fused, the limits of the supraoccipital can still be identified by a distinctive elevation of its margin

L33	Supraoccipital	Postero-lateral tip of the supraoccipital-paroccipital suture. When bones are fused, the limits of the supraoccipital can still be identified by a distinctive elevation of its margin
L34	Supraoccipital	Base of the processus ascendens of supraoccipital (lizards) or antero-medial tip of supraoccipital (snakes)
L35	Opisthotic	Ventral end of the opisthotic-prootic suture on the dorsal margin of the fenestra ovalis (identifiable even if bones are fused)
L36	Opisthotic	Inflection point on the posterior curvature of fenestra ovalis
L37	Opisthotic	Dorsal end of the opisthotic-prootic suture on the ventral margin of the fenestra ovalis (identifiable even if bones are fused)
L38	Opisthotic	Most ventral point along the opisthotic-prootic margin at the intersection or near the pterygoid suture
L39	Exoccipital	Most ventral point of the occipital condyle
L40	Exoccipital	Most dorsal point of the occipital condyle
L41	Exoccipital	Lateral end of the exoccipital margin facing the foramen magnum
L42	Exoccipital	Inflection point on the curvature of excoccipital bone along the border of the foramen magnum. It can be fused with the supraoccipital in fossorial forms and in some lizards, but the extremity can still be clearly identified
L43	Exoccipital	Intersection point between exoccipital, paraoccipital, and supraoccipital bones. These bones are often fused, but the extremities are still identifiable
L44	Exoccipital	Most posterior medial point of the basioccipital condyle
L45	Exoccipital	Most lateral point of the basioccipital condyle
L46	Exoccipital	Inflection point on the ottocipital side of the basioccipital condyle
L47	Exoccipital	Tip of the spheno-occipital tubercle
L48	Basioccipital	Antero-medial tip of the suture between basiooccipital and parabasisphenoid bones
L49	Parabasisphenoid	Tip of the sagittal crest
L50	Parabasisphenoid	Tip of the basipterygoid process. In snakes, this process is lost but its topological position is located at the level of the vidian canal opening
L51	Quadrate	Latero-ventral tip of the mandibular condyle of the quadrate
L52	Quadrate	Inflection point on the curvature of the mandibular articular surface of the quadrate
L53	Quadrate	Ventro-medial tip of the mandibular condyle of the quadrate
L54	Quadrate	Dorso-medial tip of the adductor crest of the quadrate or tympanic crest
L55	Quadrate	Dorsal tip of the quadrate pillar or middle of the quadrate blade in Caenophidian snakes where the pillar is less visible or lost
L56	Quadrate	Tip of the cephalic condyle of the quadrate, dorso-ventrally contiguous to the medial mandibular condyle
L57	Pterygoid	Most dorsal point of the suture between the pterygoid and palatine bones, on the pterygoid side
L58	Pterygoid	Most ventral point of the suture between the pterygoid and palatine bones, on the pterygoid side
L59	Pterygoid	Most dorsal point on the posterior tip of the quadrate process of pterygoid
L60	Pterygoid	Most ventral point on the posterior tip of the quadrate process of pterygoid
L61	Prefrontal	Most posterior point of the prefrontal medial foot process

Supplementary Table 5 | Convergence metrics and associated p-values for each ecology. Significant values for fossorial ecology are highlighted with bold font.

Main ecology	C1	p-value	C2	p-value	C3	p-value	C4	p-value
Aquatic	0.026	0.937	0.007	0.936	0.013	0.940	<0.001	0.504
Arboreal	0.031	0.964	0.008	0.979	0.015	0.953	<0.001	0.374
Fossorial	**0.094**	**<0.001**	**0.029**	**0.001**	**0.044**	**0.001**	**0.005**	**<0.001**
Leaf litter	0.0689	0.085	0.0208	0.130	0.032	0.102	0.002	0.014
Terrestrial	0.034	0.996	0.009	1.000	0.0156	0.996	<0.001	0.004

Supplementary Table 6 | Prediction of ancestral ecologies (for MRCAs of Toxicofera, crown snakes, and snakes and their sister group) from shape parameters using linear discriminant analysis (LDA).

Main ecology	MRCA Toxicofera	MRCA snakes and their sister group	MRCA crown snakes
Aquatic	0.032	0.058	0.011
Arboreal	0.307	0.137	0.010
Fossorial	<0.001	<0.001	**0.820**
Leaf litter	0.114	0.104	0.0070.054
Terrestrial	**0.547**	**0.700**	0.105

Supplementary Table 7 | List of centroid size and log-centroid size values (in mm) for all lizard, snake, and outgroup species used in the 2D analysis. Snake species are highlighted in grey. ID numbers are as in Supplementary Tables 1 and 2.

ID	Species	Family	Centroid Size	Log-Centroid Size
107	*Myriopholis cairi*	Leptotyphlopidae	4.042717219	1.396917045
74	*Indotyphlops braminus*	Typhlopidae	4.922809608	1.593879426
95	*Epictia goudotii*	Leptotyphlopidae	5.161971507	1.641318581
27	*Liotyphlops albirostris*	Anomalepididae	6.518526108	1.874648293
46	*Typhlophis squamosus*	Anomalepididae	6.520241545	1.874911422
169	*Typhlops richardi*	Typhlopidae	7.152085062	1.967403931
102	*Ramphotyphlops lineatus*	Typhlopidae	7.360355693	1.996108259
220	*Aprasia striolata*	Pygopodidae	7.529181412	2.018786326
146	*Letheobia caeca*	Typhlopidae	8.085468139	2.090068393
96	*Trilepida macrolepis*	Leptotyphlopidae	8.175145787	2.10109855
41	*Rhinophis homolepis*	Uropeltidae	8.264898067	2.112017398
2157	*Procellosaurinus tetradactylus*	Gymnophthalmidae	8.573899749	2.148722676
25	*Rena dulcis*	Leptotyphlopidae	8.90497586	2.186610206
2138	*Nothobachia ablephara*	Gymnophthalmidae	8.910367814	2.187215522
2204	*Typhlosaurus vermis*	Scincidae	8.925808828	2.188946949
2357	*Dibamus novaeguineae*	Dibamidae	9.143316831	2.213023211
2160	*Psilophthalmus paeminosus*	Gymnophthalmidae	9.174654033	2.216444686
244	*Calyptommatus sp.*	Gymnophthalmidae	9.844005253	2.286862666
2201	*Typhlosaurus braini*	Scincidae	9.899966809	2.292531405
65	*Anomochilus leonardi*	Anomochilidae	10.65572872	2.366097655
78	*Acutotyphlops kunuaensis*	Typhlopidae	11.54710005	2.446434328
2203	*Acontias lineatus*	Scincidae	11.57620551	2.448951743
2217	*Vanzosaura rubricauda*	Gymnophthalmidae	11.58536431	2.449742604
143	*Hydrophis gracilis*	Elapidae	11.89149136	2.475823133
10	*Brachyophidium rhodogaster*	Uropeltidae	11.98473992	2.483634167
138	*Eirenis rothii*	Colubridae	12.04635212	2.488761886
287	*Geocalamus acutus*	Amphisbaenidae	12.05113611	2.489158938
226	*Bipes biporus*	Bipedidae	12.387584	2.516694681
49	*Uropeltis rubromaculatus*	Uropeltidae	12.48583877	2.524595104
47	*Typhlops jamaicensis*	Typhlopidae	12.535832	2.528591103
262	*Cricosaura typica*	Xantusiidae	12.6156231	2.534935975
270	*Diplometopon zarudnyi*	Trogonophiidae	12.67733421	2.539815691
50	*Uropeltis woodmasoni*	Uropeltidae	12.97770699	2.563233039
131	*Coronella austriaca*	Colubridae	13.04347992	2.568288386
259	*Cordylosaurus subtessellatus*	Gerrhosauridae	13.08270402	2.571291054
2200	*Acontias aurantiacus*	Scincidae	13.25620193	2.584465513
223	*Bachia bicolor*	Gymnophthalmidae	13.37756539	2.59357908
219	*Amphisbaena kingii*	Amphisbaenidae	13.40842093	2.595882937
40	*Rhinophis blythii*	Uropeltidae	14.24831057	2.656638343
2243	*Amphisbaena darwinii*	Amphisbaenidae	14.43500711	2.669656306
217	*Anniella pulchra*	Anniellidae	14.48978726	2.673444074
100	*Polemon collaris*	Lamprophiidae	14.57285857	2.679160797
227	*Bipes canaliculatus*	Bipedidae	14.7803338	2.6932975

2202	*Acontias cregoi*	Scincidae	14.84292176	2.697523103
160	*Prosymna ambigua*	Lamprophiidae	15.32081548	2.729212393
267	*Delma borea*	Pygopodidae	15.40045498	2.734397053
127	*Calamaria muelleri*	Colubridae	16.20223424	2.785149149
79	*Afrotyphlops punctatus*	Typhlopidae	16.8985434	2.827227429
235	*Brachymeles gracilis*	Scincidae	17.48394749	2.861283174
2175	*Scincus scincus*	Scincidae	17.53321136	2.864096874
257	*Colobosaura modesta*	Gymnophthalmidae	17.65239376	2.870871398
42	*Sonora semiannulata*	Colubridae	17.73102992	2.875316207
2233	*Acontias meleagris*	Scincidae	17.76886005	2.87744749
2	*Afronatrix anoscopus*	Colubridae	18.1497816	2.898658527
136	*Duberria lutrix*	Lamprophiidae	18.41953075	2.913411555
2237	*Takydromus sexlineatus*	Lacertidae	18.78394626	2.933002583
2114	*Amphisbaena microcephalum*	Amphisbaenidae	19.09685645	2.949523738
2331	*Hemidactylus frenatus*	Gekkonidae	19.11625856	2.950539206
99	*Phyllorhynchus decurtatus*	Colubridae	19.12704502	2.951103303
2145	*Pholidobolus montium*	Gymnophthalmidae	19.13985348	2.951772731
86	*Atractus erythromelas*	Colubridae	19.24288057	2.957141152
16	*Diadophis punctatus*	Colubridae	19.5014095	2.970486745
289	*Gonatodes albogularis*	Sphaerodactylidae	19.61402724	2.976244986
2373	*Zootoca vivipara*	Lacertidae	19.65808911	2.978488913
141	*Homoroselaps lacteus*	Lamprophiidae	20.0353096	2.997496197
83	*Aparallactus modestus*	Lamprophiidae	20.04165891	2.997813053
2192	*Trogonophis wiegmanni*	Trogonophiidae	20.43166843	3.017086071
137	*Eirenis decemlineatus*	Colubridae	20.69864207	3.030068097
2250	*Xantusia bezyi*	Xantusiidae	21.09141993	3.048866319
2168	*Rhineura floridana*	Rhineuridae	21.11985205	3.050213454
2127	*Mabuya sp.*	Scincidae	21.23489553	3.055645844
2215	*Uta stansburiana*	Phrynosomatidae	21.24171081	3.05596674
139	*Eryx jaculus*	Boidae	21.31969002	3.05963106
154	*Opisthotropis latouchii*	Colubridae	21.51227038	3.068623488
2290	*Bunopus tuberculatus*	Gekkonidae	21.56552096	3.071095787
2225	*Xantusia henshawi*	Xantusiidae	21.67055872	3.075954598
90	*Parahydrophis mertoni*	Elapidae	21.83838766	3.083669323
158	*Pareas carinatus*	Pareatidae	21.94332304	3.088462904
93	*Exiliboa placata*	Boidae	22.6002151	3.117959424
2235	*Mochlus sundevalli*	Scincidae	22.66091737	3.120641739
2184	*Takydromus formosanus*	Lacertidae	22.70274673	3.122485918
110	*Amblyodipsas unicolor*	Lamprophiidae	22.7640872	3.125184171
164	*Scaphiodontophis annulatus*	Colubridae	22.79871826	3.126704318
68	*Conophis lineatus*	Colubridae	22.84725871	3.128831141
2189	*Trachylepis maculilabris*	Scincidae	22.95777878	3.133656824
256	*Coleonyx variegatus*	Eublepharidae	23.6600915	3.163789726
112	*Arrhyton taeniatum*	Colubridae	23.66361201	3.16393851
165	*Sibynophis collaris*	Colubridae	23.67237745	3.16430886
106	*Vipera latastei*	Viperidae	23.970496	3.176823741
135	*Dasypeltis scabra*	Colubridae	24.04241643	3.179819622
113	*Atractaspis boulengeri*	Lamprophiidae	24.04383783	3.17987874

2182	*Sphenomorphus solomonis*	Scincidae	24.04434353	3.179899773
85	*Atheris squamigera*	Viperidae	24.09317425	3.181928574
2137	*Potamites ecpleopus*	Gymnophthalmidae	24.35577777	3.192769102
2234	*Ichnotropis capensis*	Lacertidae	24.58266848	3.202041661
2356	*Chamaesaura anguina*	Cordylidae	24.82279871	3.211762534
2342	*Teratoscincus przewalskii*	Sphaerodactylidae	25.32930726	3.231962115
248	*Celestus enneagrammus*	Anguidea	25.46184622	3.237181105
98	*Pareas margaritophorus*	Pareatidae	26.08187342	3.261240568
132	*Cylindrophis melanotus*	Cylindrophiidae	26.49867668	3.277094795
104	*Uropeltis ceylanicus*	Uropeltidae	26.58284156	3.280265954
2129	*Moloch horridus*	Agamidae	26.81419899	3.28893156
29	*Lycophidion capense*	Lamprophiidae	26.86960609	3.290995763
2111	*Lepidophyma gaigeae*	Xantusiidae	26.92364187	3.293004781
2329	*Egernia depressa*	Scincidae	27.20939791	3.303562425
2244	*Phrynosoma platyrhinos*	Phrynosomatidae	27.56872957	3.316682144
2144	*Phelsuma lineata*	Gekkonidae	27.64143084	3.319315765
281	*Eumeces algeriensis*	Scincidae	27.94314989	3.330172085
147	*Lycodon aulicus*	Colubridae	28.08704883	3.335308574
2119	*Liolaemus bellii*	Liolaemidae	28.12085844	3.336511594
2247	*Tracheloptychus petersi*	Gerrhosauridae	28.23284592	3.340486049
214	*Gerrhosaurus skoogi*	Gerrhosauridae	28.29293907	3.342612271
111	*Aplopeltura boa*	Pareatidae	28.87249411	3.362889381
249	*Chalarodon madagascariensis*	Opluridae	28.98704558	3.366849026
329	*Amphiglossus splendidus*	Scincidae	29.1639033	3.372931756
213	*Amphisbaena fuliginosa*	Amphisbaenidae	29.49098012	3.384084458
2106	*Leiocephalus barahonensis*	Leiocephalidae	30.39030889	3.414123771
58	*Haasiophis terrasanctus*	Fossil	30.84967481	3.429126209
272	*Draco quinquefasciatus*	Agamidae	31.11754892	3.437771934
2371	*Tropidurus torquatus*	Tropiduridae	31.14898965	3.43878181
159	*Polemon gabonensis*	Lamprophiidae	31.38641461	3.446375144
108	*Acanthophis antarcticus*	Elapidae	31.44084503	3.448107845
12	*Casarea dussumieri*	Bolyeriidae	31.45005665	3.448400784
66	*Azemiops feae*	Viperidae	31.46357767	3.448830612
2205	*Uma scoparia*	Phrynosomatidae	31.84101706	3.460755303
45	*Tropidophis haetianus*	Tropidophiidae	31.87713808	3.461889078
2150	*Phymaturus palluma*	Liolaemidae	31.97950511	3.465095232
109	*Acrochordus granulatus*	Acrochordidae	32.11569773	3.469344936
4	*Amphiesma stolatum*	Colubridae	32.32221127	3.475754649
2112	*Lepidophyma smithii*	Xantusiidae	32.57297471	3.483482948
2360	*Liopholis whitii*	Scincidae	32.90342135	3.493576645
84	*Aspidelaps scutatus*	Elapidae	32.93736078	3.4946076
218	*Anolis carolinensis*	Dactyloidae	33.58042811	3.513943401
2174	*Sceloporus variabilis*	Phrynosomatidae	33.62340191	3.51522231
120	*Boaedon fuliginosus*	Lamprophiidae	33.66353921	3.516415329
20	*Eryx colubrinus*	Boidae	33.93920392	3.524570804
2318	*Agamura persica*	Gekkonidae	34.41301115	3.538434724
250	*Chalcides ocellatus*	Scincidae	34.84896603	3.551023468
2352	*Bradypodion pumilum*	Chamaeleonidae	34.88305702	3.552001239

2183	*Stenocercus guentheri*	Tropiduridae	34.94406027	3.553748505
36	*Pseudotyphlops philippinus*	Uropeltidae	35.40711688	3.566912842
2143	*Petrosaurus mearnsi*	Phrynosomatidae	35.48639626	3.569149419
2359	*Diploglossus lessonae*	Anguidae	36.02895876	3.584323025
2328	*Strophurus ciliaris*	Diplodactylidae	36.09995347	3.586291576
128	*Candoia superciliosa*	Boidae	36.76033428	3.604419391
2214	*Urostrophus vautieri*	Leiosauridae	37.1656044	3.615383721
88	*Lichanura trivirgata*	Boidae	37.63009403	3.627804103
103	*Sistrurus miliarius*	Viperidae	37.70038585	3.629670329
2310	*Temujinia ellisoni*	Fossil	38.14016566	3.641267944
153	*Nerodia sipedon*	Colubridae	38.21765839	3.64329767
299	*Lacerta viridis*	Lacertidae	38.37273246	3.647347115
70	*Liasis mackloti*	Pythonidae	38.52998025	3.651436646
140	*Heterodon platirhinos*	Colubridae	38.54140469	3.65173311
2341	*Tarentola americana*	Phyllodactylidae	38.81687566	3.658855092
30	*Micrurus fulvius*	Elapidae	39.0111948	3.663848651
69	*Dendroaspis polylepis*	Elapidae	39.15033918	3.667409086
2378	*Eulamprus quoyii*	Scincidae	39.43266669	3.674594577
13	*Causus rhombeatus*	Viperidea	39.66611078	3.68049719
2110	*Lepidophyma flavimaculatum*	Xantusiidae	40.17678436	3.693289325
130	*Corallus hortulanus*	Boidae	40.55027027	3.702542446
2379	*Psammodromus algirus*	Lacertidae	40.62498859	3.70438336
285	*Gambelia wislizenii*	Crotaphytidae	40.7331828	3.707043062
151	*Natrix natrix*	Colubridae	40.76345488	3.707785966
2103	*Lanthanotus borneensis*	Lanthanotidae	40.93182246	3.711907816
282	*Eumeces schneideri*	Scincidae	41.20388992	3.718532667
222	*Aspidoscelis tigris*	Teiidae	41.27664951	3.720296953
271	*Dipsosaurus dorsalis*	Iguanidae	41.46843138	3.724932448
3	*Agkistrodon contortrix*	Viperidea	41.9533692	3.736558744
92	*Eristicophis macmahoni*	Viperidae	41.95407588	3.736575589
2238	*Zonosaurus ornatus*	Gerrhosauridae	42.02862378	3.738350905
2303	*Priscagama gobiensis*	Fossil	42.47942991	3.749019957
2272	*Aciprion formosum*	Fossil	42.56040991	3.750924476
55	*Pantherophis guttatus*	Colubridae	42.59959879	3.751844835
168	*Psammophis sibilans*	Lamprophiidae	42.72938507	3.754886859
2362	*Gerrhonotus infernalis*	Anguidae	43.21185028	3.76611477
11	*Calabaria reinhardtii*	Boidae	43.22358503	3.766386296
73	*Xenodermus javanicus*	Xenodermatidae	43.56610927	3.774279538
161	*Pseudechis porphyriacus*	Elapidae	43.89437344	3.781786144
279	*Eublepharis macularius*	Eublepharidae	44.02926879	3.784854613
125	*Bothrops jararacussu*	Viperidae	44.0353777	3.784993349
326	*Agama agama*	Agamidae	44.46689982	3.794745088
2141	*Oplurus cyclurus*	Opluridae	44.58581051	3.797415658
280	*Eugongylus rufescens*	Scincidae	45.26957567	3.812635188
15	*Cylindrophis ruffus*	Cylindrophiidae	46.30722616	3.835298021
293	*Hemitheconyx caudicinctus*	Eublepharidae	47.11183195	3.852524179
2156	*Pristidactylus torquatus*	Leiosauridae	47.87061505	3.868501852
2109	*Leiosaurus catamarcensis*	Leiosauridae	48.12615491	3.87382579

51	*Xenochrophis piscator*	Colubridae	48.52924397	3.882166585
324	*Aeluroscalabotes felinus*	Eublepharidae	48.67156081	3.885094893
2108	*Leiolepis triploida*	Agamidae	48.73650055	3.886428247
14	*Coluber constrictor*	Colubridae	49.25681049	3.897047642
91	*Chilabothrus striatus*	Boidae	49.37997739	3.899545026
2254	*Xenosaurus grandis*	Xenosauridae	49.49724797	3.901917071
275	*Elgaria multicarinata*	Anguidea	49.50939263	3.902162402
2185	*Teius teyou*	Teiidae	49.55443351	3.903071732
43	*Thamnophis marcianus*	Colubridae	49.84945615	3.909007587
2116	*Lialis burtonis*	Pygopodidae	50.25104376	3.917031318
157	*Pantherophis obsoletus*	Colubridae	50.52596059	3.922487275
2313	*Zapsosaurus sceliphros*	Fossil	51.2109616	3.935953603
28	*Loxocemus bicolor*	Loxocemidae	51.35090245	3.938682511
2365	*Kentropyx altamazonica*	Teiidae	51.6203072	3.943915146
242	*Calotes emma*	Agamidae	51.76979816	3.946806932
44	*Trimorphodon biscutatus*	Colubridae	51.8774035	3.94888331
2155	*Polychrus marmoratus*	Polychrotidae	52.517299	3.96114262
2151	*Physignathus cocincinus*	Agamidae	52.63911007	3.963459381
2324	*Bronchocela jubata*	Agamidae	52.6685566	3.964018628
2207	*Uranoscodon superciliosus*	Tropiduridae	52.80940651	3.966689328
167	*Daboia russelii*	Viperidae	53.19484332	3.973961462
210	*Amphisbaena alba*	Amphisbaenidae	53.79630499	3.985204784
5	*Anilius scytale*	Aniliidae	53.91183612	3.987350048
2219	*Varanus acanthurus*	Varanidae	54.20170653	3.992712394
52	*Xenopeltis unicolor*	Xenopeltidae	54.49371709	3.998085412
263	*Crotaphytus collaris*	Crotaphytidae	54.56542392	3.999400421
23	*Lampropeltis getula*	Colubridae	55.30936936	4.012942322
241	*Callopistes maculatus*	Teiidae	55.67762304	4.019578325
2177	*Shinisaurus crocodilurus*	Shinisauridae	55.78559152	4.02151562
24	*Laticauda colubrina*	Elapidae	55.96825382	4.024784634
276	*Enyalioides laticeps*	Hoplocercidae	57.56048387	4.052836288
80	*Ahaetulla prasina*	Colubridae	58.38132122	4.066995997
174	*Pseudoeryx plicatilis*	Colubridae	59.32847187	4.083089324
234	*Brachylophus fasciatus*	Iguanidae	59.5169983	4.086261957
255	*Chamaeleo laevigatus*	Chamaeleonidae	59.97867747	4.093989123
2107	*Leiolepis belliana*	Agamidae	60.05281788	4.095224473
2167	*Rhacodactylus auriculatus*	Gekkonidae	61.12973896	4.112998474
121	*Boa constrictor*	Boidae	61.46496126	4.118467277
22	*Homalopsis buccata*	Homalopsidae	61.8046213	4.12397814
2152	*Plica plica*	Tropiduridae	62.37962162	4.133238645
173	*Farancia abacura*	Colubridae	62.51781695	4.135451587
260	*Smaug mossambicus*	Cordylidae	62.8080192	4.14008276
87	*Bitis nasicornis*	Viperidae	63.34711993	4.148629443
101	*Python regius*	Pythonidae	64.93741882	4.173424019
2245	*Saltuarius cornutus*	Diplodactylidae	66.51090769	4.19736596
48	*Ungaliophis continentalis*	Boidae	66.70800946	4.200325028
32	*Naja naja*	Elapidae	66.84263847	4.202341177
71	*Oxyuranus scutellatus*	Elapidae	69.47330091	4.240942519

2104	*Latastia longicaudata*	Lacertidae	69.63460489	4.243261641
163	*Pseudaspis cana*	Lamprophiidae	70.38265072	4.253946794
261	*Corytophanes cristatus*	Corytophanidae	73.3818659	4.295676846
2140	*Pseudopus apodus*	Anguidea	74.82056953	4.315092841
2249	*Saara hardwickii*	Agamidae	74.90681764	4.31624491
59	*Pachyrhachis problematicus*	Fossil	78.29453153	4.360477761
172	*Hydrops martii*	Elapidae	79.1490637	4.371332957
2283	*Eosaniwa koehni*	Fossil	79.42037353	4.374754929
2347	*Phoboscincus bocourti*	Scincidae	81.98786322	4.406571227
225	*Basiliscus basiliscus*	Corytophanidae	82.09441784	4.407870022
97	*Notechis scutatus*	Elapidae	82.83227889	4.416817827
89	*Corallus ruschenbergerii*	Boidae	84.33299192	4.434773152
288	*Broadleysaurus major*	Gerrhosauridae	84.86515941	4.441063637
2332	*Hypsilurus boydii*	Agamidae	85.79372313	4.451945847
296	*Hydrosaurus pustulatus*	Agamidae	88.06515147	4.478076898
2154	*Pogona vitticeps*	Agamidae	88.24270148	4.48009099
6	*Aspidites melanocephalus*	Pythonidae	89.87152053	4.498381101
291	*Heloderma suspectum*	Helodermatidae	91.90563032	4.520762293
115	*Bitis arietans*	Viperidae	94.50463594	4.548648891
2221	*Varanus gouldii*	Varanidae	95.09626028	4.554889645
2220	*Varanus exanthematicus*	Varanidae	98.47402083	4.589792765
2180	*Sphenodon punctatus*	Sphenodontidae	99.9007719	4.604177412
53	*Bothrops jararaca*	Viperidae	99.97932028	4.604963367
251	*Chamaeleo calyptratus*	Chamaeleonidae	101.2351204	4.617445736
9	*Bothrops asper*	Viperidae	101.7491856	4.622510821
290	*Heloderma horridum*	Helodermatidae	109.3080896	4.694170405
2246	*Tiliqua scincoides*	Scincidae	111.2689017	4.711949809
126	*Malayopython reticulatus*	Phytonidae	112.1977758	4.720263169
2197	*Tupinambis teguixin*	Teiidae	116.8030538	4.760489216
264	*Ctenosaura pectinata*	Iguanidae	118.2822063	4.773073348
37	*Python molurus*	Pythonidae	134.110865	4.898666809
38	*Python sebae*	Pythonidae	139.7387546	4.939774641
2364	*Iguana iguana*	Iguanidae	159.1266168	5.069700217
57	*Dinilysia patagonica*	Fossil	160.7608513	5.079917865
62	*Yurlunggur camfieldensis*	Fossil	193.2347434	5.263905737
61	*Wonambi naracoortensis*	Fossil	205.3190031	5.324564882
2222	*Varanus salvator*	Varanidae	218.8341312	5.388314051
2302	*Plotosaurus bennisoni*	Fossil	723.4586027	6.584043326
2295	*Mosasaurus hoffmanni*	Fossil	1953.282648	7.577266645

Supplementary Table 8 | List of centroid size and log-centroid size values (in mm) for all lizard, snake, and outgroup species used in the 3D analysis. Snake species are highlighted in grey. ID numbers are as in Supplementary Tables 1 and 2.

ID	Species	Family	Centroid Size	Log-Centroid Size
74	*Indotyphlops braminus*	Typhlopidae	9551.349558	9.164437739
27	*Liotyphlops albirostris*	Anomalepididae	11257.28968	9.328771169
25	*Rena dulcis*	Leptotyphlopidae	16074.59583	9.684995406
169	*Typhlops richardi*	Typhlopidae	16289.85948	9.698298075
146	*Letheobia caeca*	Typhlopidae	17539.81607	9.772228779
2357	*Dibamus novaeguineae*	Dibamidae	17757.32125	9.784553175
65	*Anomochilus leonardi*	Anomochilidae	19612.60076	9.883927534
160	*Prosymna ambigua*	Lamprophiidae	22432.38898	10.01826113
50	*Uropeltis woodmasoni*	Uropeltidae	23040.15549	10.04499386
138	*Eirenis rothii*	Colubridae	23256.5797	10.05434337
143	*Hydrophis gracilis*	Elapidae	24077.86277	10.08904814
217	*Anniella pulchra*	Anniellidae	24821.56684	10.11946818
131	*Coronella austriaca*	Colubridae	24886.32728	10.12207383
136	*Duberria lutrix*	Lamprophiidae	31237.70624	10.34938118
600	*Aparallactus modestus*	Lamprophiidae	33623.83861	10.42299058
2373	*Zootoca vivipara*	Lacertidae	34239.51599	10.44113569
2215	*Uta stansburiana*	Phrynosomatidae	36890.75511	10.51571626
137	*Eirenis decemlineatus*	Colubridae	38492.11459	10.55820868
159	*Polemon gabonensis*	Lamprophiidae	38545.12897	10.55958501
112	*Arrhyton taeniatum*	Colubridae	39562.44847	10.58563568
154	*Opisthotropis latouchii*	Colubridae	39623.08006	10.58716706
110	*Amblyodipsas unicolor*	Lamprophiidae	39778.38556	10.59107897
139	*Eryx jaculus*	Boidae	39932.31318	10.59494113
135	*Dasypeltis scabra*	Colubridae	40031.04245	10.59741049
609	*Brookesia brygooi*	Chameleonidae	41217.60895	10.62662085
109	*Acrochordus granulatus*	Acrochordidae	42132.92488	10.64858478
608	*Anolis sagrei*	Dactyloidae	42689.39029	10.6617057
113	*Atractaspis boulengeri*	Lamprophiidae	43698.67906	10.68507315
164	*Scaphiodontophis annulatus*	Colubridae	43724.54974	10.685665
158	*Pareas carinatus*	Pareatidae	43894.36207	10.68954116
165	*Sibynophis collaris*	Colubridae	45007.23072	10.71457844
248	*Celestus enneagrammus*	Anguidea	45071.78018	10.71601161
12	*Casarea dussumieri*	Bolyeriidae	46826.29742	10.75420023
111	*Aplopeltura boa*	Pareatidae	47025.86335	10.75845301
249	*Chalarodon madagascariensis*	Opluridae	48713.29743	10.79370732
45	*Tropidophis haetianus*	Tropidophiidae	48772.53169	10.79492256
147	*Lycodon aulicus*	Colubridae	49157.97947	10.80279446
2106	*Leiocephalus barahonensis*	Leiocephalidae	50045.45031	10.82068688
2205	*Uma scoparia*	Phrynosomatidae	52330.64381	10.8653374
114	*Azemiops kharini*	Viperidae	52431.58513	10.86726446
48	*Ungaliophis continentalis*	Boidae	53386.62222	10.88531547
601	*Homalopsis buccata*	Homalopsidae	53498.18649	10.88740303
132	*Cylindrophis melanotus*	Cylindrophiidae	53668.74242	10.89058603

2371	*Tropidurus torquatus*	Tropiduridae	53788.92569	10.89282288
611	*Draco volans*	Agamidae	55554.12694	10.92511308
2150	*Phymaturus palluma*	Liolaemidae	55918.24788	10.93164604
605	*Tarentola mauritanica*	Phyllodactylidae	56633.32772	10.94435292
128	*Candoia superciliosa*	Boidae	59208.51989	10.98882073
250	*Chalcides ocellatus*	Scincidae	62285.2042	11.03947918
607	*Agama hispida*	Agamidae	62432.52764	11.04184169
108	*Acanthophis antarcticus*	Elapidae	62492.96196	11.04280922
140	*Heterodon platirhinos*	Colubridae	63013.217	11.05109978
2365	*Kentropyx altamazonica*	Teiidae	63532.47667	11.0593065
2103	*Lanthanotus borneensis*	Lanthanotidae	65537.94238	11.09038453
120	*Boaedon fuliginosus*	Lamprophiidae	65946.97516	11.09660629
285	*Gambelia wislizenii*	Crotaphytidae	66098.46832	11.09890085
2378	*Eulamprus quoyii*	Scincidae	67431.99919	11.11887495
11	*Calabaria reinhardtii*	Boidae	69160.63714	11.14418715
130	*Corallus hortulanus*	Boidae	69864.25539	11.15430943
610	*Bronchocela jubata*	Agamidae	70174.78485	11.15874434
151	*Natrix natrix*	Colubridae	73522.01902	11.20534022
125	*Bothrops jararacussu*	Viperidae	74155.40682	11.21391826
28	*Loxocemus bicolor*	Loxocemidae	74417.58527	11.21744755
2362	*Gerrhonotus infernalis*	Anguidae	74676.02482	11.22091437
168	*Psammophis sibilans*	Lamprophiidae	74700.22937	11.22123844
161	*Pseudechis porphyriacus*	Elapidae	75704.78851	11.23459669
2156	*Pristidactylus torquatus*	Leiosauridae	77860.54261	11.26267459
153	*Nerodia sipedon*	Colubridae	78751.49054	11.27405248
2207	*Uranoscodon superciliosus*	Tropiduridae	83006.87669	11.32667874
52	*Xenopeltis unicolor*	Xenopeltidae	85189.57272	11.35263432
2254	*Xenosaurus grandis*	Xenosauridae	85625.87789	11.35774283
263	*Crotaphytus collaris*	Crotaphytidae	86085.00793	11.36309055
2219	*Varanus acanthurus*	Varanidae	87373.3877	11.37794603
604	*Pogona barbata*	Agamidae	89639.03812	11.4035462
167	*Daboia russelii*	Viperidae	90083.91889	11.40849695
606	*Tiliqua scincoides*	Scincidae	91337.39322	11.42231555
157	*Pantherophis obsoletus*	Colubridae	91469.71091	11.42376317
23	*Lampropeltis getula*	Colubridae	92986.43672	11.44020892
2177	*Shinisaurus crocodilurus*	Shinisauridae	94642.08746	11.45785756
121	*Boa constrictor*	Boidae	96404.05245	11.47630352
234	*Brachylophus fasciatus*	Iguanidae	98701.10458	11.49985142
276	*Enyalioides laticeps*	Hoplocercidae	100289.8867	11.51582014
115	*Bitis arietans*	Viperidae	116517.4568	11.66579638
225	*Basiliscus basiliscus*	Corytophanidae	122608.8829	11.71675475
2249	*Saara hardwickii*	Agamidae	124348.729	11.73084523
6	*Aspidites melanocephalus*	Pythonidae	142763.1384	11.86894216
291	*Heloderma suspectum*	Helodermatidae	159630.2429	11.98061544
603	*Python bivittatus*	Pythonidae	192889.284	12.16987165
126	*Malayopython reticulatus*	Phytonidae	202721.0409	12.21958613

Supplementary Table 9 | Allometric test. The percentage of shape predicted by size in both 2D and 3D analyses (using lizards and snakes together or separately) is highlighted in bold.

	Total Sum of Squares (SS)	Predicted SS	Residual SS	**% predicted**
Lizards and snakes (2D)	2.69	0.16	2.53	**6.06**
Lizards and snakes (3D)	8.26	1.23	7.02	**14.94**
Lizards (2D)	1.38	0.07	1.32	**4.76**
Snakes (2D)	1.34	0.11	1.23	**8.40**

Supplementary Table 10 | Phenotypic analysis of ontogenetic trajectories between two points (stage 10 embryo and adult, see Supplementary Figure 8) in snake (S) versus lizard (L) species. The analysis assesses for differences in path length and angle of trajectories.

1) Ontogenetic lengths

*Observed lengths:

L	S
0.109	0.111

*Pairwise absolute differences between lengths:

	L	S
L	0.000	0.002
S	0.002	0.000

*Size effect:

	L	S
L	0.000	0.066
S	0.066	0.0000

*p-values:

	L	S
L	1.00	**0.94**
S	**0.94**	1.000

2) Ontogenetic angles

*Pairwise angles (in degrees):

	L	S
L	0.000	43.683
S	43.683	0.000

*Size effect:

	L	S
L	0.000	0.859
S	0.859	0.000

*p-values:

	L	S
L	1.00	**0.65**
S	**0.65**	1.00

Supplementary Table 11 | Angles, lengths, and slopes of pooled snake and lizard ontogenetic trajectories (between stage 10 embryo and adult, see Supplementary Figure 10) from the regression of shape on log-centroid size.

Regression analysis							
		**Slope**		**Length**		**Angle**	
	n	Mean	St Dev	Mean	St Dev	Mean	St Dev
Lizards	16	0.06	0.05	0.74	0.35	3.24	2.56
Snakes	13	**0.19****	0.07	0.92	0.41	**10.58****	3.65

** p-value (t-test) < 0.01

Supplementary Table 12 | List of post-oviposition incubation periods for oviparous lizard (L) and snake (S) species (only one representative species per genus) at similar temperature (30 +/- 1^{0}C). Snake species are highlighted in grey.

Group	Species	Temperature (°C)*	Duration (days)*	Family
L	*Acanthocercus atricollis*	30	75	Agamidae
L	*Agama impalearis*	30	54	Agamidae
L	*Calotes versicolor*	30	37	Agamidae
L	*Chlamydosaurus kingii*	29.5	62.5	Agamidae
L	*Ctenophorus decresii*	30.5	59	Agamidae
L	*Draco spilopterus*	29	36	Agamidae
L	*Hydrosaurus amboinensis*	30	78.5	Agamidae
L	*Paralaudakia caucasia*	30	72	Agamidae
L	*Leiolepis guttata*	30	64	Agamidae
L	*Phrynocephalus mystaceus*	30	61	Agamidae
L	*Physignathus cocincinus*	30	101	Agamidae
L	*Pogona vitticeps*	30	60	Agamidae
L	*Trapelus mutabilis*	30	61	Agamidae
L	*Tympanocryptis tetraporophora*	30	48	Agamidae
L	*Uromastyx acanthinura*	30	99.5	Agamidae
L	*Pseudopus apodus*	30	52	Anguidae
L	*Chamaeleo africanus*	30	183	Chamaeleonidae
L	*Furcifer antimena*	30	360	Chamaeleonidae
L	*Basiliscus basiliscus*	30	98	Corytophanidae
L	*Laemanctus longipes*	30	64	Corytophanidae
L	*Crotaphytus collaris*	30	86	Crotaphytidae
L	*Gambelia wislizenii*	30	60	Crotaphytidae
L	*Anolis bimaculatus*	30	43	Dactyloidae
L	*Coleonyx brevis*	30	70	Eublepharidae
L	*Hemidactylus mabouia*	30	60	Eublepharidae
L	*Chondrodactylus angulifer*	30	85	Gekkonidae
L	*Geckolepis typica*	30	45	Gekkonidae
L	*Hemidactylus brookii*	30	55	Gekkonidae
L	*Homopholis mulleri*	30	80	Gekkonidae
L	*Lepidodactylus lugubris*	30	100	Gekkonidae
L	*Lygodactylus pictus*	30	78	Gekkonidae
L	*Pachydactylus tsodiloensis*	30	49	Gekkonidae
L	*Phelsuma borbonica*	30	64	Gekkonidae
L	*Stenodactylus sthenodactylus*	30	80	Gekkonidae
L	*Uroplatus phantasticus*	30	61	Gekkonidae
L	*Broadleysaurus major*	30	77	Gerrhosauridae
L	*Neusticurus bicarinatus*	30	86	Gymnophthalmidae
L	*Heloderma horridum*	30	170	Helodermatidae
L	*Conolophus subcristatus*	30	105	Iguanidae
L	*Ctenosaura bakeri*	29.5	90.5	Iguanidae
L	*Cyclura collei*	29.5	85	Iguanidae
L	*Iguana delicatissima*	30	119.5	Iguanidae
L	*Sauromalus ater*	29.5	83.5	Iguanidae
L	*Gallotia galloti*	30	90	Lacertidae
L	*Lacerta agilis*	29.5	34	Lacertidae

L	*Darevskia armeniaca*	30	55	Lacertidae
L	*Archaeolacerta bedriagae*	30	40	Lacertidae
L	*Dinarolacerta mosorensis*	30	19	Lacertidae
L	*Dalmatolacerta oxycephala*	30	49	Lacertidae
L	*Parvilacerta parva*	30	33	Lacertidae
L	*Omanosaura jayakari*	30	94	Lacertidae
L	*Podarcis muralis*	30	27	Lacertidae
L	*Teira dugesii*	30.5	51	Lacertidae
L	*Timon lepidus*	30	88	Lacertidae
L	*Petrosaurus thalassinus*	29.5	56	Phrynosomatidae
L	*Phrynosoma asio*	30	80.5	Phrynosomatidae
L	*Sceloporus scalaris*	30	44	Phrynosomatidae
L	*Uta stansburiana*	30	47	Phrynosomatidae
L	*Asaccus platyrhynchus*	30	48	Phyllodactylidae
L	*Ptyodactylus hasselquistii*	30	85.5	Phyllodactylidae
L	*Tarentola mauritanica*	30	90	Phyllodactylidae
L	*Polychrus marmoratus*	30	133	Polychrotidae
L	*Ctenotus taeniolatus*	30	40	Scincidae
L	*Bassiana duperreyi*	30	29	Scincidae
L	*Eumeces algeriensis*	30	47	Scincidae
L	*Lampropholis guichenoti*	30	27.5	Scincidae
L	*Morethia adelaidensis*	30	29	Scincidae
L	*Scincus scincus*	29.4	64	Scincidae
L	*Gonatodes albogularis*	30	72	Sphaerodactylidae
L	*Saurodactylus mauritanicus*	30	60	Sphaerodactylidae
L	*Teratoscincus microlepis*	30	75	Sphaerodactylidae
L	*Tupinambis teguixin*	30	171	Teiidae
L	*Varanus bengalensis*	30	170	Varanidae
S	*Calabaria reinhardtii*	30.5	37.5	Calabariidae
S	*Boiga dendrophila*	30	92	Colubridae
S	*Hemorrhois hippocrepis*	30	66	Colubridae
S	*Coniophanes fissidens*	30	53	Colubridae
S	*Coronella girondica*	30	51	Colubridae
S	*Dasypeltis scabra*	30	90	Colubridae
S	*Dipsas articulata*	30	85	Colubridae
S	*Orthriophis cantoris*	30	102	Colubridae
S	*Pantherophis guttatus*	30	60	Colubridae
S	*Farancia abacura*	30	57	Colubridae
S	*Gonyosoma oxycephalum*	30	115	Colubridae
S	*Heterodon nasicus*	30	59	Colubridae
S	*Lampropeltis getula*	30	75	Colubridae
S	*Erythrolamprus poecilogyrus*	30	93	Colubridae
S	*Liopholidophis dolicocercus*	30	59	Colubridae
S	*Coluber flagellum*	30	79	Colubridae
S	*Natrix matrix*	30	63	Colubridae
S	*Philodryas patagoniensis*	30	59	Colubridae
S	*Pituophis lineaticollis*	30	68	Colubridae
S	*Ptyas mucosa*	30	60	Colubridae
S	*Rhabdophis tigrinus*	30	47	Colubridae
S	*Rhinocheilus lecontei*	30	68	Colubridae

S	*Sonora semiannulata*	30	56	Colubridae
S	*Spalerosophis diadema*	30	84	Colubridae
S	*Spilotes pullatus*	30	56	Colubridae
S	*Telescopus fallax*	30	71	Colubridae
S	*Thrasops jacksonii*	30	84.5	Colubridae
S	*Aspidelaps scutatus*	30	67	Elapidae
S	*Bungarus caeruleus*	29.5	60	Elapidae
S	*Cacophis squamulosus*	30	74	Elapidae
S	*Demansia vestigiata*	30	67	Elapidae
S	*Dendroaspis angusticeps*	30	107	Elapidae
S	*Naja haje*	30	77	Elapidae
S	*Oxyuranus scutellatus*	30	71.5	Elapidae
S	*Pseudechis australis*	30	68	Elapidae
S	*Pseudonaja modesta*	30	61	Elapidae
S	*Vermicella intermedia*	30	59	Elapidae
S	*Boaedon fuliginosus*	30	60	Lamprophiidae
S	*Malpolon monspessulanus*	30	60	Lamprophiidae
S	*Xenocalamus transvaalensis*	30	55	Lamprophiidae
S	*Pareas carinatus*	30	62	Pareatidae
S	*Aspidites melanocephalus*	30	58	Pythonidae
S	*Bothrochilus albertisii*	30	68	Pythonidae
S	*Leiopython albertisii*	30	60	Pythonidae
S	*Simalia boeleni*	30	71	Pythonidae
S	*Liasis fuscus*	30	52	Pythonidae
S	*Morelia amethistina*	30.5	85	Pythonidae
S	*Python curtus*	30.5	65.5	Pythonidae
S	*Malayopython reticulatus*	30	87.5	Pythonidae
S	*Anilios australis*	29.5	58	Typhlopidae
S	*Cerastes cerastes*	30	48	Viperidae
S	*Lachesis muta*	30	61	Viperidae
S	*Macrovipera lebetina*	30	42	Viperidae
S	*Pseudocerastes fieldi*	30	17	Viperidae
S	*Xenopeltis unicolor*	30	76	Xenopeltidae

* mean value of temperature and/or number of days

Supplementary Table 13 | Analysis of variance (ANOVA) of the embryonic period (incubation period, see Supplementary Table 12) in lizards and snakes.

Duration of development					
	df	Sum Square	Mean Square	F-value	P(>F)
Group	1	2160	2160.4	1.51	**0.22**
Residuals	126	179990	1428.5		

Supplementary Table 14 | Discrete character coding of the ossification levels of both frontal and parietal bones in stage 10 snake and lizard embryos. Increasing score number (0-3) and grayscale intensity reflect more advanced ossification level (see legend and 3D rendered skull examples of analyzed species with their associated skull bone ossification color codes). New specimens produced by this work are highlighted in bold.

Group	Family	Last stage of development**	Ossification of frontal*	Ossification of parietal*	Reference
Outgroup	Sphenodontidae	*Sphenodon punctatus*	3	2	Howes and Swiknertok 1901[95]
**Lizard**	**Agamidae**	***Pogona vitticeps***	0	0	**This work**
**Lizard**	**Anguidae**	***Anguis fragilis***	1	0	**This work**
**Lizard**	**Anguidae**	***Celestus costatus***	2	2	**This work**
Lizard	Chamaleonidae	*Chamaeleo hoehnelii*	1	0	Rieppel 1993[67]
Lizard	Gymnophthalmidae	*Nothobachia ablephara*	3	0	Roscito & Rodrigues 2012[77]
Lizard	Gymnophthalmidae	*Calyptommatus sinebrachiatus*	1	0	Roscito & Rodrigues 2012[77]
Lizard	Gymnophthalmidae	*Vanzosaura rubricauda*	1	0	Roscito 2010[78]
Lizard	Iguanidae	Iguana iguana	0	0	Lima 2015[113]
Lizard	Leiosauridae	*Anisolepis longicauda*	0	0	Guerra-Fuentes 2006[83]
**Lizard**	**Leiosauridae**	***Urostrophus vautieri***	3	2	**This work**
Lizard	Liolaemidae	*Liolaemus scapularis*	2	1	Lobo et al. 1995[85]
**Lizard**	**Phyllodactylidae**	***Tarentola mauritanica***	1	0	**This work**
Lizard	Scincidae	*Liopholis whitii*	1	0	Hugi et al. 2010[91]
**Lizard**	**Scincidae**	***Eulamprus quoyii***	1	0	**This work**
Lizard	Scincidae	*Acontias meleagris*	3	2	Brock 1941[114]
**Lizard**	**Scincidae**	***Chalcides chalcides***	2	1	**This work**
**Lizard**	**Scincidae**	***Tiliqua nigrolutea***	3	2	**This work**
**Lizard**	**Teiidae**	***Kentropyx altamazonica***	0	1	**This work**
Lizard	Tropiduridae	*Tropidurus sp*	0	0	Guerra-Fuentes 2006[83]
Lizard	Varanidae	*Varanus panoptes*	3	3	Werneburg et al. 2015[98]
Snake	Achrocordidae	*Acrochordus granulatus*	3	3	Rieppel & Zaher 2001[99]
**Snake**	**Boidae**	***Candoia carinata***	3	3	**This work**
**Snake**	**Cylindrophiidae**	***Cylindrophis ruffus***	3	3	**This work**
Snake	Colubridae	*Lampropeltis getula*	2	2	Digimorph
Snake	Colubridae	*Crotaphopeltis hotamboia*	2	2	Brock 1929[115]
Snake	Colubridae	*Pantherophis obsoletus*	3	3	**This work**
**Snake**	**Colubridae**	***Pantherophis guttatus***	3	3	**This work**
**Snake**	**Elapidae**	***Hydrophis gracilis***	3	3	**This work**
Snake	Elapidae	*Naja h. haje*	3	2/3	Khannoon & Evans 2015[116]
**Snake**	**Lamprophiidae**	***Boaedon fuliginosus***	**3**	2	**This work**
Snake	Pythonidae	*Python sebae*	3	3	Boughner et al. 2007[110]
**Snake**	**Typhlopidae**	***Letheobia caeca***	0	0	**This work**
**Snake**	**Typhlopidae**	***Typhlops richardi***	0	0	**This work**
Snake	Viperidae	*Bothropoides jararaca*	3	3	Polachowski & Werneburg 2013[112]

*Levels of ossification

0 Poorly ossified

Frontal: anterior and posterior regions are largely unossified

Parietal: a large fontanelle, only the lateral wall or splints are ossified

1 Partially ossified

Frontal: anterior region is ossified and posterior central region remains unossified and not fused (in species where bones fuse)

Parietal: still has a large fontanelle but the posterior region has ossified and fused in the midline

2 Mostly ossified

Frontal: posterior region is largely ossified and fused (in species where bones fuse)

Parietal: a small fontanelle remains unossified on the parietal roof

3 Completely ossified

Frontal: fully ossified

Parietal: fully ossified

**Late embryos (stage 10)

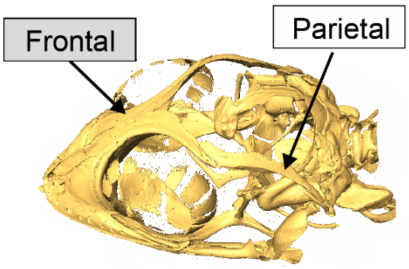

Tarentola mauritanica (lizard)

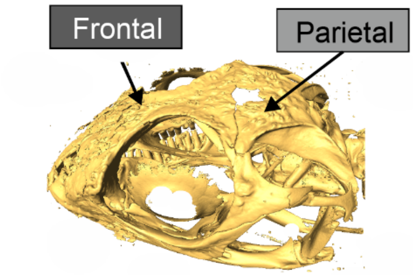

Urosthrophus vautieri (lizard)

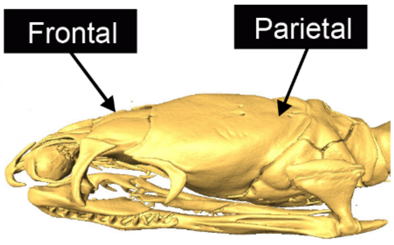

Hydrophis gracilis (snake)

Supplementary References

1. Pyron, R.A., Burbrink, F.T. & Wiens, J.J. A phylogeny and revised classification of Squamata, including 4161 species of lizards and snakes. *BMC Evol. Biol.* **13,** 93 (2013).
2. Hsiang, A.Y et al. The origin of snakes: revealing the ecology, behavior, and evolutionary history of early snakes using genomics, phenomics, and the fossil record. *BMC Evol. Biol.* **15,** 87 (2015).
3. Reeder, T.W. et al. Integrated Analyses Resolve Conflicts over Squamate Reptile Phylogeny and Reveal Unexpected Placements for Fossil Taxa. *PLoS One* **10,** e0118199 (2015).
4. Pyron, R.A. Novel Approaches for Phylogenetic Inference from Morphological Data and Total-Evidence Dating in Squamate Reptiles (Lizards, Snakes, and Amphisbaenians). *Syst. Biol.* **66,** 38-56 (2016).
5. Tonini, J.F.R., Beard, K.H., Ferreira, R.B., Jetz, W. & Pyron, R.A. Fully-sampled phylogenies of squamates reveal evolutionary patterns in threat status. *Biol. Cons.* **204,** 23-31 (2016).
6. Bhullar, B.A. et al. Birds have paedomorphic dinosaur skulls. *Nature* **487,** 223-226 (2012).
7. Klingenberg, C.P. & Marugán-Lobón, J. Evolutionary covariation in geometric morphometric data: analyzing integration, modularity and allometry in a phylogenetic context. *Syst. Biol.* **62,** 591-610 (2013).
8. Sherratt E., Gower, D.J., Klingenberg, C.P. & Wilkinson, M. Evolution of Cranial Shape in Caecilians (Amphibia: Gymnophiona). *Evol. Biol.* **41,** 528-545 (2014).
9. Longrich, N.R., Bhullar, B.A. & Gauthier, J.A. A transitional snake from the Late Cretaceous period of North America. *Nature* **488,** 205-208 (2012).
10. Apesteguía, S. & Zaher, H. A Cretaceous terrestrial snake with robust hindlimbs and a sacrum. *Nature* **440,** 1037-1040 (2006).
11. Martill, D.M., Tischlinger, H. & Longrich, N.R. A four-legged snake from the Early Cretaceous of Gondwana. *Science* **349,** 416-419 (2015).
12. Klingenberg, C.P. MorphoJ: an integrated software package for geometric morphometrics. *Mol. Ecol. Resour.* **11,** 353-357 (2011).
13. Maisano, J.A. & Rieppel, O. The skull of the round island boa, Casarea dussumieri Schlegel, based on high-resolution X-ray computed tomography. *J. Morphol.* **268,** 371-384 (2007).
14. Evans, S.E. The skull of lizards and Tuatara. In: Gans, C., Gaunt, A.S. & Adler, K. (eds). *Biology of the Reptilia* **20,** 1-347. Ithaca: New York Society for the Study of Amphibians and Reptiles (2008).
15. Cundall, D. & Irish, F. The snake skull. In: Gans, C., Gaunt, A.S. & Adler, K. (eds). *Biology of the Reptilia* **20,** 349-692. Ithaca: New York Society for the Study of Amphibians and Reptiles (2008).
16. Gauthier, J., Kearney, M., Maisano, J.A., Rieppel, O. & Behlke A. Assembling the squamate tree of life: perspectives from the phenotype and the fossil record. *Bull. Peabody Mus. Nat. Hist.* **53,** 3-308 (2012).
17. Olori, J.C. & Bell, C.J. Comparative skull morphology of uropeltid snakes (Alethinophidia: Uropeltidae) with special reference to disarticulated elements and variation. *PLoS One* **7,** e32450 (2012).
18. Rohlf, F.J. tpsDig, digitize landmarks and outlines v.2.17. Department of Ecology and Evolution, State University of New York at Stony Brook (2013).
19. Zelditch, M.L., Swiderski, D.L., Sheets, H.D. & Fink, W.L. *Geometric morphometrics for biologists: a primer*. San Diego: Elsevier (2004).
20. Klingenberg, C.P. Evolution and development of shape: integrating quantitative approaches. *Nat. Rev. Genet.* **11,** 623-635 (2010).
21. Jolliffe, I.T. Principal component analysis. *Springer Series in Statistics* (2002).
22. Rohlf, F. J. tpsSmall ver.1.29. Department of Ecology and Evolution, State University of New York at Stony Brook (2014).
23. Drake, A.G. & Klingenberg, C.P. The pace of morphological change: historical transformation of skull shape in St. Bernard dogs. *Proc. R. Soc. Lond.* **275,** 71-76 (2008).

24. Klingenberg, C.P. Visualizations in geometric morphometrics: how to read and how to make graphs showing shape changes. *Hystrix* **24,** 5-24 (2013).
25. Márquez, E.J., Cabeen, R., Woods, R.P. & Houle, D. The Measurement of Local Variation in Shape. *Evol. Biol.* **39,** 419-439 (2012).
26. Adams, D.C. A generalized K statistic for estimating phylogenetic signal from shape and other high-dimensional multivariate data. *Syst. Biol.* **63,** 685-97 (2014).
27. Adams, D.C. & Otárola-Castillo, E. Geomorph: an r package for the collection and analysis of geometric morphometric shape data. *Methods Ecol. Evol.* **4,** 393-399 (2013).
28. Felsenstein, J. Phylogenies and the comparative method. *Amer. Nat.* **125,** 1-15(1985).
29. Garland, T., Jr., Dickerman, A.W., Janis, C.M. & Jones, J.A. Phylogenetic analysis of covariance by computer simulation. *Syst. Biol.* **42,** 265-292 (1993)-Evans, S.E. 1980. The skull of a new eosuchian reptile from the Lower Jurassic of South Wales. In Conrad, J. L. Phylogeny and systematics of squamata (reptilia) based on morphology. Bull. Am. Mus. Nat. Hist. **310,** 1-182 (2008).
30. Harmon, L.J., Weir, J.T., Brock, C.D., Glor, R.E. & Challenger, W. GEIGER: investigating evolutionary radiations. *Bioinformatics* **24,** 129-31 (2008).
31. Timm, N.H. Applied multivariate analysis. *New York: Springer* (2002).
32. Stayton, C.T. The definition, recognition, and interpretation of convergent evolution, and two new measures for quantifying and assessing the significance of convergence. *Evolution* **69,** 2140-2153 (2015).
33. Caldwell, M.W., Nydam, R.L., Palci, A. & Apesteguía, S. The oldest known snakes from the middle Jurassic-Lower Cretaceous provide insights on snake evolution. **Nat. Commun. 6,** 5996 (2015).
34. Zaher, H. & Scanferla, C.A. The skull of the Upper Cretaceous snake Dinilysia patagonica Smith-Woodward 1901, and its phylogenetic position revisited. *Zool. J. Linnean. Soc.* **164,** 194-238 (2012).
35. Yi, H. & Norell, M.A. The burrowing origin of modern snakes. *Sci. Adv.* **1,** e1500743 (2015).
36. Scanlon, J.D. & Lee, M.S. The Pleistocene serpent Wonambi and the early evolution of snakes. *Nature* **403,** 416-420 (2000).
37. Scanlon, J.D. Skull of the large non-macrostomatan snake Yurlunggur from the Australian Oligocene. *Nature* **439,** 839-842 (2006).
38. Scanferla, A. *et al.* A new snake skull from the Paleocene of Bolivia sheds light on the evolution of macrostomatans. *PLoS One* **8,** e57583 (2013).
39. Caldwell, M.W. & Lee, M.S.Y. A snake with legs from the marine Cretaceous of the Middle East. *Nature* **386,** 705-709 (1997).
40. Tchernov, E., Rieppel, O., Zaher, H., Polcyn, M.J. & Jacobs, L.L. A Fossil Snake with Limbs. *Science* **287,** 2010-2012 (2000).
41. Rage, J.C. & Escuillié, F. Un nouveau serpent bipède du Cénomanien (Crétacé). Implications phylétiques. *C. R. Acad. Sci. Paris Earth Sci.* **330,** 513-520 (2000).
42. Polcyn M.J., Jacobs, L.L. & Haber, A.A. morphological model and CT assessment of the skull of Pachyrhachis problematicus (squamata, serpentes), a 98 million year old snake with legs from the Middle East. *Palaeontol. Electronica* **8,** 1-24 (2005).
43. Conrad, J.L. Phylogeny and systematics of squamata (reptilia) based on morphology. *Bull. Am. Mus. Nat. Hist.* **310,** 1-182 (2008).
44. Wilson, J.A., Mohabey, D.M., Peters, S.E. & Head, J.J. Predation upon hatchling dinosaurs by a new snake from the late Cretaceous of India. *PLoS Biol.* **8,** e1000322 (2010).
45. Wu, X.C., Brinkman, D.B. & Russell, A.P. Sineoamphisbaena hexatabularis hexatabularis: an amphisbaenian (Diapsida: Squamata) from the Upper Cretaceous redbeds at Bayan Mandahu (Inner Mongolia, People's Republic of China), and comments on the phylogenetic relationships of the Amphisbaenia. *Can. J. Earth Sci.* **33,** 541-577 (1996).
46. Berman, D.S. Spathorhynchus natronicus, a new species of rhineurid amphisbaenian (Reptilia) from the Early Oligocene of Wyoming. *J. Paleontol.* **51,** 986-991 (1977).

47. Kearney, M., Maisano, J.A. & Rowe, T. Cranial anatomy of the extinct amphisbaenian Rhineura hatcherii (Squamata, Amphisbaenia) based on high-resolution X-ray computed tomography. *J. Morphol.* **264,** 1-33 (2005).
48. Rieppel, O., Conrad, J.L. & Maisano, J.A. New morphological data for Eeosaniwa koehni haubold, 1977 and a revised phylogenetic analysis. *J. Paleont.* **81,** 760-769 (2007).
49. Lingham-Soliar, T. Anatomy and functional morphology of the largest marine reptile known: Mosasaurus hoffmanni (Mosasauridae, Reptilia) from the Upper Cretaceous, Upper Maastrichtian of The Netherlands. *Philos. Trans. R Soc. Lond. B Biol. Sci.* **347,** 155-180 (1995).
50. Montero, R., Gans, C. & Lions, M.L. Embryonic development of the skeleton of Amphisbaena darwini heterozonata (Squamata: Amphisbaenidae). *J. Morphol.* **239,** 1-25 (1999).
51. Klingenberg, C.P. & Gidaszewski, N.A. Testing and quantifying phylogenetic signals and homoplasy in morphometric data. *Syst. Biol.* **59**, 245-261 (2010).
52. Blomberg S.P., Lefevre J.G., Wells J.A. & Waterhouse M. Independent contrasts and PGLS regression estimators are equivalent. *Syst. Biol.* **61,** 382-391 (2012).
53. Müller, J. *et al.* Eocene lizard from Germany reveals amphisbaenian origins. *Nature* **473,** 364-367 (2011).
54. Maddison, W.P. (1991) Squared-change parsimony reconstructions of ancestral states for continuous-valued characters. *Syst. Zool.* **40,** 304-314 (1991).
55. Collyer, M.L. & Adams, D.C. Analysis of two-state multivariate phenotypic change in ecological studies. *Ecology* **88,** 683-692 (2007).
56. Collyer, M.L. & Adams, D.C. Phenotypic trajectory analysis: Comparison of shape change patterns in evolution and ecology. *Hystrix* **24,** 75-83 (2013).
57. Alberch, P., Gould, S.J., Oster, G.F. & Wake, D.B. Size and shape in ontogeny and phylogeny. *Paleobiology* **5,** 296-317 (1979).
58. Mitteroecker, P., Gunz, P. & Bookstein, F.L. Heterochrony and geometric morphometrics: a comparison of cranial growth in Pan paniscus versus Pan troglodytes. *Evol. Dev.* **7,** 244-58 (2005).
59. Werneburg, I. A standard system to study vertebrate embryos. *PLoS One* **4,** e5887 (2009).
60. Boback, S.M., Dichter, E.K. & Mistry, H.L. A developmental staging series for the African house snake, Boaedon (Lamprophis) fuliginosus. *Zoology (Jena)* **115,** 38-46 (2012).
61. Tinkle, D.W. & Gibbons, J.W. Distribution and Evolution of Viviparity in Reptiles. *Misc. Publ. Mus. Zool. Univ. Mich.* **154,** 1-55 (1977).
62. Köhler, G. Incubation of reptile eggs: basics, guidelines, experiences. *Krieger Publishing Company* (2005).
63. Maisano, J.A. A survey of state of ossification in neonatal squamates. *Herpetol. Monogr.* **15,** 135-157 (2001).
64. Maisano, J.A. The potential utility of postnatal skeletal developmental patterns in squamate phylogenetics. *Zool. J. Linnean. Soc.* **136,** 277-313 (2002).
65. Rieppel, O. Studies on Skeleton Formation in Reptiles. III. Patterns of Ossification in the Skeleton of Lacerta vivipara Jacquin (Reptilia, Squamata). *Zoology* **68,** 1-25 (1992).
66. Werneburg, I. & Sánchez-Villagra, M.R. Skeletal heterochrony is associated with the anatomical specializations of snakes among squamate reptiles. *Evolution* **69,** 254-63 (2015).
67. Rieppel, O. Studies on skeleton formation in reptiles. II. Chamaeleo hoehnelii (Squamata: Chamaeleoninae), with comments on the homology of carpal and tarsal Bones. *Herpetologica* **49,** 66-78 (1993).
68. Rieppel, O. The cranial morphology of the fossorial lizard genus Dibamus with a consideration of its phylogenetic relationships. *J. Zool.* **204,** 289-327 (1984a).
69. Sulimski, A. Adamisaurus magnidentatus n. gen., n. sp. (Sauria) from the Upper Cretaceous of Mongolia. *Palaeontol. Pol.* 27, 33-40 (1972).
70. Fernandez, V. *et al.* Evidence of Egg Diversity in squamate evolution from Cretaceous anguimorph embryos. *PLoS One* **10,** e0128610 (2015).

71. Gao, K. & Norell, M.A. Taxonomic composition and systematics of late cretaceous lizard assemblages from ukhaa tolgod and adjacent localities, mongolian gobi desert. *Bull. Am. Mus. Nat. Hist.* **249,** 1-118 (2000).
72. Reynoso, V.H. Huehuecuetzpalli mixtecus mixtecus gen. et sp. nov. a basal squamate (Reptilia) from the Early Cretaceous of Tepexide Rodríguez, Central México. *Philos. Trans. R Soc. Lond. B Biol. Sci.* **353,** 477-500 (1998).
73. Borsuk-Białynicka, M. & Moody, S.M. Priscagaminae: a new subfamily of the Agamidae (Sauria) from the Late Cretaceous of the Gobi Desert. *Acta Palaeontol. Pol.* **29,** 51-81 (1984).
74. Daza, J.D., Alifanov, V.R. & Bauer, A.M. A redescription and phylogenetic reinterpretation of the fossil lizard Hoburogekko suchanovi Alifanov, 1989 (Squamata, Gekkota), from the Early Cretaceous of Mongolia. *J. Vert. Paleontol.* **32,** 1303-1312 (2012).
75. Nance, H.A. Cranial osteology of the African gerrhosaurid Angolosaurus skoogi (Squamata; Gerrhosauridae). *Afr. J. Herpetol.* **56,** 39-75 (2007).
76. Tarazona, O.A. Cranial morphology of Bachia bicolor (Squamata:Gymnophthalmidae) and its postnatal development. *Zool. J. Linnean. Soc.* 152, 775-792 (2008).
77. Roscito, J.G. & Rodrigues, M.T. Skeletal development in the fossorial gymnophthalmids Calyptommatus sinebrachiatus and Nothobachia ablephara. *Zoology* **115,** 289-301 (2012).
78. Roscito, J.G. Desenvolvimento embrionário e a evolução da fossorialidade nos lagartos da tribo Gymnophthalmini (Squamata, Gymnophthalmidae). Doctoral thesis in Zoology, Instituto de Biociências, Universidade de São Paulo, São Paulo (2010).
79. Montero, R. Cranial anatomy of Euspondylus acutirostris (squamata: gymnophthalmidae) and its placement in a modern phylogenetic hypothesis. *Russ. J. Herpetol.* **9,** 215-228 (2002).
80. Bell, C.J., Evans, S.E. & Maisano, J.A. The skull of the gymnophthalmid lizard Neusticurus ecpleopus (Reptilia: Squamata). *Zool. J. Linnean. Soc.* **139,** 283-304 (2003).
81. Hernández-Jaimes, C., Jerez, A. & Ramírez-Pinilla, M.P. Embryonic development of the skull of the Andean lizard Ptychoglossus bicolor (Squamata, Gymnophthalmidae). *J. Anat.* **221,** 285-302 (2012).
82. Guerra, C. & Montero, R. The skull of Vanzosaura rubricauda (Squamata: Gymnophthalmidae). *Acta. Zool.* **90,** 359-371 (2009).
83. Guerra-Fuentes, R.A. Desenvolvimento embrionário do crânio do clado Iguania e sua contribuicão para a filogenia do grupo (Reptilia, Squamata). Doctoral thesis in Zoology - Instituto de Biociências, Universidade de São Paulo, São Paulo (2006).
84. Rieppel, O. Studies on skeleton formation in reptiles. Patterns of ossification in the skeleton of Lacerta agilis exigua Eichwald (Reptilia, Squamata). *J. Herpetol.* **28,** 145-153 (1994).
85. Lobo, F., Abdala, F. & Scrocchi, G. Desarrollo del esqueleto de Liolaemus scapularis (Iguania: Tropiduridae). *Bolletino del Museo Regionale di Scienze Naturali (Torino)* **13,** 77-104 (1995).
86. Daza, J.D., Herrera, A., Thomas, R. & Claudio, H.J. Are you what you eat? A geometric morphometric analysis of gekkotan skull shape. *Biol. J. Linnean Soc.* **97,** 677-707 (2009).
87. Rieppel, O. The structure of the skull and jaw adductor musculature of the Gekkota, with comments on the phylogenetic relationships of the Xantusiidae (Reptilia: Lacertilia). *Zool. J. Linn. Soc.* **82,** 291-318 (1984b).
88. Rieppel, O. The upper temporel arcade of lizards: an ontogenetic problem. *Rev. Suisse Zool.* **91,** 475-482 (1984c).
89. Hollenshead, M.G. Geometric morphometric analysis of cranial variation in the Egernia depressa (Reptilia: Squamata: Scincidae) species complex. *Rec. West. Aust. Mus.* **26,** 138-153 (2011).
90. Hugi, J. Heterochronic shifts in the ossification sequences of surface- and subsurface-dwelling skinks are correlated with the degree of limb reduction. *Zoology* **115,** 188-198 (2012).
91. Hugi, J., Mitgutsch, C. & Sánchez-Villagra, M.R. Chondrogenic and ossification patterns and sequences in White’s skink Liopholis whitii (Scincidae, Reptilia). *Zoosyst. Evol.* **86,** 21-32 (2010).

92. Jerez, A. Structural characteristics of the skeleton in Mabuya sp. (Squamata: Scincidae): a comparison with african scincids. *Actual. Biol.* **34,** 207-223 (2012).
93. Caut, S., Holden, M., Jowers, M.J., Boistel, R. & Ineich, I. Is Bocourt's Terrific Skink Really So Terrific? Trophic Myth and Reality. *PLoS One* **8,** e78638 (2013).
94. Bever, G.S., Bell, C.J. & Maisano, J.A. The ossified braincase and cephalic osteoderms of Shinisaurus crocodilurus. *Palaeontol. Electronica* **8,** 1-36 (2005).
95. Howes, G.B. & Swinnerton, H.H. On the development of the skeleton of the Tuatara Sphenodon punctatus, with remarks on the egg, on the hatchling, and on the hatched young. *Trans. Zool. Soc. London* **16,** 1-86 (1901).
96. Arias, F. & Lobo, F. Patrones de osificación en tupinambis merianae y Tupinambis rufescens (squamata: teiidae) y patrones generales en squamata. *Cuad. herpetol.* **20,** 3-23 (2006).
97. Maisano, J.A. Cranial anatomy of the spade-headed amphisbaenian Diplometopon zarudnyi (Squamata, Amphisbaenia) based on high-resolution X-ray computed tomography. *J. Morphol.* **267,** 70-102 (2006).
98. Werneburg, I., Polachowski, K.M. & Hutchinson, M.N. Bony skull development in the Argus monitor (Squamata, Varanidae, Varanus panoptes) with comments on developmental timing and adult anatomy. *Zoology* **118,** 255-80 (2015).
99. Rieppel, O. & Zaher, H. The development of the skull in Acrochordus granulatus (Schneider) (Reptilia: Serpentes), with special consideration of the otico-occipital complex. *J. Morphol.* **249,** 252-266 (2001).
100. Rieppel, O., Kley, N.J. & Maisano, J.A. Morphology of the Skull of the White-Nosed Blindsnake, Liotyphlops albirostris (Scolecophidia: Anomalepididae). *J. Morphol.* **270,** 536-557 (2009).
101. Rieppel, O. The performance of morphological characters in broad-scale phylogenetic analyses. *Biol. J. Linnean Soc.* **92,** 297-308 (2007).
102. Wellman, J. A revision of snakes of the genus Conophis (family Colubridae, from Middle America). *Univ. Kansas Publ. Mus. Nat. Hist.* **15,** 251-295 (1963).
103. Cundall, D. & Rossman, D.A. Quantitative Comparisons of Skull Form in the Colubrid Snake Genera Farancia and Pseudoeryx. *Herpetologica* **40,** 388-405 (1984).
104. Di Pietro, D.O., Alcalde, L. & Williams, J.D. Nasal cartilages, hyobranchial apparatus, larynx, and glottal tubes in four species of Hydropsini (Serpentes: Dipsadidae: Xenodontinae). *Vertebr. Zool.* **64,** 103-111 (2014).
105. Deufel, A. & Cundall, D. Prey Transport in "Palatine-Erecting" Elapid Snakes. *J. Morphol.* **258,** 358-375 (2003).
106. Albuquerque, N.R. Osteologia craniana, morfologia do hemipênis e o posicionamento sistemático do gênero Hydrops Wagler, 1830 (Serpentes: Colubridae). *Comun. Mus. Ciênc. Tecnol. PUCRS, Sér. Zool.* **15,** 41-54 (2002).
107. Scanferla, A. & Bhullar, B.A. Postnatal development of the skull of Dinilysia patagonica (Squamata-stem serpentes). *Anat. Rec.* **297,** 560-573 (2014).
108. Kamal, A.M. & Hammouda, H.G. The Development of the Skull of Psammophis sibilans. III. The osteocranium of a late embryo. *J. Morphol.* **116,** 297-310 (1965).
109. Lee, M.S & Scanlon, J.D. Snake phylogeny based on osteology, soft anatomy and ecology. *Biol. Rev. Camb. Philos. Soc.* **77,** 333-401(2002).
110. Boughner, J.C. Embryonic development of Python sebae - I: Staging criteria and macroscopic skeletal morphogenesis of the head and limbs. *Zoology* **110,** 212-230 (2007).
111. Rieppel, O. & Zaher, H. The skull of the Uropeltinae (Reptilia, Serpentes), with special reference to the otico-occipital region. *Bull. Am. Mus. Nat. Hist.* **68,** 123-130 (2002).
112. Polachowski, K.M. & Werneburg, I. Late embryos and bony skull development in Bothropoides jararaca (Serpentes, Viperidae). *Zoology* **116,** 36-63 (2013).
113. Lima, F.C. Ontogenia de Iguana iguana (Linnaeus, 1758): estágios embrionários e desenvolvimento do esqueleto. Doctoral thesis (2015).

114. Brock, G.T. The skull of Acontias meleagris, with a study of the affinities between lizards and snakes. *Zool. J. Linnean. Soc.* **41,** 71-88 (1941).
115. Brock, G.T. On the development of the skull in Leptodeira hotamboia. *Q. J. Microsc. Sci.* **73,** 289-331 (1929).
116. Khannoon, E.R. & Evans, S.E. The Development of the skull of the egyptian cobra Naja h. haje (Squamata: Serpentes: Elapidae). *PLoS One* **10,** e0122185 (2015).
117. Haluska, F. & Alberch, P. The Cranial Development of Elaphe obsoleta (Ophidia, Colubridae). *J. Morphol.* **178,** 37-55 (1983).
118. Holliday, C.M. & Witmer, L.M. Archosaur Adductor Chamber Evolution: Integration of Musculoskeletal and Topological Criteria in Jaw Muscle Homology. *J. Morphol.* **268,** 457-484 (2007).
119. Jackson, K. Post-ovipositional development of the monocled cobra, Naja kaouthia (Serpentes: Elapidae). *Zoology* **105,** 203-214 (2002).
120. Ramaswami, L.S. The chondrocranium of Calotes versicolor (Daud.) with a description of the osteocranium of a just-hatched young. *Quart. J. Micr. Sci.* **87,** 237-297(1946).
121. Rieppel, O. & Maisano, J.A. The skull of the rare Malaysian snake Anomochilus leonardi Smith, based on high-resolution X-ray computed tomography. *Zool. J. Linnean. Soc.* **149,** 671-685 (2007).
122. Witten, G.J. Family Agamidae. In: Glasby, C.J., Ross, G.J.B. & Beesley, P.L. (eds). *Fauna of Australia* **2A**. AGPS Canberra (1993).
123. Bates, M.F. & de Villiers, A. *Agama hispida*. *The IUCN Red List of Threatened Species* **2017,** e.T170370A110309904 (2017).
124. Leaché, A.D. *et al.* Phylogeny of the genus *Agama* based on mitochondrial DNA sequence data. *Bonn. Zool. Beitr.* **56,** 273-278 (2009).
125. Sharma, R.C. Fauna of India and adjacent countries. Reptilia Volume II (Sauria). *Zoological Survey of India, Kolkata* (2002).
126. Collar, D.C., Schulte II, J.A., O'mera, B.C. & Losos J.B. Habitat use affects morphological diversification in dragon lizards. *J. Evol. Biol.* **23,** 1033-1049 (2010).
127. Agarwala, B.K. & Majumber, J. Calotes emma Gray, 1845 (Squamata: Agamidae): Range extension and new addition to the reptilian fauna of Tripura, northeast India. *Check List* **11,** 1562 (2015).
128. McGuire, J.A. & Dudley, R. The biology of gliding in flying lizards (Genus Draco) and their fossil and extant analogs. *Integr. Comp. Biol.* **51,** 983-990 (2011).
129. Ledesma, M., Brown, R., Sy, E. & Rico, E.L. *Hydrosaurus pustulatus*. *The IUCN Red List of Threatened Species* **2009,** e.T10335A3194587 (2009).
130. Manthey, U. & Denzer, W. A revision of the Melanesian-Australian Angle Head lizards of the genus *Hypsilurus* (Sauria: Agamidae: Amphibolurinae) with description of four new species and one new subgenus. *Hamadryad* **30,** 1-40 (2006).
131. Srikulnath, K. *et al.* Chromosomal localization of the 18S-28S and 5S rRNA genes and (TTAGGG)*n* sequences of butterfly lizards (*Leiolepis belliana belliana* and *Leiolepis boehmei*, Agamidae, Squamata). *Genet. Mol. Biol.* **34,** 582-586 (2011).
132. Krysko, K. & Enge, K.M. A new non-native lizard in Florida, the butterfly lizard, *Leiolepis belliana* (Sauria: Agamidae). *Florida Scientist* **68,** 247-249 (2005).
133. Darevsky, I.S. & Kupriyanova, L.A. Two new all-female lizard species of the genus *Leiolepis cuvier*, 1829 from Thailand and Vietnam (Squamata: Sauria: Uromastycinae). *Herpetozoa* **6,** 3-20 (1993).
134. Hartmann, T., Geissler, P. & Böhme, W. *Leiolepis* (Squamata: Agamidae) farming in southern Vietnam and a new size record in butterfly lizards. *Herpetological Bulletin* **117,** 15-18 (2011).
135. Balian, E.V., Lévêque, C., Segers, H. & Martens, K. Freshwater animal diversity assessment. *Hydrobiologia* **595,** 581-586 (2008).

136. Das, S.K., Dookia, S., Das, K. & Dutta, S.K. Ecological observations on the Indian spiny-tailed lizard *Saara hardwickii* (Gray, 1827) (Reptilia: Squamata: Agamidae) in Tal Chhapar wildlife sanctuary, Rajasthan, India. *J. Threat. Taxa* **5,** 3516-3526 (2013).
137. Hashmi, M.U.A, Khan, M.Z., Huda, N., Gabol, K. & Safi, A. Observation of the status, distribution, habitat and population estimation of the Indian spiny tailed lizard *Saara hardwickii* (Gray, 1827) of Thatta district of Sindh Pakistan. *American Journal of Zoological Research* **2,** 46-50 (2014).
138. Kearney, M. Systematics of the Amphisbaenia (Lepidosauria: Squamata) based on morphological evidence from recent and fossil forms. *Herpet. Monog.* **17,** 1-74 (2003).
139. Colli, G.R. & Zamboni, D.S. Ecology of the worm-lizard *Amphisbaena alba* in the cerrado of central Brazil. *Copeia* **3,** 733-742 (1999).
140. Agasyan, A. *et al. Anguis fragilis*. *The IUCN Red List of Threatened Species* **2009,** e.T157249A5060016 (2009).
141. Savage, J.M., Lips, K.R. & Ibanez, D.R. A new species of *Celestus* from west-central Panama, with consideration of the status of the genera of the Anguidae: Diploglossinae (Squamata). *Rev. Biol. Trop*. **56,** 845-859 (2008).
142. Vanzolini, P.E. Miscelaneus notes on the ecology of some brazilian lizards (Sauria). *Papeis Avulsos de Zoologia* **26,** 83-115 (1972).
143. Vitt, L.J. On the biology of the little known anguid lizards, *Diploglossus lessonae* in northeast Brazil. *Papeis Avulsos de Zoologia* **36,** 69-76 (1985).
144. Grismer, L.L. Amphibians and reptiles of Baja California, including its Pacific islands. *University of California Press* (2012).
145. Bartlett, R.D. & Bartlett, P.P. A field guide to Texas reptiles and amphibians. *Gulf Publishing Company, Houston, Texas* (1999).
146. Smíd, J. *et al.* Annotated checklist and distribution of the lizards of Iran. *Zootaxa* **3855,** 1-97 (2014).
147. Rifai, L. *et al. Pseudopus apodus* (PALLAS, 1775) from Jordan, with notes on its ecology. *Herpetozoa* **18,** 133-140 (2005).
148. Bury, R.B. & Balgooyen, T.G. Temperature selectivity in the legless lizard, *Anniella pulchra*. *Copeia* **1976,** 152-155 (1976).
149. Hunt, L.E. A nomenclatural rearrangement of the genus *Anniella* (Sauria: Anniellidae). *Copeia* **1983,** 79-89 (1983).
150. Hollingsworth, B. & Frost, D.R. *Bipes biporus*. *The IUCN Red List of Threatened Species* **2007,** e.T63723A12710548 (2007).
151. Ponce-Campos, P. & García Aguayo, A. *Bipes canaliculatus*. *The IUCN Red List of Threatened Species* **2007,** e.T63724A12710708 (2007).
152. Segall, M., Tolley, K.A., Vanhooydonck, B., Measey, G.J. & Herrel A. Impact of temperature on performance in two species of South African dwarf chameleon, *Bradypodion pumilum* and *B. occidentale*. *J. Exp. Biol.* **216,** 3828-3836 (2013).
153. Tilbury, C.R. Chameleons of Africa: An Atlas, Including the Chameleons of Europe, the Middle East and Asia. *Edition Chimaira, Frankfurt* (2010).
154. Boycott R.C. Observations on the African grass lizards *Chamaesaura Fitzinger (*Reptilia*:* Sauria*:* Cordylidae*)* in Swaziland with emphasis on fire impacts on populations in Malolotja Nature Reserve. *Durban Natural Science Museum Novitates* **37,** 30-39 (2015).
155. Savage, J.M. The amphibians and reptiles of Costa Rica: a herpetofauna between two continents, between two seas. *University of Chicago Press, Chicago* (2002).
156. Vitt, L.J. & Zani, P.A. Prey use among sympatric lizard species in lowland rain forest of Nicaragua. *J. Trop. Ecol.* **14,** 537-559 (1998).
157. Hammerson, G.A., Lavin, P., Vazquez Díaz, J., Quintero Díaz, G. & Gadsden, H. *Crotaphytus collaris*. *The IUCN Red List of Threatened Species* **2007,** e.T64007A12734318 (2007).

158. Frost, D.R. & Hammerson, G.A. *Anolis carolinensis*. *The IUCN Red List of Threatened Species* **2007,** e.T64188A12745542 (2007).
159. Williams E.E. Ecomorphs, faunas, island size, and diverse endpoints in island radiations of *Anolis*. In: Huey R.B., Pianka E.R. & Schoener T.W. (eds.). *Lizard ecology: studies of a model organism*. Harvard University Press, Cambridge, Massachusetts (1983).
160. Rieppel, O. The cranial morphology of the fossorial lizard genus *Dibarnus* with a consideration of its phylogenetic relationships. *J. Zool., Lond.* **204,** 289-327 (1984).
161. King, M. & Horner, P. Family Gekkonidae. In: Glasby, C.J., Ross, G.J.B. & Beesley, P.L. (eds). *Fauna of Australia* **2A**. AGPS Canberra (1993).
162. Swan, G. & Foster, R. The reptiles and amphibians of Mutawintji national park, Western New South Wales. *Australian Zoologist* **33,** 39-48 (2005).
163. Peattie, A.M. Subdigital setae of narrow-toed geckos, including a Eublepharid (*Aeluroscalabotes felinus*). *Anat. Rec.* **291,** 869-875 (2008).
164. Hammerson, G.A., Frost, D.R. & Gadsden, H. *Coleonyx variegatus*. *The IUCN Red List of Threatened Species* **2007,** e.T64039A12739050 (2007).
165. Khan, M.S. Natural history and biology of hobbyist choice leopard gecko *Eublepharis macularius*. *Reptilia* 1-16 (2004).
166. Penner, J., Rödel, M.O., Luiselli, L. & Segniagbeto, G. *Hemitheconyx caudicinctus*. *The IUCN Red List of Threatened Species* **2013,** e.T203830A2771717 (2013).
167. Cope, E.D. Synopsis of new vertebrata from the tertiary of Colorado obtained during the summer of 1873. *Seventh Annual Report of the United States Geological Survey of the Territories* (1973).
168. Jones, M.E.H. Skull shape and feeding strategy in Sphenodon and other Rhynchocephalia (Diapsida: Lepidosauria). *J. Morphol.* **269,** 945-966 (2008).
169. Borsuk-Bialynicka, M. *Globaura venusta* gen. et sp. n. and *Eoxanta lacertifrons* gen. et sp. n. - non-teiid lacertoids from the Late Cretaceous of Mongolia. *Acta Palaeontol. Pol.* **33,** 211-248 (1988).
170. Reynoso, V.H. *Huehuecuetzpali mixtecus* gen. et sp. nov: a basal squamate (Reptilia) from the Early Cretaceous of Tepexi de Rodríguez, Central México. *Philos. Trans. R. Soc. Lond. B Biol. Sci.* **353,** 477–500 (1998).
171. Cope, E.D. On the reptilian orders Pythonomorpha and Streptosauria. *Proceedings of the Boston Society of Natural History* **12,** 250-266 (1869).
172. Baig, K.J., Awan, M.R. & Ashraf N. Ecological studies and zoogeographic affinities of the amphibians and reptiles found in Chagai desert, Balochistan, Pakistan. *Pakistan J. Zool.* **38,** 145-151 (2006).
173. Gardner, D. Terrestrial reptiles in The Emirates a natural history. In: Hellyer, P. & Aspinall, S. (eds). *Trident press Limited* (2005).
174. Hoskin, C.J. The invasion and potential impact of the Asian House Gecko (*Hemidactylus frenatus*) in Australia. *Austral Ecology* **36,** 240- 251 (2011).
175. Vences, M. *Phelsuma lineata*. *The IUCN Red List of Threatened Species* **2011,** e.T172826A6925369 (2011).
176. Polakow, D.A. Communication and sexual selection in the barking gecko (*Ptenopus kochi*). *Thesis (M.Sc. (Zoology)), University of Cape Town* (1997).
177. Whitaker, A.H., Sadlier, R.A. & Bauer A.M. *Rhacodactylus auriculatus*. *The IUCN Red List of Threatened Species* **2010,** e.T176132A7185707 (2010).
178. Adolphs, K. & Bates, M.F. *Cordylosaurus subtessellatus*. *The IUCN Red List of Threatened Species* **2010,** e.T178341A7526963 (2010).
179. Bates, M.F. *et al.* A molecular phylogeny of the African plated lizards, genus *Gerrhosaurus* Wiegmann, 1828 (Squamata: Gerrhosauridae), with the description of two new genera. *Zootaxa* **3750,** 465-493 (2013).
180. Ramos-Pallares, E. *et al.* Feeding and reproductive ecology of *Bachia bicolor* (Squamata: Gymnophthalmidae) in urban ecosystems from Colombia. *J. Herpetol.* **49,** 108-117 (2015).

181. Roscito, J.G. & Rodrigues, M.T. Comparative cranial osteology of fossorial lizards from the tribe gymnophthalmini (Squamata, Gymnophthalmidae). *J. Morphol.* **271,** 1352-1365 (2010).
182. Barros, F.C., Herrel, A. & Kohlsdorf. T. Head shape evolution in Gymnophthalmidae: does habitat use constrain the evolution of cranial design in fossorial lizards? *J. Evol. Biol.* **24,** 2423-2433 (2011).
183. Chávez, G., Siu-Ting, K., Duran, V., Venegas, P.J. Two new species of Andean gymnophthalmid lizards of the genus *Euspondylus* (Reptilia, Squamata) from central and southern Peru. *Zookeys* **109,** 1-17 (2011).
184. Cisneros-Heredia, D.F. *Pholidobolus montium*. *The IUCN Red List of Threatened Species* **2015,** e.T44578680A44578689 (2015).
185. Anaya-Rojas, J.L. & Serrano-Cardozo, V.H. Diet, microhabitat use, and thermal preferences of *Ptychoglossus bicolor* (Squamata: Gymnophthalmidae) in an organic coffee shade plantation in Colombia. *Papeis Avulsos de Zoologia* **50,** 159-166 (2010).
186. Beck, D.D. & Lowe, C.H. Ecology of the beaded Lizard, *Heloderma horridum*, in a tropical dry forest in Jalisco, Mexico. *J. Herpetol.* **25,** 395-406 (1991).
187. Gienger, C.M. & and Tracy, C.R. Ecological interactions between Gila monsters (*Heloderma suspectum*) and desert tortoises (*Gopherus agassizii*). *The Southwestern Naturalist* **53,** 265-268 (2008).
188. Ribeiro-Júnior, M.A. Catalogue of distribution of lizards (Reptilia: Squamata) from the Brazilian Amazonia. I. Dactyloidae, Hoplocercidae, Iguanidae, Leiosauridae, Polychrotidae, Tropiduridae. *Zootaxa* **3983,** 1-110 (2015).
189. Torres-Carvajal, O., Almendáriz, A., Valencia, J., Yánez-Muñoz, M. & Reyes, J.P. A new species of *Enyalioides* (Iguanidae: Hoplocercinae) from southwestern Ecuador. *Papeis Avulsos de Zoologia* **48,** 227-235 (2007).
190. Pianka, E.R. & Vitt, P.L. Lizards: windows to the evolution of diversity. *University of California Press* (2003).
191. Fisher, R., Grant, T. & Harlow, P. *Brachylophus fasciatus*. *The IUCN Red List of Threatened Species* **2012,** e.T19243030A2791124 (2012).
192. Townsend, J.H., Krysko, K.L. & Enge, K.M. The identity of spiny-tailed Iguanas, *Ctenosaura*, introduced to Florida, USA (Squamata: Sauria: Iguanidae). *Herpetozoa* **16,** 67-72 (2003).
193. Cowles, R.B. & Bogert, C.M. Preliminary study of the thermal requirements of desert reptiles. *Bull. Am. Mus. Nat. Hist.* **83,** 261-296 (1944).
194. Vitt, L.J. & Zani, P.A. Prey use among sympatric lizard species in lowland rain forest of Nicaragua. *J. Trop. Ecol.* **14,** 537-559 (1998).
195. Irish, J. *Ichnotropis capensis* (Smith 1838) in Namibia. *Namibia Biodiversity Database Web Site* (2012).
196. Isailovic, J.C. *et al. Lacerta viridis*. *The IUCN Red List of Threatened Species* **2009,** e.T61530A12507156 (2009).
197. Ineich, I., Chirio, L., Ascani, M., Rabeil, T. & Newby, J. Herpetofauna of termit massif and neighbour areas in Tenere desert, southeastern Niger, West Africa. *Herpetol. Notes* **7,** 375-390 (2014).
198. Miras, J.A.M. *et al. Psammodromus algirus*. *The IUCN Red List of Threatened Species* **2009,** e.T61558A12491246 (2009).
199. Amat Orriols, F. Preliminary analysis of correlated evolution of morphology and ecological diversification in lacertid lizards. *Butll. Soc. Cat. Herp*. 19 (2011).
200. Gifford, M.E., Herrel, A. & Mahler, D.L. The evolution of locomotor morphology, performance, and anti-predator behaviour among populations of *Leiocephalus* lizards from the Dominican Republic. *Biol. J. Linn. Soc.* **93,** 445-456 (2008).
201. Arzamendia, V. *et al. Anisolepis longicauda*. *The IUCN Red List of Threatened Species* **2017,** e.T203135A2761080 (2017).
202. Espinoza, R. & Cruz, F.B. *Leiosaurus catamarcensis*. *The IUCN Red List of Threatened Species* **2010,** e.T178320A7522348 (2010).

203. Espinoza, R. *Pristidactylus torquatus*. *The IUCN Red List of Threatened Species* **2010,** e.T178512A7561781 (2010).
204. Sousa, B.M., Gomides, S.C., Hudson, A.A., Ribeiro, L.B. & Novelli, I.A. Répteis do município de Juiz de Fora, Minas Gerais, Brasil. *Biota Neotrop.* **12,** 35-49 (2012).
205. Schulte, J.A. II, Losos, J.B., Cruz, F.B. & Núñez, H. The relationship between morphology, escape behaviour and microhabitat occupation in the lizard clade *Liolaemus* (Iguanidae: Tropidurinae: Liolaemini). *J. Evol. Biol.* **17,** 408-420 (2004).
206. Vidal, M.A. Habit, E., Victoriano, P., González-Gajardo, A. & Ortiz, J.C. Thermoregulation and activity pattern of the high-mountain lizard *Phymaturus palluma* (Tropiduridae) in Chile. *Zoologia* **27,** 13-18 (2010).
207. Vences, M. *Chalarodon madagascariensis*. *The IUCN Red List of Threatened Species* **2011,** e.T172849A6929237 (2011).
208. Münchenberg, T., Wollenburg, K.C., Glaw, F. & Vences, M. Molecular phylogeny and geographic variation of Malagasy iguanas (*Oplurus* and *Chalarodon*). *Amphib-Reptil.* **29,** 319-327 (2008).
209. Grismer, L.L. Amphibians and reptiles of Baja California, including its Pacific islands and the islands in the sea of Cortés. *University of California Press* (2002).
210. Hammerson, G.A., Frost, D.R. & Gadsden, H. *Phrynosoma platyrhinos*. *The IUCN Red List of Threatened Species* **2007,** e.T64080A12734576 (2007).
211. Chaves, G., Lamar, W., Porras, L.W. & Sunyer, J. *Sceloporus variabilis*. *The IUCN Red List of Threatened Species* **2013,** e.T198414A2525951 (2013).
212. Gottschol, A.D., Marks, S.B. & Jennings, W.B. Speciation, population structure, and demographic history of the Mojave Fringe-toed Lizard (*Uma scoparia*), a species of conservation concern. *Ecol. Evol.* **4,** 2546-2562 (2014).
213. Tinkle, D.W., McGregor, D. & Dana, S. Home range ecology of *Uta Stansburiana* Stejnegeri. *Ecology* **43,** 223-229 (1962).
214. Diaz, L.M. & Hedges, B. A new gecko of the genus *Tarentola* (Squamata: Gekkonidae) from eastern Cuba. *Zootaxa* **1743,** 43-52 (2008).
215. Koch, C., Venegas, P.J., Garcia-Bravo, A. & Böhme, W. A new bush anole (Iguanidae, Polychrotinae, *Polychrus*) from the upper Marañon basin, Peru, with a redescription of *Polychrus peruvianus* (Noble, 1924) and additional information on *P. gutturosus* Berthold, 1845. *Zookeys* **141,** 79-107 (2011).
216. Maryan, B., How, R.A., & Adams, M. A new species of the *Aprasia repens* species-group (Squamata: Pygopodidae) from western Australia. *Rec. West Aust. Mus.* **28,** 30-43 (2013).
217. Cogger, H.G. *Delma torquata*. *The IUCN Red List of Threatened Species* **2010,** e.T6317A12626870 (2010).
218. Wall, M. & Shine, R. Ecology and behaviour of burton's legless lizard (*Lialis burtonis*, Pygopodidae) in tropical Australia. *Asian Herpetol. Res.* **4,** 9-21 (2013).
219. Kearney, M. Systematics of the Amphisbaenia (Lepidosauria: Squamata) based on morphological evidence from recent and fossil forms. *Herpet. Monog.* **17,** 1-74 (2003).
220. Daniels, S.R., Heidman, N., Hendricks, M. & Willsond, B. A molecular phylogeny for the South African limbless lizard taxa of the subfamily Acontinae (Sauria: Scincidae) with special emphasis on relationships within *Acontias*. *Mol. Phylogenet. Evol.* **24,** 315-323 (2002).
221. Raxworthy, C.J., Ramanamanjato, J.B. & Randriamahazo, H. *Amphiglossus splendidus*. *The IUCN Red List of Threatened Species* **2011,** e.T172908A6939782 (2011).
222. Siler, C.D., Rico, E.L., Duya, M.R. & Brown, R.M. A new limb-reduced, loamswimming skink *(*Squamata*:* Scincidae*: Brachymeles)* from Central Luzon Island, Philippines. *Herpetologica* **65,** 449-459 (2009).
223. Brown, R., Ledesma, M. & Jose, R. *Brachymeles gracilis*. *The IUCN Red List of Threatened Species* **2009,** e.T169761A6670501 (2009).
224. Çiçek, K. & Göçmen, B. Food composition of ocellated Skink, *Chalcides ocellatus* (Forskal, 1775) (Squamata: Scincidae), from the Cyprus Island. *Acta Herpetol.* **8,** 167-170 (2013).

225. Pianka, E.R. Notes on the ecology and natural history of two species of *Egernia* (Scincidae) in Western Australia. *West. Aust. Nat.* **30,** 231-236 (2014).
226. Cogger, H.G. Reptiles and amphinians of australia 7th edition. *CSIRO* (2014).
227. Geniez, P. *et al. Eumeces algeriensis*. *The IUCN Red List of Threatened Species* **2009,** e.T61499A12483547 (2009).
228. Çiçek, K., Cumhuriyet, O., Bayrakci, Y. & Ayaz. D. New locality records of *Eumeces schneideri* (Daudin, 1802) (Sauria: Scincidae) from western Anatolia, Turkey. *Turk. J. Zool.* **39,** 987-990 (2015).
229. Smyth, M. The distribution and life history of the skink, *Hemiergis peronii* (Fitzinger). *Trans. R. Soc. S* **92,** 51-58 (1968).
230. Gardner, M.G., Hugall, A.F., Donnellan, S.C., Hutchinson, M.N. & Foster, R. Molecular systematics of social skinks: phylogeny and taxonomy of the *Egernia* group (Reptilia: Scincidae). *Zool. J. Linnean. Soc.* **154,** 781-794 (2008).
231. Chapple, D.G. *et al.* Evolution and maintenance of colour pattern polymorphism in *Liopholis* (Squamata: Scincidae). *Aust. J. Zool.* **56,** 103-115 (2008).
232. Vitt, L.J. & Blackburn, D.G. Ecology and life history of the viviparous lizard *Mabuya bistriata* (Scincidae) in the Brazilian Amazon. *Copeia* **4,** 916-927 (1991).
233. Spawls, S. *Mochlus sundevalli*. *The IUCN Red List of Threatened Species* **2010,** e.T178641A7586633 (2010).
234. Kennedy, A.M., Marais, J., Bauer, A.M., Lewis, P.J. & Thies, M.L. Effect of fire on the herpetofauna of the Koanaka hills, Ngamiland, Botswana. *Check List* **8,** 666-674 (2012).
235. Caut, S., Holden, M., Jowers, M.J., Boistel, R. & Ineich, I. Is Bocourt's terrific skink really so terrific? Trophic myth and reality. *PLoS One* **8,** e78638 (2013).
236. Young, B.A. & Morain, M. The use of ground-borne vibrations for prey localization in the Saharan sand vipers (*Cerastes*). *J. Exp. Biol.* **205,** 661-665 (2002).
237. Linkem, C.W., Diesmas, A.C. & Brown, R.M. Molecular systematics of the Philippine forest skinks (Squamata: Scincidae: *Sphenomorphus*): testing morphological hypotheses of interspecific relationships. *Zool. J. Linnean. Soc.* **163,** 1217-1243 (2011).
238. Koenig, J., Shine, R. & Shea, G. The ecology of an Australian reptile icon: how do blue-tongued lizards (*Tiliqua scincoides*) survive in suburbia? *Wildlife Research* **28,** 215-227 (2001).
239. Hoinsoude, G. *et al.* Checklist of the lizards of Togo (West Africa), with comments on systematics, distribution, ecology, and conservation. *Zoosystema* **37,** 381-402 (2015).
240. Huang, C.M. *et al.* Population and conservation strategies for the Chinese crocodile lizard (*Shinisaurus crocodilurus*) in China. *Animal Biodiversity and Conservation* **31.2** (2008).
241. Domínguez-López, M.E. *et al.* Tail autotomy effects on the escape behavior of the lizard *Gonatodes albogularis* (Squamata: Sphaerodactylidae), from Córdoba, Colombia. *Revista Chilena de Historia Natural* **88** (2015).
242. Ananjeva, N. *Teratoscincus przewalskii*. *The IUCN Red List of Threatened Species* **2010,** e.T178359A7530815 (2010).
243. Corkery, I., Bell, B.D. & Nelson, N.J. Behavioral thermoregulation of the tuatara, *Sphenodon punctatus,* under hydric and digestive constraints. *Herpetol. Conserv. Biol.* **9,** 29-37 (2014).
244. Hammerson, G.A., Frost, D.R. & Santos-Barrera, G. *Aspidoscelis tigris*. *The IUCN Red List of Threatened Species* **2007,** e.T64290A12754666 (2007).
245. Vidal, M.A., Pizarro-Araya, J. Jerez, V. & Ortiz, J.C. Daily activity and thermoregulation in predatoreprey interaction during the flowering desert in Chile. *J. Arid Environ.* **75,** 802-808 (2011).
246. Vitt, L.J. Sartorius, S.S., Avila-Pires, T.C.S. & Espósito, M.C. Life at the river's edge: ecology of *Kentropyx altamazonica* in Brazilian Amazonia. *Can. J. Zool.* **79,** 1855-1865 (2001).
247. Embert, D., Fitzgerald, L. & Waldez, F. *Salvator merianae*. *The IUCN Red List of Threatened Species* **2010,** e.T178340A7526681 (2010).
248. Scott, N. *et al. Salvator merianae*. *The IUCN Red List of Threatened Species* **2016,** e.T178340A61322552 (2016).

249. Pelegrin, N., Chani, J.M., Echevarría, A.L. & Bucher, E.H. Effects of forest degradation on abundance and microhabitat selection by ground dwelling Chaco lizards. *Amphib-Reptil* **30,** 265-271 (2009).
250. Kearney, M. Systematics of the Amphisbaenia (Lepidosauria: Squamata) based on morphological evidence from recent and fossil forms. *Herpet. Monog.* **17,** 1-74 (2003).
251. Vitt, L.J. Ecology and life history of the scansorial arboreal lizard *Plica plica* (Iguanidae) in Amazonian Brazil. *Can. J. Zool.* **69,** 504-511 (1991).
252. Torres-Carvajal, O. Ecuadorian lizards of the genus *Stenocercus* (Squamata: Tropiduridae). *Natural History Museum, The University of Kansas* (2000).
253. Teixeira, R.L. & Giovanelli, M. Ecologia de *Tropidurus torquatus (*Sauria: Tropiduridae*)* da Restinga de Guriri, São Mateus, ES. *Rev. Brasil. Biol.* **59,** 11-18 (1999).
254. Cassimiro, J., Teixeira Jr, M., Recoder, R.S. & Rodrigues, M.T. *Tropidurus montanus* (Calango-da-Montanha; Montane collared lizard). *Herpetological Review* **40** (2009).
255. Bennett, D. & Sweet, S.S. *Varanus primordius*. *The IUCN Red List of Threatened Species* **2010,** e.T178030A7487647 (2010).
256. Bennett, D. & Sweet, S.S. *Varanus exanthematicus*. *The IUCN Red List of Threatened Species* **2010,** e.T178346A7527972 (2010).
257. Bennett, D. & Sweet, S.S. *Varanus rosenbergi*. *The IUCN Red List of Threatened Species* **2010,** e.T178031A7488030 (2010).
258. Bennett, D., Gaulke, M., Pianka, E.R., Somaweera, R. & Sweet, S.S. *Varanus salvator*. *The IUCN Red List of Threatened Species* **2010,** e.T178214A7499172 (2010).
259. Savage, J.M. Studies on the lizard family Xantusiidae IV. The genera. *Los Angeles Co. Mus., Contrib. Sci.* **71,** 1-38 (1963).
260. Bezy, R.L. & Camarillo, J.L. Systematics of xantusiid lizards of the genus *Lepidophyma. Contributions in Science* **493,** 1-41 (2002).
261. Papenfussi, T., Macey, J.R. & Schulte II, J.A. A new lizard species in the genus *Xantusia* from Arizona. *Natural History Museum, The University of Kansas* **23,** 1-9 (2001).
262. Hollingsworth, B. & Hammerson, G.A. *Xantusia henshawi*. *The IUCN Red List of Threatened Species* **2007,** e.T64366A12774257 (2007).
263. Lemos-Espinal, J.A., Smith, G.R. & Ballinger, R.E. Ecology of *Xenosaurus grandis* agrenon, a knob-scaled lizard from Oaxaca, México. *J. Herpetol.* **37,** 192-196 (2003).
264. Lawing, A.M., Head, J.J. & Polly, P.D. The ecology of morphology: the ecometrics of locomotion and macroenvironment in North American snakes. In: Louys, L. (ed). *Paleontology in Ecology and Conservation*. Springer-Verlag, Berlin and Heidelberg (2012).
265. Sanders, K., Murphy, J., Lobo, A. & Gatus, J. *Acrochordus granulatus*. *The IUCN Red List of Threatened Species* **2010,** e.T176769A7300762 (2010).
266. Maschio G.F., Prudente, A.L., Rodrigues F. & Hoogmoed, M.S. Food habits of *Anilius scytale* (Serpentes: Aniliidae) in the Brazilian Amazonia. *Zoologia* **27,** 184-190 (2010).
267. Adalsteinsson, S.A., Branch, W.R., Trape, S., Vitt, L.J. & Hedges, S.B. Molecular phylogeny, classification, and biogeography of snakes of the family Leptotyphlopidae (Reptilia, Squamata). *Zootaxa* **2244,** 1-50 (2009).
268. Bruner, G., Fernández-Marín, H., Touchon, J.C. & Wcislo, W.T. Eggs of the blind snake, *Liotyphlops albirostris*, are incubated in a nest of the lower fungus-growing ant, *Apterostigma* cf. *goniodes*. *Psyche* **2012,** 1-5 (2012).
269. Martin, M. Natural history of snakes in forests of the Manaus region, central Amazonia, Brazil. *Herpetological Natural History* **6,** 78-150 (1998).
270. Perez, J. & Lehr, E. *Anomalepis aspinosus*. *The IUCN Red List of Threatened Species* **2017,** e.T203190A2761861 (2017).
271. Das, I., Lakim, M., Lim K.K.P. & Hui T.K. New Species of *Anomochilus* from Borneo (Squamata: Anomochilidae). *J. Herpetol.* **42,** 584-591 (2008).

272. Andreone, F. & Luiselli, L. Are there shared general patterns of specific diversity, abundance, and guild structure in snake communities of tropical forests of Madagascar and continental Africa? *Rev. Ecol. Terre Vie* **55,** 215-239 (2000)
273. de Lang, R. & Vogel, G. The snakes of Sulawesi: a field guide to the land snakes of Sulawesi with identification keys. *Edition Chimaira, Frankfurt am Main* (2005).
274. Reynolds, R.G. *et al.* Ecological specialization and morphological diversification in Greater Antillean boas. *Evolution* **70,** 1882-1895 (2016).
275. Stafford, P.J. & Henderson, R.W. Kaleidoscopic tree boas: the genus *Corallus* of tropical America. *Krieger, Malabar, FL* (1996).
276. Acosta Chaves, V. *et al. Corallus ruschenbergerii*. *The IUCN Red List of Threatened Species* **2016,** e.T203211A2762201 (2016).
277. Rodriguez-Robles, J.A., Bell, C.J. & Greene, H.W. Gape size and evolution of diet in snakes: feeding ecology of erycine boas. *J. Zool., Lond.* **248,** 49-58 (1999).
278. Popgeorgiev, G., Tzankov, N., Kornilev, Y., Naumov, B. & Stojanov, A. Species diversity of amphibians and reptiles in the special protected area "Besaparski Ridove", Southern Bulgaria. *Biotechnology & Biotechnological Equipment* **24,** 661-666 (2010).
279. Kreiner, G. The snakes of Europe: all species from west of the Caucasus mountains. *Edition Chimaria, Frankfurt am Main* (2007).
280. Campbell, J.A. & Flores-Villela, O. A new long-tailed rattlesnake (Viperidae) from Guerrero, Mexico. *Herpetologica* **64,** 246-257 (2008).
281. Hammerson, G.A., Frost, D.R. & Gadsden, H. *Lichanura trivirgata*. *The IUCN Red List of Threatened Species* **2007,** e.T63726A12711011 (2007).
282. O'Shea, M. Boas and pythons of the worlds. *New Holland Publishers* (2007).
283. Luiselli, L. *et al.* Diet of the semi-aquatic snake, *Afronatrix anoscopus* (Colubridae) in southern Nigeria. ***Afr. J. Herpetol.*** **52,** 123-126 (2003).
284. Srinivasulu, C., Srinivasulu, B., Deepak, V., Achyuthan, N.S. & Vyas, R. *Ahaetulla pulverulenta*. *The IUCN Red List of Threatened Species* **2013,** e.T172687A1367105 (2013).
285. Ziegler, T., Hendrix, R., Vu, T.N. & Kien, D.N. The diversity of a snake community in a karst forest cosystem in the central Truong Son, Vietnam, with an identification key. *Zootaxa* **1493,** 1-40 (2007).
286. Schargel, W.E. *et al.* A new giant *Atractus* (Serpentes: Dipsadidae) from Ecuador, with notes on some other large Amazonian congeners. *Zootaxa* **3721,** 455-474 (2013).
287. Lubis, I. *et al.* Conservation of herpetofauna in Bantimurung Bulusaraung national park*,* South Sulawesi, Indonesia. *Wildlife Conservation Society, Indonesia Program (2008).*
288. Klug, P.E., Fill, J. & With, K.A. Spatial ecology *of* eastern yellow-bellied Racer *(*Coluber constrictor flaviventris*)* and great plains rat snake *(*Pantherophis emoryi*)* in a contiguous tallgrass-prairie landscape. *Herpetologica* ***67,*** *428-439 (2011).*
289. Ernst, C.H., Ernst, E.M. Snakes of the United States and Canada. *Smithsonian Books Washington, D.C.* (2003).
290. Flores-Villela, O., Köhler , G., Sunyer, J., Townsend, J.H. & Wilson, L.D. *Conophis lineatus*. *The IUCN Red List of Threatened Species* **2013,** e.T63758A3128953 (2013).
291. Capula, M. & Luiselli, L. A tentative review of sexual behaviour and alternative reproductive strategies of the Italian colubrid snakes. *Herpetozoa* **10,** 107-119 (1997).
292. Masood, M.F. Ecological distribution of snakes' fauna of Jazan region of Saudi Arabia. *Egypt. Acad. J. Biolog. Sci.* **4,** 183-197 (2012).
293. Spawls, S.; Howell, K.; Drewes, R.C. & Ashe, J. A field guide to the reptiles of East Africa. *Academic Press* (2002).
294. Casper, G.S. Surveys for the Northern Ring-necked Snake (*Diadophis punctatus edwardsii*) in Northeastern Minnesota: final report. *Minnesota County Biological Survey* (2012).
295. Mousa Disi, A.M. *et al. Eirenis decemlineatus*. *The IUCN Red List of Threatened Species* **2010,** e.T164609A5912176 (2010).

296. Mahlow, K., Tillack, F., Schmidtler, J.F. & Müller, J. An annotated checklist, description and key to the dwarf snakes of the genus *Eirenis* Jan, 1863 (Reptilia: Squamata: Colubridae), with special emphasis on the dentition. *Vertebr. Zool.* **63,** 41-85 (2013).
297. Mousa Disi, A.M. *et al. Eirenis rothii*. *The IUCN Red List of Threatened Species* **2009,** e.T164602A5911399 (2009).
298. Caldwell, J.P. A new Amazonian species of Cryptophyllobates (Anura: Dendrobatidae). *Herpetologica* **61,** 449-461 (2005).
299. Avila, R.W., Ferreira, V.L. & Arruda, J.A.O. Natural history of the south American water snake *Helicops leopardinus* (Colubridae: Hydropsini) in the Pantanal, central Brazil. *J. Herpetol.* **40,** 274-279 (2006).
300. Whitaker, R., Captain, A. & Ahmed, F. Snakes of India. *Draco Books* (2004).
301. Wogan, G. & Chan-Ard, T. *Lycodon capucinus*. *The IUCN Red List of Threatened Species* **2012,** e.T192064A2035298 (2012).
302. *Brown, P. Ecology and vagility of the grass snake Natrix natrix Helvetica. PhD thesis (1992).*
303. Hammerson, G.A. *Nerodia sipedon*. *The IUCN Red List of Threatened Species* **2007,** e.T62239A12583567 (2007).
304. Liu, Y., Ding., L., Lei., J., Zhao, E. & Tang, Y. Eye size reflects habitat and daily activity patterns in Colubrid snakes. *J. Morphol.* **273,** 883-893 (2012).
305. Hammerson, G.A. *Pantherophis obsoletus*. *The IUCN Red List of Threatened Species* **2007,** e.T63864A12715740 (2007).
306. Frost, D.R., Hammerson, G.A. & Gadsden, H. *Phyllorhynchus decurtatus*. *The IUCN Red List of Threatened Species* **2007,** e.T63868A12723163 (2007).
307. Sara, M. & Curtis, S. Field observations of mating behavior in the neck-banded snake *Scaphiodontophis annulatus* (Serpentes: Colubridae). *Rev. Biol. Trop.* **54,** 647-650 (2006).
308. Kim, B.S. & Oh, H.S. A taxonomic reinvestigation of the collared many-toothed snake *sibynophis collaris* gray (reptiles: Serpentes: Colubridae) from Jeju Island, Korea. *Integrative Biosciences* **10,** 121-123 (2006).
309. Seigel, R.A., Ford, N.B. & Mahrt, L.A. Ecology of an aquatic snake (*Thamnophis marcianus*) in a desert environment: implications of early timing of birth and geographic variation in reproduction. *Am. Midl. Nat.* **143,** 453-462 (2000).
310. Sasa, M., Sunyer, J. & Lamar, W. *Trimorphodon quadruplex*. *The IUCN Red List of Threatened Species* **2013,** e.T203336A2764133 (2013).
311. Vogel, G. & David, P. On the taxonomy of the *Xenochrophis piscator* complex (Serpentes, Natricidae). *Proceedings of the 13th Congress of the Societas Europaea Herpetologica*, 241-246 (2006).
312. Hoser, R.T. Divisions within the snake genera *Cylindrophis* Wagler, 1828 (Cylindrophiidae Fitzinger, 1843) and *Anomochilus* Berg, 1901 (Anomochilidae Cundall, Wallach and Rossman, 1993). *Aust. J. Herpetol.* **16,** 31-38 (2013).
313. Allison, A. *Acanthophis rugosus*. *The IUCN Red List of Threatened Species* **2015,** e.T177483A79355114 (2015).
314. Haagner, G.V. & Morgan, D.E. Captive biology of the shield-nosed snake (*Aspidelaps scutatus* intermedius). *Journal of the Herpetological Association of Africa* **40,** 90-94 (1992).
315. Maina, J.N. The morphology of the lung of the black mamba *Dendroaspis polylepis*. *J. Anat.* **167,** 31-46 (1989).
316. Milton, D., Courtney, T., Guinea, M. & Lukoschek, V. *Hydrophis pacificus*. *The IUCN Red List of Threatened Species* **2010,** e.T176712A7288420 (2010).
317. Das, I. A Field Guide to the Reptiles of South-East Asia. *New Holland Publishers, United Kingdom* (2010).
318. Scrocchi, G., Ferreira, V.L., GIraudo, A.R., Avila, R.W. & Motte, M. A new species of Hydrops (Serpentes: Colubridae: Hydropsini) from Argentina, Brazial and Paraguay. *Herpetologica* **64,** 468-477 (2005).

319. Lane, A., Guinea, M., Gatus, J. & Lobo, A. *Laticauda colubrina*. *The IUCN Red List of Threatened Species* **2010,** e.T176750A7296975 (2010).
320. Heatwole, H. Sea snakes (No. Ed. 2). *Krieger Publishing Company* (1999).
321. Stuart, B. & Wogan, G. *Naja kaouthia*. *The IUCN Red List of Threatened Species* **2012,** e.T177487A1488122 (2012).
322. Aubret, F. A comparison of two populations of tiger snakes, *Notechis scutatus occidentalis*. *Master Thesis, University of Poitiers* (2005).
323. Cogger, H. Reptiles and amphibians of Australia. *CSIRO Publising* (2014).
324. O'Shea, M. A Guide to the Snakes of Papua New Guinea. *Independent Publishing Group. Port Moresby, Papua New Guinea* (1996).
325. Guinea, M., Lukoschek, V., Milton, D. & Courtney, T. *Parahydrophis mertoni*. *The IUCN Red List of Threatened Species* **2010,** e.T176772A7301678 (2010).
326. Dudgeon, D. Tropical stream ecology. *Academic Press* (2008).
327. Murphy, J., Brooks, S.E. & Zug, G.R. *Homalopsis buccata*. *The IUCN Red List of Threatened Species* **2010,** e.T176682A7283049 (2010).
328. Vidal, N. Dissecting the major African snake radiation: a molecular phylogeny of the Lamprophiidae Fitzinger (Serpentes, Caenophidia). *Zootaxa* **1945,** 51-66 (2008).
329. Shine, R., Branch, W.R., Harlow, P.S., Webb, J.K. & Shine, T. Biology of burrowing asps (Atractaspididae) from Southern Africa. *Copeia* **1,** 103-115 (2006).
330. Spawls, S. *Duberria lutrix*. *The IUCN Red List of Threatened Species* **2010,** e.T176801A730733 (2010).
331. Graham, G. & Marais, J. A guide to the reptiles of Southern Africa. *Struik Publishers* (2007).
332. Burger, M. *Homoroselaps lacteus*. *The IUCN Red List of Threatened Species* **2017,** e.T110133193A110345288 (2017).
333. Kelly, C.M.R., Barker, N.P., Villet, M.H. & Broadley, D.G. Phylogeny, biogeography and classification of the snake Superfamily Elapoidea: A rapid radiation in the late Eocene. *Cladistics* **25,** 38-63 (2009).
334. Chirio, L. Inventaire des reptiles de la région de Sangarédi (Guinée maritime). *Bull. Soc. Zool. Fr.* **144,** 67-100 (2012).
335. Vidal, N. *et al.* Blindsnake evolutionary tree reveals long history on Gondwana. *Biol. Lett.* **6,** 558-561 (2010).
336. Chan-ard, T., Nabhitabhata, J. & Parr, J.W. A field guide to the reptiles of Thailand. *Oxford University Press* (2014).
337. Rawlings, L.H., Barker, D. & Donnellan, S.C. Phylogenetic relationships of the Australo-Papuan Liasis pythons (Reptilia: Macrostomata), based on mitochondrial DNA. *Australian Journal* of *Zoology* **52,** 215-227 (2004).
338. Das, I. Naturalists’ guide to the snakes of south-east Asia. *London: Beaufoy Books* (2012).
339. Schwartz, A. & Marsh, R.J. A review of the *pardalis-maculatus* complex of the boid genus *Tropidophis* of the West Indies. *Bulletin of the Museum of Comparative Zoology* **123,** 49-84 (1960).
340. Hedges, S.B., Marion, A.B., Lipp, K.M., Marin, J. & Vidal, N. A taxonomic framework for typhlopid snakes from the Caribbean and other regions (Reptilia, Squamata). *Caribb. Herpetol.* **49,** 1-61 (2014).
341. Gower, D.J. Scale microornamentation of uropeltid snakes. *J. Morphol.* **258,** 249-268 (2003).
342. Srinivasulu, C., Srinivasulu, B., Ganesan, S.R. & Vijayakumar, S.P. *Uropeltis ceylanicus*. *The IUCN Red List of Threatened Species* **2013,** e.T172696A1368629 (2013).
343. Srinivasulu, B., Srinivasulu, C., Ganesan, S.R., Vijayakumar, S.P. & Gower, D.J. *Uropeltis ocellatus*. *The IUCN Red List of Threatened Species* **2013,** e.T178233A1528220 (2013).
344. Srinivasulu, C., Srinivasulu, B., Ganesan, S.R. & Vijayakumar, S.P. *Uropeltis rubromaculatus*. *The IUCN Red List of Threatened Species* **2013,** e.T178483A1536535 (2013).
345. Srinivasulu, B., Srinivasulu, C., Ganesan, S.R., Vijayakumar, S.P. & Prabhu, M. *Uropeltis woodmasoni*. *The IUCN Red List of Threatened Species* **2013,** e.T178375A1532051 (2013).

346. Campbell, J.A. & Lamar, W.W. The venomous reptiles of the western hemisphere. *Comstock, Ithaca and London* (2004).
347. Phelps, T. Old world vipers: a natural hstory of the *Azemiopinae* and *Viperinae*. *Edition Chimaira, Frankfurt and Main, Germany* (2010).
348. Lau, M. & Rao, D.-q. *Azemiops feae*. *The IUCN Red List of Threatened Species* **2012,** e.T190641A1955758 (2012).
349. Wogan, G., Grismer, L. & Chan-Ard, T. *Xenodermus javanicus*. *The IUCN Red List of Threatened Species* **2012,** e.T190514A1954501 (2012).
